# Exploring the Stability and Disorder in the Polymorphs of L-Cysteine through Density Functional Theory and Vibrational Spectroscopy

**DOI:** 10.1021/acs.cgd.3c00375

**Published:** 2023-06-29

**Authors:** John Kendrick, Andrew David Burnett

**Affiliations:** University of Leeds, Leeds, LS2 9JT, United Kingdom

## Abstract

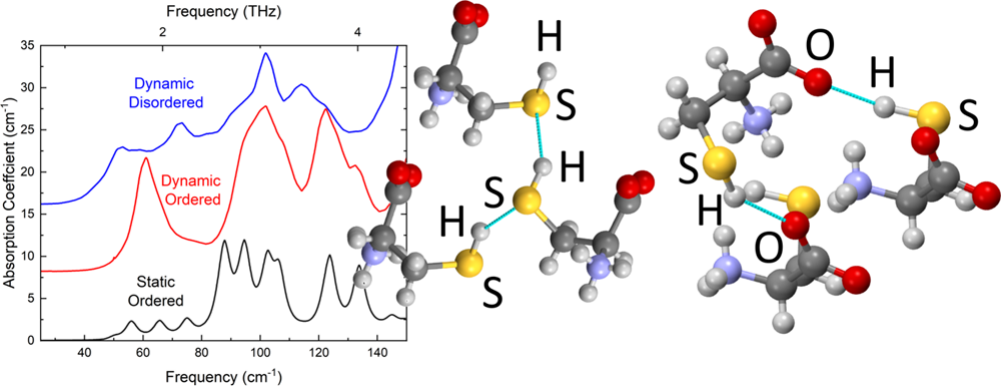

Static and dynamic density functional calculations are reported for the four known polymorphs of l-cysteine. Static calculations are used to explore the relative free energies (within the harmonic approximation) of the polymorphs as a function of pressure. An important feature of the structural differences between the polymorphs is shown to be the dihedral angle of the C–C–S–H bond. It is shown that, by varying this angle, it is possible to move between hydrogen bonding motifs S–H**···**S and S–H**···**O in all four polymorphs. The energetics for dihedral angle rotation are explored, and the barriers for rotation between the hydrogen bonding motifs have been calculated for each polymorph. Two possible models for the experimental disorder observed in Form I at room temperature are explored using both static and dynamic methods; a domain disorder model, where the disorder is localized, and a dispersed disorder model, where the disorder is randomly distributed throughout the crystal. Molecular dynamics calculations show transitions between the two hydrogen bonding motifs occurring in the dispersed disorder model at 300 and 350 K. In addition, molecular dynamics calculations of Form IV also showed the onset of hydrogen bond disorder at 300 K. Calculations of the predicted infrared and terahertz absorption are performed for both the static and dynamic simulations, and the results are compared with experimental results to understand the influence of disorder on the observed spectra.

## Abstract 2

Static and dynamic DFT calculations are used to understand the stability and disorder in the four known polymorphs of l-cysteine. With a focus on Form I, we then explore how the disorder and dynamics influence the measured vibrational spectra. In particular, we show that the terahertz spectra is sensitive to both the lattice dynamics and crystalline disorder.

## 1 Introduction

**Figure 1:**
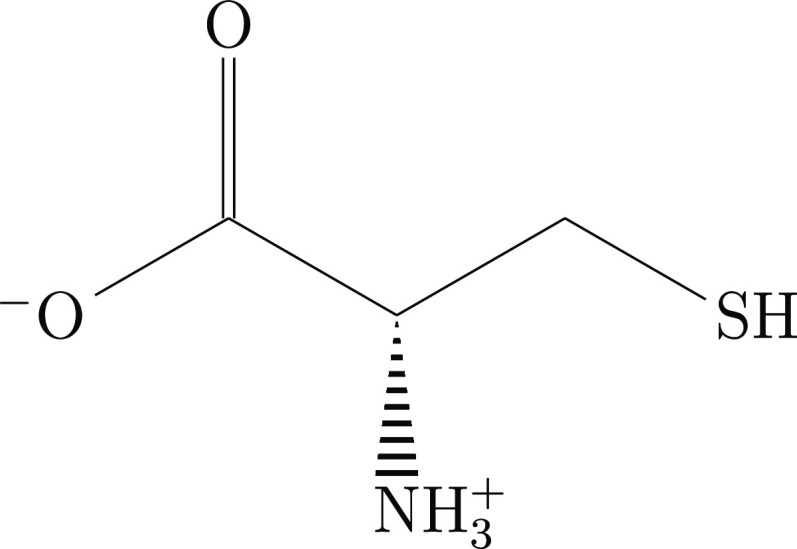
Molecular structure of the l-cysteine zwitterion.

The amino acid l-cysteine (Figure 1) is known to crystallize in two polymorphs at ambient pressure. Form I is orthorhombic (*P*2_1_2_1_2_1_),^1^ with one molecule in the asymmetric unit, and Form II is monoclinic (P2_1_),^2^ with two molecules in the asymmetric unit. The crystal structures of both forms have been studied at ambient temperatures: Form I by X-ray^1^ and neutron diffraction^3^ and Form II by X-ray diffraction.^2,4^ An X-ray diffraction study of Form I at 30 K has also been reported.^5^ In all these studies, l-cysteine is found as a zwitterion (see Figure 1).

**Figure 2:**
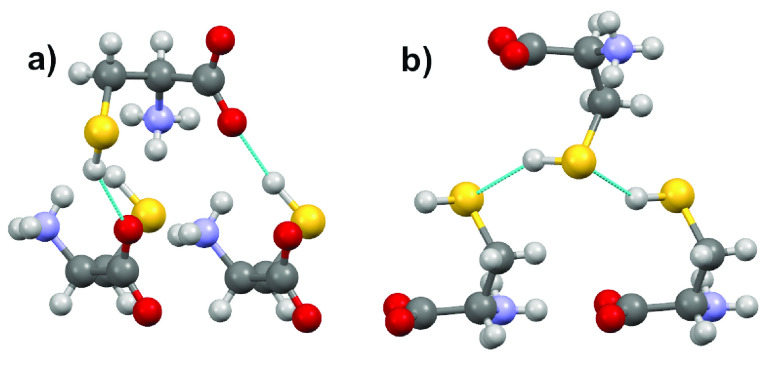
Hydrogen bonding pattern in Form I: (a) S–H**···**O and (b) S–H**···**S.

A low-temperature study (30 K) of Form I^5^ showed that the thiol group is ordered with regular S–H**···**S bonds and anN–C–C–S gauche (g+) dihedral angle of 70.6°. However, a neutron diffraction study at room temperature^3^ showed disorder in the Form I lattice that can be modeled as either involving just the thiol hydrogens or modeled using both sulfur and hydrogen atoms. The disorder arises because the hydrogen of the thiol group can form a hydrogen bond with either another thiol group (as in the Form I 30 K crystal structure) or with an oxygen of the carboxylate anion, as shown in Figure 2. In the neutron diffraction results at room temperature,^3^ the C–C–S–H dihedral angle (τ) was reported to be 77.6° and −85.4° for the SH**···**S and SH**···**O H-bond interactions, respectively. In an X-ray and a polarized Raman study of single crystals of Form I over a range of temperatures from 3 to 300 K^6^ the transition between the ordered, low-temperature structure and the disordered room-temperature structures took place via a series of stages involving activation of the motion of different groups at different temperatures. These results explained the observation of an extended transition observed by calorimetry around 70 K^7^ and also explained why the heat capacity is sensitive to the thermal history. Heat capacities of Form I^8^ showed that there is actually a sharp transition at 76 K that is consistent with an ordering transition of the thiol groups. In order to model the data, it was necessary to use a two-level model: a Debye model for the acoustic phonons and an Einstein model for the optical phonons. The Einstein model is normally used for localized cage phonons and was thought in this case to represent the motion of water molecules trapped in the lattice. The boson peak, which is seen in the low-frequency Raman spectrum of Form I,^9^ can also be explained as the result of a glassy form of disorder in the water molecules distribution.

The observation of both S–H**···**S and S–H**···**O hydrogen bonds in the room-temperature disordered Form I is also reflected in the structure of Form II, which is a monoclinic structure with two independent molecules in the unit cell. The two independent molecules differ in the N–C–C–S dihedral angle, and one molecule shows a gauche conformation (g+) with an angle of 74.4° (which is similar to that seen in Form I). The other molecule is in a trans configuration (t) with an angle of −170.2°. The two molecules also differ in the orientation of the C–C–S–H group, with the first molecule taking part in an S–H**···**O hydrogen bonding pattern and the second taking part in an S–H**···**S pattern.

At pressures above atmospheric pressure two further polymorphs have been determined.^10^ Form III forms above 1.8 GPa and was found to be stable to at least 4.2 GPa. Like Form I, it has a single molecule in the asymmetric unit but with a gauche (g−) N–C–C–S dihedral angle.

Form IV was found on decompression of Form III from 2.6 GPa to 1.7 GPa; like Form II, it has two molecules in the asymmetric unit. Both molecules have gauche N–C–C–S dihedral angles, one g+ and the other g–. The hydrogen atoms bonded to sulfur, however, are not resolved in the X-ray diffraction experiment, and therefore, the work presented here on Form IV has assumed initial hydrogen positions based on those hydrogen bond acceptors (S or O) which are nearest to the donor sulfur atom. This resulted in one of the symmetry unique molecules being involved in an S–H**···**S hydrogen bond and the other with an S–H**···**O hydrogen bond in a similar way to Form II.

Details of the unit-cell dimensions and the pressures and temperatures at which they were determined are provided in Table SI-1 in the Supporting Information (SI). This table also shows the nature of the hydrogen bonding found in each polymorph and illustrates that a significant factor in the differences between the polymorphs and the effect of temperature is the C–C–S–H dihedral angle, which, in turn, dictates whether the hydrogen bonding pattern is SH**···**O or SH**···**S. As can be seen from Table SI-1, Form I has been studied^6^ over a wide range of temperatures, from 30 to 300 K. Some disorder in the hydrogen bonding pattern is seen at all temperatures above 105 K; only the crystal structure of Form I at or below 30 K shows a completely ordered SH**···**S bonding pattern. For the NH**···**O hydrogen-bond motifs, which are also found in all the polymorphs, the article on hydrogen-bond motifs in hydrophobic amino acids provides a summary of the common types found in these systems.^11^

Previous density functional theory (DFT) molecular dynamics calculations have been performed to understand the terahertz spectrum of l-cysteine, which was determined at 77 K and 298 K.^12^ Static solid-state energy calculations^5^ indicated that the Form I polymorph with S–H**···**S intermolecular interactions is only 4.1 kJ mol^–1^ lower in energy than a similar crystal structure but with S–H**···**O intermolecular bonding, confirming the likelihood of disorder as observed experimentally.

Previous spectroscopic work reported on l-cysteine includes angle-dependent terahertz time-domain spectroscopy of single crystals and molecular quantum calculations of the vibrational modes of the cysteine molecule in its zwitterion form.^13^ This work showed a peak in the powder spectrum at 46.06 cm^–1^, which was not observed in the single crystal spectrum. A complete assignment of the vibrational spectra of both Form I and Form II has been reported by Parker^14^ using infrared, Raman, inelastic neutron scattering, and solid-state quantum mechanical calculations. Form II (monoclinic) is shown to have a short SH**···**HN nonbonded interaction just less than twice the van der Waals’ radius of hydrogen. Mink et al.^15^ have reported infrared spectra of Form I at room temperature in its various deuterated forms. The S–H and S–D stretching vibrations were found to be local modes, which were not sensitive to the deuteration of the methylene or amino groups. The force constants of the S–H bond indicated that those involved in the SH**···**S hydrogen bonding network were stronger than those involved in the SH**···**O network. Terahertz measurements and static DFT calculations have been reported by Ren et al.,^16^ who found six peaks in the low-temperature spectrum Form I below 120 cm^–1^ and three peaks in the room-temperature spectrum.

Form II has been studied by a multitechnique approach,^17^ including incoherent elastic and inelastic neutron scattering, DSC and X-ray diffraction over a range of temperatures from 2 to 300 K. A dynamic transition at 150 K was observed and ascribed to a crossover for harmonic to anharmonic motion. An anomaly was also seen at 240 K in the unit-cell parameters of the crystal. The pressure dependence of the Raman scattering of Forms I and II has been studied^18^ to get an understanding of the dynamics of the side chain on the phase transitions observed in this material. For Form II, phase transitions were observed at ∼2.9 and ∼3.9 GPa, which were completely reversible, indicating no radical change to the molecular conformation under pressure. Form I was observed to behave differently, indicating substantial conformational changes with pressure.

The pressure dependence of the Raman scattering of Form I has also been examined up to 20.2 GPa.^19^ Two structural transitions were observed; one between 1.8 and 3.6 GPa and the other between 6.9 and 7.8 GPa. The first is probably associated with interconversion between the two types of S–H hydrogen bonds and the second with the breakdown of these hydrogen bonds. A further three transitions were suggested by the data. The temperature-induced phase transition of Form I has been studied using DSC and powder X-ray diffraction^20^ in the temperature range from 268 K to its melting point (493 K).^9^ Two phase transitions were seen at 464 and 480 K.

Terahertz spectroscopy is an ideal method to investigate the effect of the different intermolecular interactions occurring in this system, as it probes the lattice vibrations of the crystal. However, calculations of the absorptions at these low frequencies are known to be difficult to perform and sensitive to the details of the calculation.^21^ In this paper, we report theoretical calculations using both static and molecular dynamic DFT calculations to examine the nature of the hydrogen bonding in all the polymorphs of l-cysteine in an effort to shed further light on the nature of the disorder, which occurs in this material.

## 2 Methods

### 2.1 Static Calculations

Static DFT calculations were performed with the VASP package^22^ using the Perdew–Burke–Ernzerhof (PBE) functional^23^ and the Projector Augmented Wave (PAW) pseudopotentials^24^ distributed with VASP 5.4.1. Dispersion corrections were included using the Grimme DFT-D3 method^25^ with Beck–Johnson damping.^26^ Full details of the settings used are provided in Section S2 of the SI. For full geometry optimizations of the unit-cell and the molecular geometries, the default optimizers within VASP were used. For those calculations which required constraints, the GADGET optimizer^27,28^ was used, which has an interface to the VASP package.

### 2.2 Molecular Dynamics

Dynamic DFT calculations using Ab Initio Molecular Dynamics (MD) were performed with the CP2K package.^29^ As was used in VASP, all calculations employed the PBE exchange-correlation potential, along with the GD3/BJ dispersion correction. The Molopt double-ζ valence plus polarization basis sets^30^ were used with GTH PBE pseudopotentials.^31^ Full details of the protocols used for the NPT and NVT simulations are reported in Section S4 of the SI.

Some of the static and dynamical calculations employed supercells, a full description of which are given in the Section S6 in the SI. A supercell is indicated by the use of the “SC” or “DC” designation, followed by the polymorph, followed by the number of molecules in the cell and optionally a letter to distinguish between cells. Thus, SCII8 refers to a supercell of Form II with 8 molecules in the supercell. Where there is some disorder in hydrogen bonding pattern, the designation “DC” is used instead of “SC” and, therefore, DCI32 would refer to a disordered cell of Form I containing 32 molecules. All calculations using supercells were performed without symmetry constraints.

### 2.3 Infrared and Terahertz Spectral Calculations

Two different approaches were used to calculate the infrared absorption spectra of all polymorphs of cysteine. From the static calculations using VASP, the Born charges and phonon frequencies at the Γ point were determined, enabling the calculation of the infrared and terahertz spectrum by the PDielec package,^21,32,33^ which assumes a powdered crystalline material of spherical morphology (10% by volume) supported in a PTFE matrix, which was represented using the Maxwell–Garnett effective medium approximation. Unless otherwise stated, a line broadening of 5 cm^–1^ was used in all calculations of the terahertz and infrared spectra from the static DFT calculations. For unit cells with 4 or less molecules in the cell, the calculation of the phonon frequencies was performed using density functional perturbation theory implemented in VASP. For larger cells the Phonopy^34^ package was used with VASP providing the energies and forces to calculate the dynamical matrix numerically with a summary of parameters used included in the Section S3 in the SI. VASP was used to calculate the Born charges of the cells to determine the infrared intensity of the modes for all cells, independent of size.

For molecular dynamics calculations, fluctuations in dipole moment were used to calculate the absorption spectrum. Travis^35,36^ calculates the molecular dipole moment fluctuations by partitioning the electron density using the Voronoi algorithm, but we were concerned that the low-frequency vibrations below 400 cm^–1^ might not be treated well by such a molecular algorithm. Instead, we sampled the cell dipole every 0.5 ps and calculated the dielectric permittivity of the cell from the dipole moment correlation function.^37^ Full details of the method used are provided in Section S5 of the SI and the scripts used to perform the calculation are available.^38^ This approach is subject to more noise than the methods used within the Travis package but should capture the correlations between molecules that Travis does not consider, which may be important for terahertz spectral prediction. In Section 8.3 of the SI, we compare the results obtained for the absorption spectrum using both methods and this shows reasonable agreement between them for frequencies above 400 cm^–1^ but larger spectral differences below. The Travis program was used to analyze the MD trajectories to determine the dihedral angle distribution functions.

## 3 Results

### 3.1 Static Calculations

In the following sections we report the results of the static calculations performed on all four forms of l-Cysteine. In particular the effects of pressure, the energetics for rotation of the C–C–S–H dihedral angle and the influence of disorder will be explored.

#### 3.1.1 VASP Unit-Cell Optimisations

Each experimental polymorph crystal structure was optimized using VASP at a pressure of 0.0 GPa and at the experimental pressure used to determine the structure. The results for the zero pressure optimizations are reported in Tables SI-12 and SI-13 in the SI. The calculated unit-cell dimensions under the experimental pressure conditions are shown in Table 1. The percentage deviations from the experiment are reported in Table SI-11 in the SI. All deviations are <1.6% of the experimental value, and there seems to be a general tendency to calculate a smaller unit-cell volume than that observed experimentally, which is possibly owing to temperature effects and highlighted by Form I where the difference between the volume of the calculated cell and that recorded experimentally at 30 K is only 0.91%.

**Table 1:** DFT Optimized Unit-Cell Parameters for Polymorphs at Experimental Pressures

polymorph	*P* (GPa)	*a* (Å)	*b* (Å)	*c* (Å)	**α** (°)	**β** (°)	**γ** (°)	volume (Å^3^)	energy (eV)
Form I	0.0	8.087	11.900	5.421	90.0	90.0	90.00	521.65	–323.555
Form II	0.0	9.438	5.199	11.218	90.0	109.0	90.00	520.48	–323.464
Form III	2.6	7.949	10.511	5.348	90.0	90.0	90.00	446.83	–322.858
Form III	4.2	7.874	10.315	5.288	90.0	90.0	90.00	429.46	–322.456
Form IV	1.7	8.073	5.402	10.927	90.0	95.8	90.00	474.08	–323.157

The lattice energies quoted in Table 1 are not strictly comparable, as some of the calculations are performed under different pressure conditions. However, the calculations at zero pressure reported in Tables SI-12 and SI-13 are all performed at the same pressure and the ranking of the stability of the polymorphs is found to be Form I > Form II > Form IV > Form III.

#### 3.1.2 Effect of Pressure on Geometry

**Figure 3:**
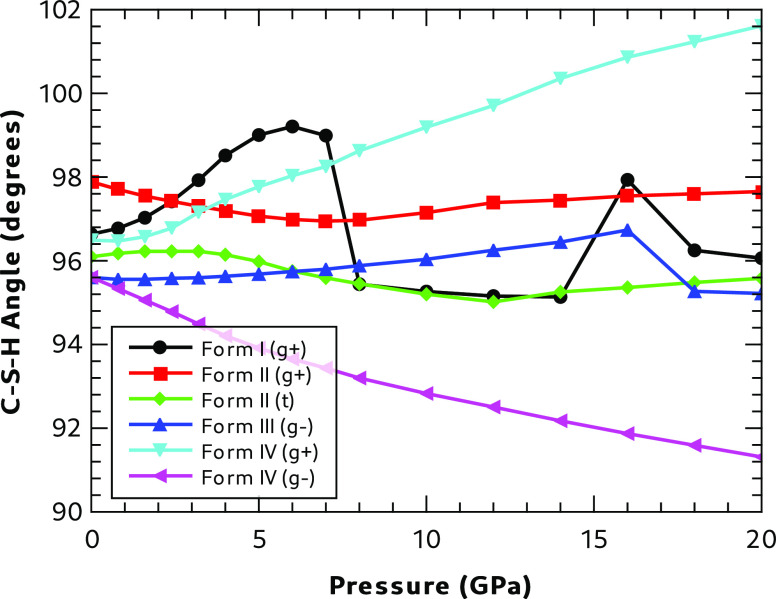
Effect of pressure on the C–S–H angle. (The N–C–C–S conformation is shown in brackets.)

Analysis of the changes in unit cell volume and molecular geometry as a result of systematically changing the pressure from 0 to 20 GPa (see Section 7.3 in the SI for details) showed that, apart from Form I, there was a smooth change in the geometric parameters over the full pressure range. Form I, however, showed discontinuities in several geometric parameters but especially the C–S–H bond angle (Figure 3). This increased from an initial value of 96.8° to 99.2° at ∼7 GPa, when it dropped to below 96°. This structural change may be consistent with that observed between 6.9 and 7.8 GPa by a Raman study of Form I,^19^ where it was attributed to a possible breaking of the sulfhydryl hydrogen bonds. These results are also consistent with the Raman study of Forms I and II,^18^ which indicated that Form II behaved reversibly under pressure, while Form I did not; indicating substantial irreversible conformational changes in Form I as the pressure is altered. A sudden change in angle also occurs at 16 GPa for both Forms I and III.

#### 3.1.3 Effect of Pressure on Polymorph Stability

The free energies of each polymorph as a function of pressure were calculated up to a pressure of 7 GPa. The calculations were performed using the Phonopy package^34^ and details of the calculation are presented in the Section S7 in the SI. The calculation assumes that the free energy can be estimated from the zero-point energy (ZPE) and the entropic contributions of the phonons using the harmonic approximation.

**Figure 4:**
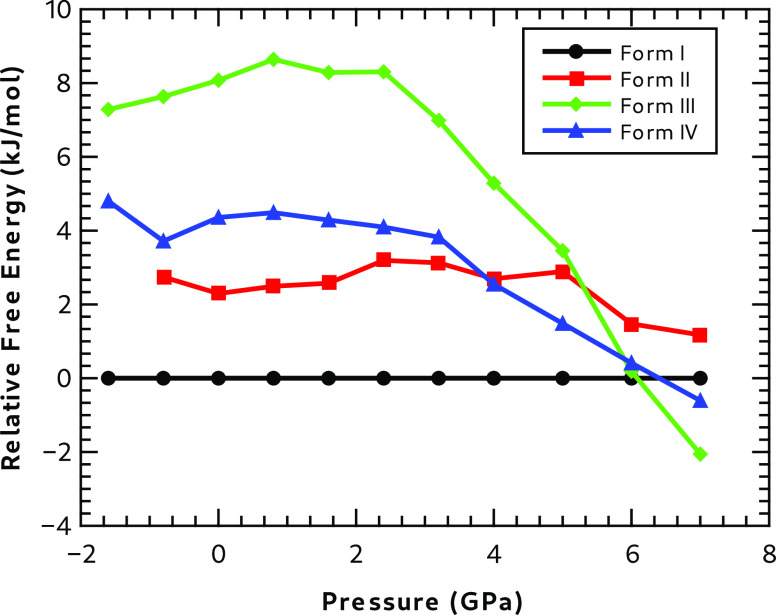
Helmholtz free energies at 300 K relative to Form I.

Figure 4 shows the quasi-harmonic Helmholtz free energies, including ZPEs for each polymorph, calculated as a function of pressure relative to the free energy of Form I.

Figure 4 shows that Forms III and IV become more stable than Forms I and II as the pressure increases above 6 GPa. Experimentally these polymorphs are indeed observed at higher pressures as can be seen in Table SI-1, though the experimental pressures at 2.6 and 1.7 GPa for polymorphs III and IV, respectively, are considerable less than the 6 GPa found by calculation. The effect of including the quasi-harmonic approximation is quite small. Section S7 contains results with and without the phonon contributions, and it is clear that the main effect arises owing to the effect of pressure on the internal energy of the lattice. Figure SI-6 in the SI shows that, if anything, the quasi-harmonic approximation moves the crossing point for the stabilities of polymorphs III and IV to higher pressures.

The quasi-harmonic approximation may not be appropriate in this system, as the mobility of the hydrogen bond network in Form I indicates that large amplitude anharmonic motion may be present at room temperature. Another possible contribution to the experimental polymorph stability not considered in the computational work is the contribution from disorder of the water content in these hygroscopic crystals. As well as giving rise to additional electronic and vibrational contributions to the free energy, the disorder will also give rise to a configurational entropy contribution, which we expect to be larger for lower pressures and smaller for smaller unit cells. The effect of this would be to make this term important in determining the crossing points in the relative stability of the polymorphs, as a function of pressure.

#### 3.1.4 Energetics for C–C–S–H Dihedral Angle Rotation

To understand the mobility of the C–C–S–H group, a series of calculations were performed on each polymorph unit cell (ignoring symmetry), rotating one of the C–C–S–H bond angles in a systematic way from −180° to +180° in steps of 10° and constraining its value while optimizing all the other molecular, geometric variables. Each unit cell contains 4 molecules, and only one of these undergoes a constrained dihedral angle optimization at a time. However, in Forms II and IV, there are two molecules in the asymmetric unit, so there are two molecules to consider when exploring the torsional energetics of C–C–S–H. During the optimization the unit-cell dimensions were held fixed at the dimensions determined by full optimization of the experimental unit cell and its contents at zero pressure.

**Figure 5:**
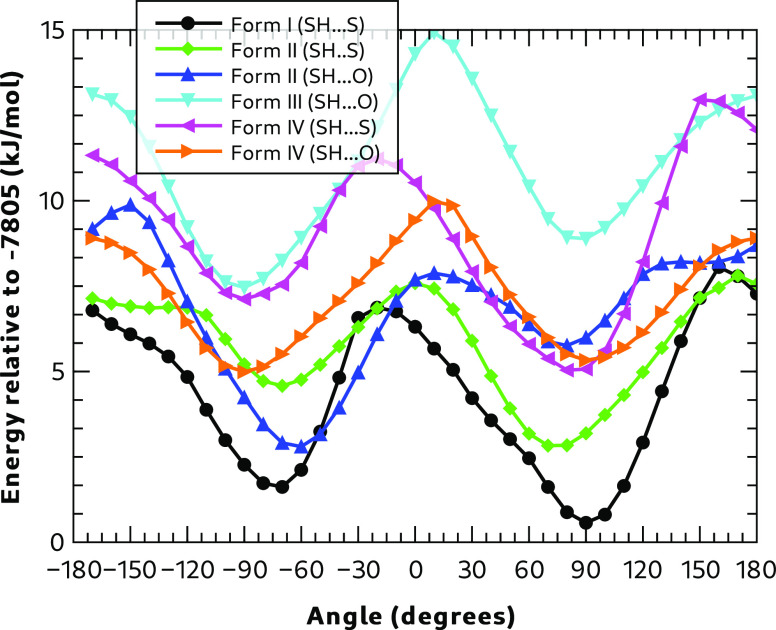
C–C–S–H dihedral angle energies. (The initial hydrogen bonding pattern for the rotating C–C–S–H bond is shown in brackets.)

Figure 5 summarizes the results of these calculations. All curves show a similar pattern with minima between −90° to −60° and +60° to +90°. All curves also show maxima around 0 and 180°.

For Form I, which has an SH**···**S hydrogen bonding pattern as its most stable structure, the other minimum, consistent with SH**···**O bonding, occurs 1.0 kJ mol^–1^ higher in energy at an angle of about −70°. The barrier to interconversion is ∼6.3 kJ mol^–1^ at −20°. Note that this is not a barrier height consistent with a transition state; it is rather the barrier for the particular path that has been chosen.

Form II has two different environments for the C–C–S–H group. The one involved in an SH**···**S hydrogen bond in the fully optimized structure can rotate from 75° to a minimum at −70° associated with an SH**···**O hydrogen bond, which is 1.9 kJ mol^–1^ higher in energy with a barrier of 4.8 kJ mol^–1^. The other group (involved with an SH**···**O hydrogen bond in the optimized structure) has a minimum at −63°, with the SH**···**S configuration 3.0 kJ mol^–1^ higher in energy with a barrier of5.0 kJ mol^–1^.

Form III has its lowest energy minimum near −90° and is stabilized by an SH**···**O hydrogen bond. The higher energy minimum is 1.4 kJ mol^–1^ higher in energy with an angle of 88°. The lowest barrier for interconversion is 5.7 kJ mol^–1^ at a dihedral angle of −180°.

Like Form II, Form IV has two different C–C–S–H groups. The group involved with an SH**···**O hydrogen bond in the optimized structure has a higher energy minimum at 80° only 0.2 kJ mol^–1^ higher than its global minimum, with a barrier at 180° of 3.9 kJ mol^–1^. The other group, which is part of a SH**···**S hydrogen bond in the optimized structure has another minimum 2.1 kJ mol^–1^ higher in energy with a barrier height of 6.2 kJ mol^–1^.

Given these results and the knowledge that Form I shows a disordered structure at room temperature, it is very conceivable that the other polymorphs will show similar disorder at increased temperatures. However, these calculations were performed using unit cells optimized at zero pressure, and the energetics will differ at the higher pressures required to stabilize Forms III and IV.

#### 3.1.5 Models of Form I Disorder

In order to further investigate the nature of the disorder observed in Form I at high temperature, a series of unit cells were created to represent the type of disorder that may be found. X-ray crystallography and neutron scattering indicate that, at room temperature, about half of the S–H groups are involved with SH**···**S hydrogen bonds and about half with SH**···**O hydrogen bonds.^5^ These different environments may be dispersed randomly throughout the crystal or they may be localized in domains.

##### 3.1.5.1 Domain Disorder Model

To study domains of the SH**···**O hydrogen bonding in Form I, a new unit cell was created starting with the low-temperature crystal structure (which has only SH**···**S hydrogen bonding) by altering every molecule’s C–C–S–H dihedral angle from 77.6° to −85.4°, thereby creating only SH**···**O hydrogen bonds in the unit cell. This unit cell was then optimized maintaining the space group symmetry (*P*2_1_2_1_2_1_). In what follows, “Form I” will refer to the unit-cell with the SH**···**S motif, while “Form I (SH**···**O)” will be used to refer to the unit cell with the other hydrogen bonding motif. As was found previously,^5^ the experimentally observed low-temperature S–H**···**S hydrogen bonding pattern was found to be more stable than the S–H**···**O pattern. The difference in stability of the two hydrogen-bonding patterns was 2.4 kJ mol^–1^ and 5.4 kJ mol^–1^ for the nondispersion and GD3/BJ dispersion corrected methods, respectively, in favor of the experimentally observed SH**···**S hydrogen bonding pattern. These results compare with 4.1 kJ mol^–1^ reported previously.^5^ The hydrogen bonding patterns established at the outset of the calculations were maintained throughout the unconstrained optimization procedure, indicating that there is a barrier for the relaxation of the S–H**···**O to the S–H**···**S hydrogen bonding pattern. The calculated C–C–S–H dihedral angles are reported in Table 2 and can be compared with the experimental value for the ordered crystal observed at 30 K and for the disordered crystal measured at 293 K.^5^

**Table 2:** Comparison of Experimental and Calculated Form I C–C–S–H Dihedral Angles

description	H-bond	τ (deg)
calculated	SH**···**S	88.3
calculated	SH**···**O	–69.2
experimental^a^ (30 K)	SH**···**S	87.0
experimental^a^ (293 K)	SH**···**S	74.2
experimental^a^ (293 K)	SH**···**O	–67.3

a Experimental data taken from ref (5).

##### 3.1.5.2 Dispersed Disorder Model

As has been mentioned previously, experimental observation indicates that, at room temperature, the population of S–H**···**S and S–H**···**O hydrogen-bonding motifs is roughly equal.^5^ This disorder may be dispersed randomly throughout the crystal and in order to simulate this, supercells, based on the experimental 295 K unit-cell^6^ (CSD code LCYSTN36), were created with *P*1 space-group symmetry. In each cell, half of the C–C–S–H dihedral angles were set to −78.4° and the other half to 87.0°. The choice of which dihedral angle to change was selected at random. A variety of supercells were investigated with between 8 and 32 molecules in the cell. For each supercell size, two unit cells were created with a different distribution of dihedral angles in the cell and the atomic positions optimized using DFT. Comparison of the results of calculating the infrared absorption spectrum using Phonopy and PDielec indicated that the calculated spectrum of the two supercells had converged when the number of molecules in the cell reached 32. The largest unit cells, DCI32a and DCI32b, were used in the calculation of the infrared and terahertz spectral calculations reported later. For all subsequent calculations, the unit-cell dimensions and the atomic positions were first fully optimized. One of these cells, DCI32a, was also used as the starting point for the CP2K MD calculations, where it will be referred to as DCI32. Details of the static spectra calculated for all the supercells is available in Section 7.8 in the SI.

### 3.2 Molecular Dynamics Calculations

In the following sections we report the results of the dynamic calculations performed on all four forms of l-Cysteine. with a particular focus on how the dynamics of the material influences the distribution of the C–C–S–H dihedral angle.

#### 3.2.1 NPT Calculations

Supercells with 8 molecules in the supercell were used in NPT molecular dynamics calculations for each polymorph. For the calculations on the dispersed disorder model for Form I, supercell DCI32 was used. The average cell dimensions and the temperatures and pressures of the simulations are listed in Table 3. Figures showing the fluctuations in the cell dimensions are available in Figures SI-36–SI-42 of the SI. The time evolutions of the dihedral angle are shown in Section 8.2 in the SI.

**Table 3:** Average Super-Cell Dimensions Calculated by MD

cell	number of molecules	*T* (K)	*P* (GPa)	*a* (Å)	*b* (Å)	*c* (Å)	**α** (°)	**β** (°)	**γ** (°)
SCI8	8	300.0	0.0	10.880	8.194	12.127	90.0	90.0	90.0
SCII8	8	120.0	0.0	9.370	10.456	11.299	90.0	104.6	90.0
SCIII8	8	300.0	2.6	10.858	8.073	10.761	90.0	90.0	90.0
SCIV8	8	300.0	1.7	8.121	10.924	11.115	89.8	94.3	90.0
									
DCI32	32	88.0	0.0	12.163	16.165	21.717	90.2	90.0	90.0
DCI32	32	300.0	0.0	12.178	16.243	21.700	90.0	90.0	90.0
DCI32	32	350.0	0.0	12.203	16.298	21.729	90.0	90.0	90.0

#### 3.2.2 Dihedral Angle Distributions

**Figure 6:**
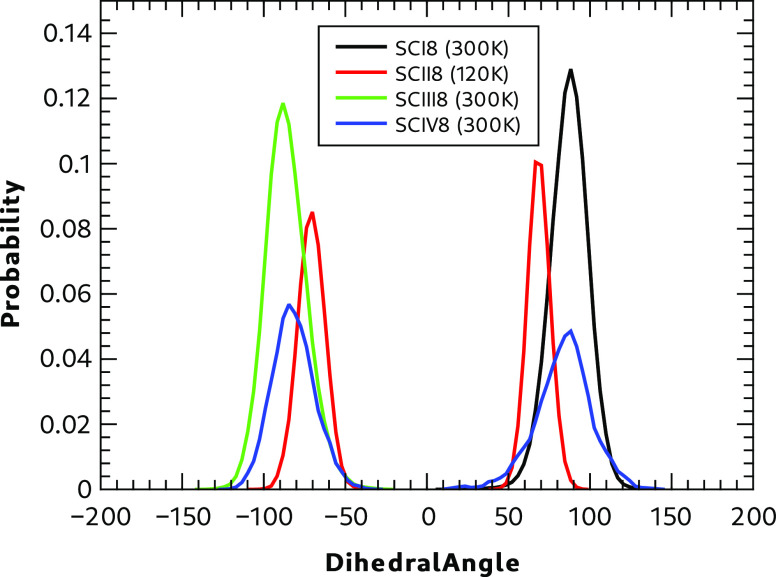
Polymorph dihedral angle distribution functions.

Analysis of the C–C–S–H dihedral angle distribution in the ordered models of Forms I, II, III, and IV during the NVT production phase of these molecular dynamics calculations is reported in Figure 6.

Forms II and IV show two peaks in the distribution functions consistent with SH**···**O and SH**···**S hydrogen bonding in the lattice. Of the two polymorphs, Form IV shows a wider distribution, indicating a more mobile dihedral angle. Forms I and III show peaks consistent with a single hydrogen bond type; SH**···**S for Form I and SH**···**O for Form III. Form II shows a slightly narrower distribution than the other polymorphs reflecting the temperature of 120 K at which the simulation was performed.

**Figure 7:**
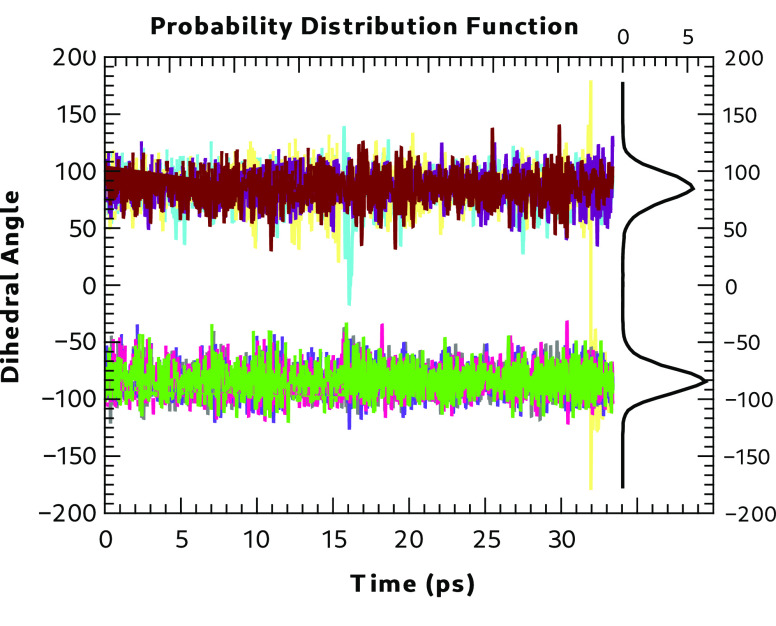
Form IV time evolution dihedral distribution function at 300 K.

For all but Form IV, no changes in the nature of the hydrogen bonding pattern associated with the C–C–S–H dihedral angle were observed. Although Form I has significant excursions of the dihedral angle from its mean value (see Section 8.2 in the SI for further details). The dihedral angle distribution observed for Form IV is shown in Figure 7. The figure shows the time evolution of the eight dihedral angles and their probability distribution (with the integrated area normalized to 100). A large excursion of a dihedral angle takes place at ∼16 ps, but no flip occurs of the bonding pattern. However, at 32 ps, a single C–C–S–H dihedral angle flip occurs, resulting in a change in the hydrogen bonding pattern. These results and those from the static calculations on the energetics of dihedral angle rotation indicate that polymorphs other than Form I are also very likely to show disorder in the hydrogen bonding patterns at higher temperatures.

#### 3.2.3 Dihedral Angle Distribution in the Form I Distributed Disorder Model

**Figure 8:**
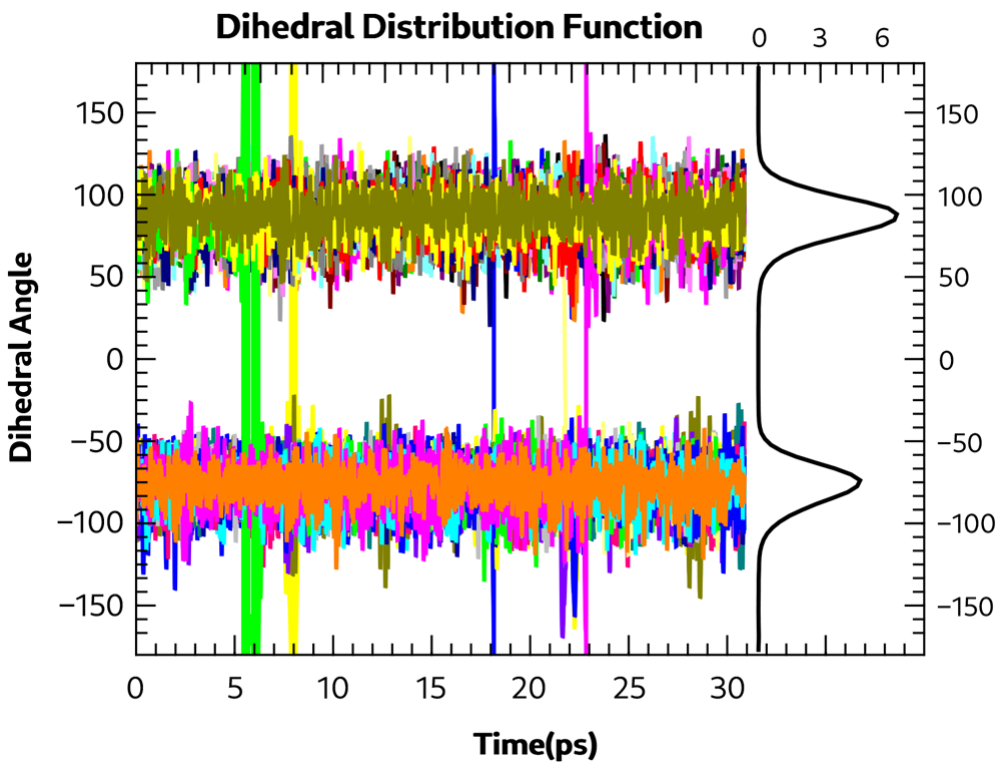
DCI32 dihedral angle distribution of C–C–S–H at 300 K.

Analysis of the C–C–S–H dihedral angle distribution during the NVT production phases of simulations of DCI32 at 300 K is summarized in Figure 8. The figure shows the values of the dihedral angle for each of the 32 molecules in the unit cell, as a function of temperature, with each molecule represented by a different color. In total, there are 5 flips. The first occurs at just above 5 ps. It appears broad because the dihedral angle of this molecule moves to +180°/–180° for a period of time, and this causes it to appear as though there are many flips occurring. The molecule spends over 0.5 ps in this metastable state before committing to a flip from an SH**···**S to an SH**···**O bonding pattern. The transition at 8 ps starts with a molecule with a SH**···**O bonding pattern, which flips, but then shortly later flips back, maintaining its original hydrogen bonding. At 18 ps, there is a flip from SH**···**O to SH**···**S. The other two flips occur at 21.8 and 22.8 ps with flips from SH**···**S and SH**···**O hydrogen bonding patterns, respectively. The right-hand side of the figure shows the distribution function of all the dihedral angles. There are broad peaks at ∼90° and ∼−80°, consistent with a slightly enhanced probability of SH**···**S over SH**···**O, respectively. Similar results are obtained for the simulation at 350 K, and these can be found in the Section S8. MD calculations of the disordered model at 88 K showed no large excursions of the torsion angle.

### 3.3 Calculating Vibrational Spectra

In the following sections, we will explore how the different static and dynamic models described previously can be used for calculating the infrared and terahertz spectra and how these methods influence the resulting calculated spectral properties. As each polymorph has a unique crystal packing structure, we focus our discussion on the low-frequency infrared response (below 150 cm^–1^, which is dominated by external modes of motion) and on the high frequency response associated with the S–H stretching vibration, as these are the regions most sensitive to polymorphism, dynamics, and disorder, although calculated spectra for all spectral regions are available in the SI (static in Section S7 and dynamic in Section S8).

#### 3.3.1 Calculated Terahertz Spectra

**Figure 9:**
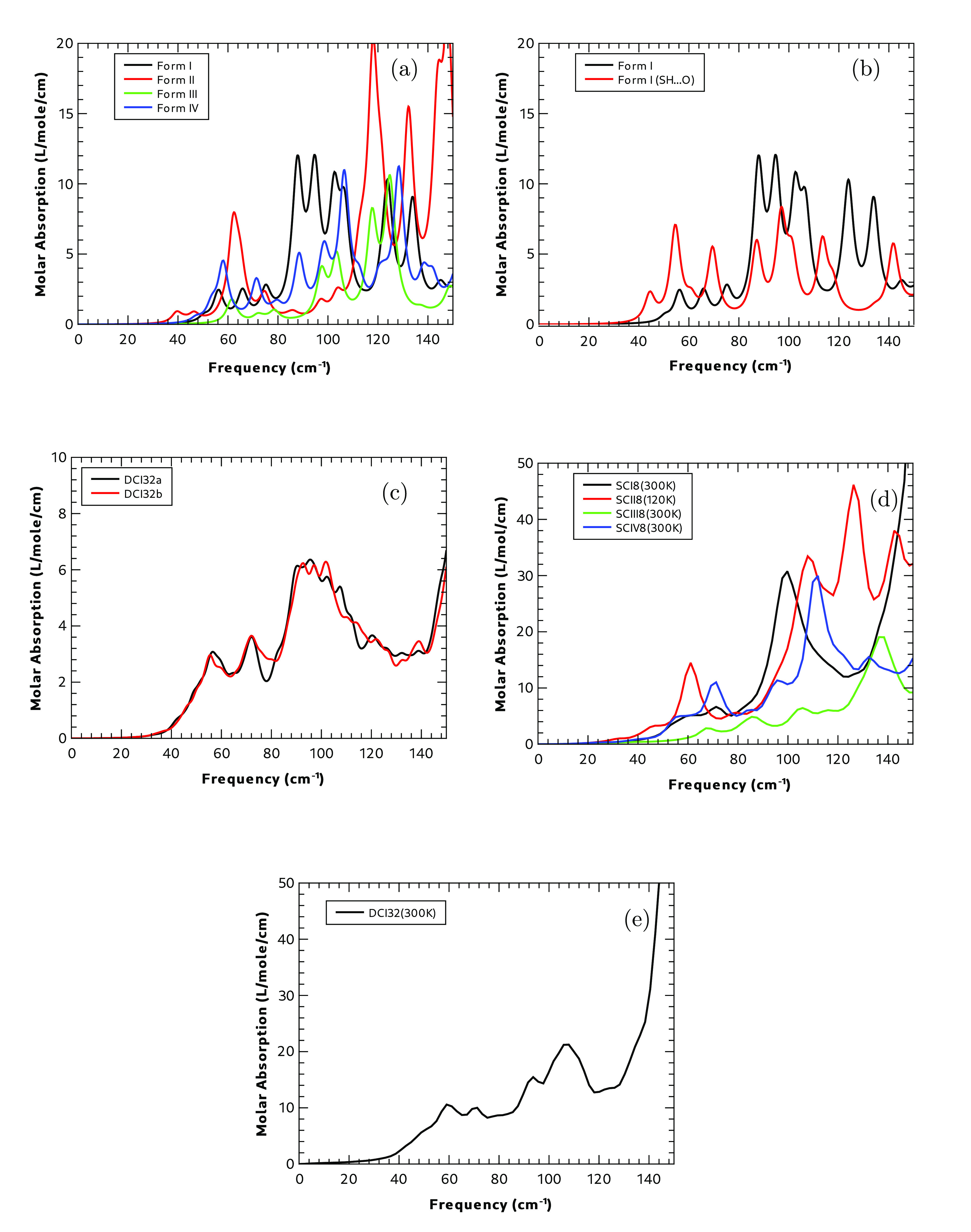
Calculated THz spectra (a) using static calculations of all four polymorphs, (b) using static calculations for Form I SH**···**S and SH**···**O hydrogen-bonding motifs, (c) using static calculations of the Form I dispersed disorder models containing 32 molecules, (d) using the dynamic calculations of the four polymorphs, and (e) using dynamic calculations of the disordered supercell (DCI32a) of Form I.

Figure 9 shows a summary of the terahertz spectral calculations for various static and dynamic calculation procedures. Overall, the key differences are that the static calculations generally predict a much lower infrared intensity (in some cases by a factor of more than four), compared to the equivalent dynamics calculation, while the inclusion of dispersed disorder tends to broaden spectral features, which reduces the definition of specific peaks when compared to the calculations performed on more-ordered systems.

Figure 9a shows the static spectrum of each of the four polymorphs. Each polymorph has a unique crystal packing, which should be reflected in the low-frequency infrared response, and this seems to be the case, with very different features for each of the four polymorphs. Form II has the lowest frequency absorption feature, below 40 cm^–1^, and it shows two prominent absorptions just above 60 and 120 cm^–1^. Both Forms I and IV show a low-frequency absorption peak at ∼56 cm^–1^, with weaker transitions at lower frequencies broadening the absorption peaks. Form III shows less absorption in the terahertz region than any of the other polymorphs, while Form II has the most intense spectral feature.

In the case of domain disorder, the experimental infrared and terahertz spectra of Form I are likely to be a mixture of the absorptions arising from the two possible H-bonding motifs. Figure 9b shows the predicted absorption spectra for the two unit cells described in section 3.1.5.1. As can be seen in the figure, the predicted terahertz spectra from the two hydrogen bonding motifs are quite different. In particular, the SH**···**O bonding pattern shows a low-frequency peak below 50 cm^–1^ and a much stronger peak just above 55 cm^–1^.

Figure 9c shows the calculated terahertz absorption spectrum for the static DFT calculations performed using the disordered supercells DCI32a and DCI32b. There is significant agreement between the spectra calculated from both supercells to suggest that these cells are large enough to represent the disorder in the system. Section S7.8 in the SI contains details of the terahertz spectra calculated for all the disordered supercells (DCI8, DCI16, and DCI32) used in this work and shows a lack of agreement with the smaller supercells. Both the terahertz and high-frequency ranges are sensitive to the hydrogen bonding patterns that are found in each cell. In the spectra of the disordered supercells, it can be seen that these show features common to both the domain disordered systems shown in Figure 9b, although this is not a simple addition of these spectra but rather a new spectrum showing some similarity to both.

**Figure 10:**
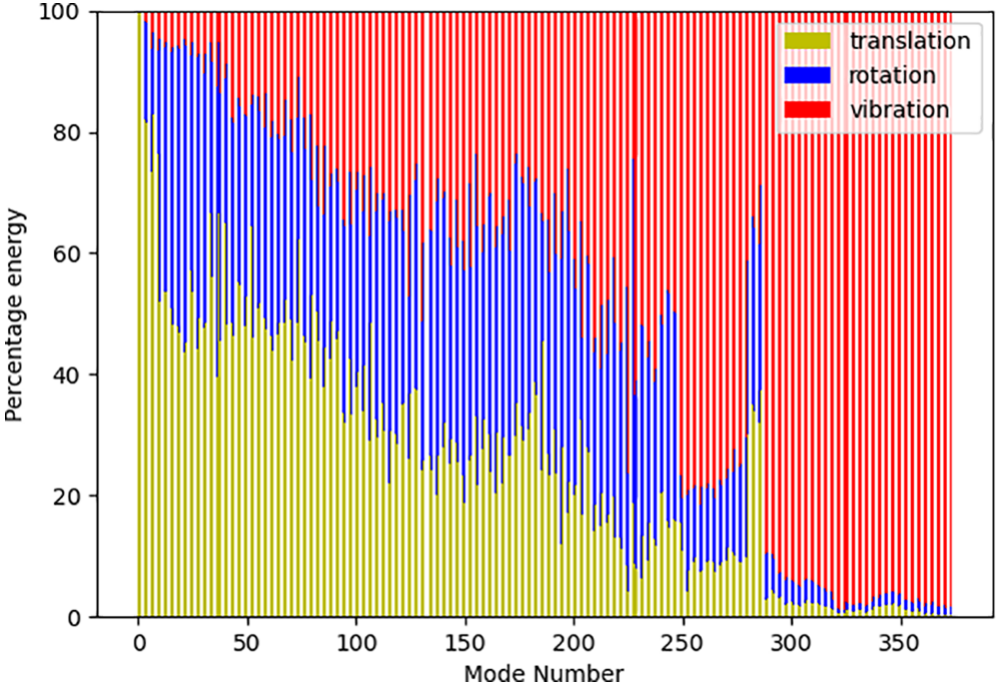
Internal and external mode contributions to the low-frequency vibrations of DCI32a.

Analysis of the internal and external contributions to the modes for both supercells is very similar. Figure 10 shows that percentage contribution from translational, rotational, and vibrational (internal) motion^39^ to the first 300 modes of vibrations of DCI32a. It can be seen that as the frequency increases, there is a steady decline in the contributions from the external rotation and translation contributions. Mode 200 in this figure vibrates at ∼140 cm^–1^ and still more than 60% of the energy in the vibration is coming from external modes. Almost pure internal modes occur above mode 300, with a frequency of 290 cm^–1^.

Figure 9d shows the results of calculating the absorption in the terahertz frequency range of all polymorphs using dynamic calculations on supercells where the spectra is determined from the cell dipole moment fluctuations of the MD trajectory. In the terahertz region, even with the narrow settings for the dipole moment correlation windowing function, the absorption peaks are broad, with Form II showing a weak absorption at ∼45 cm^–1^. All polymorphs except Form III show strong absorption at ∼100 cm^–1^, while Form III shows little absorption until above 130 cm^–1^. Form II shows the strongest absorption of all of the spectra.

It is insightful to compare these calculations with the equivalent static calculations using PDielec shown in Figure 9a. Both methods show similar overall trends, and a direct comparison is made difficult, because of the larger spectral line widths that are observed in the dynamic calculations, with the spectrum showing similar trends in both sets of calculations; for instance, Form III shows very little absorption below 110 cm^–1^ using either method, while Form II shows the lowest absorption and highest intensity mode in both. The integrated absorptions are different between the two calculations with the integrated absorption of the static calculations being at least half that of the dynamic calculations for all forms.

Finally, Figure 9e shows the results for the dynamic calculation of the disordered supercell DCI32a of Form I at 300 K. As with the static calculations shown in Figure 9d, there are broad spectral features roughly centered at ∼50, 70, and 100 cm^–1^ with fewer modes resolved than in the ordered systems. These modes are shifted to slightly lower frequencies, which is probably owing to the introduction of anharmonicity compared with the static calculations, but the shifts are very small. Finally, as with the dynamics calculations in Figure 9d, the integrated intensities are now much higher, in this case by a factor of nearly 4, compared to that in Figure 9b.

#### 3.3.2 Calculated Infrared Spectra and the S–H Stretching Region

**Figure 11:**
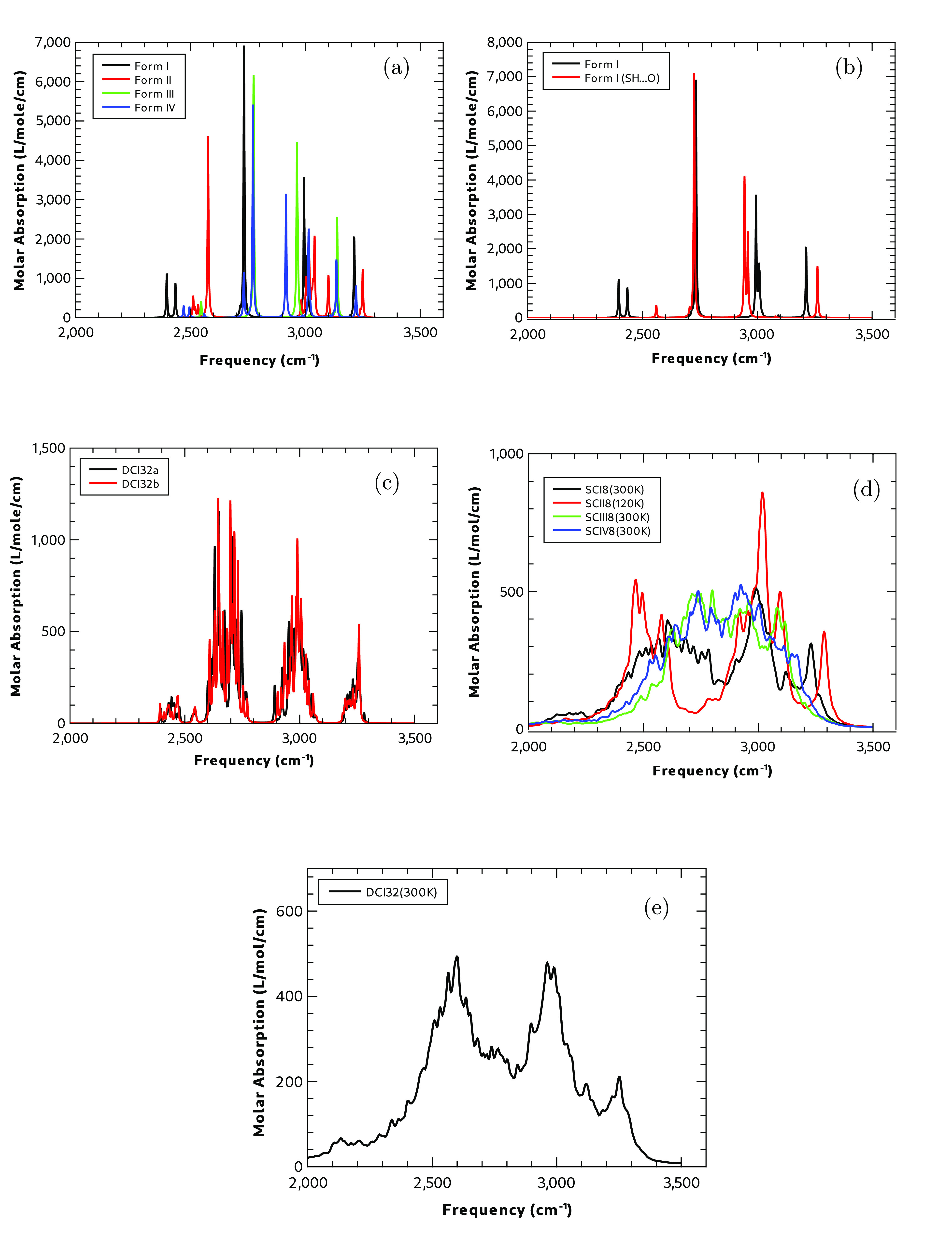
Calculated IR spectra above 2000 cm^–1^ (a) using static calculations of all four polymorphs (b) using static calculations for Form I SH**···**S and SH**···**O hydrogen-bonding motifs (c) using static calculations of the Form I dispersed disorder models containing 32 molecules (d) using the dynamic calculations of the four polymorphs and (e) using dynamic calculations of the disorderd supercell (DCI32a) of Form I.

Figure 11 summarizes the calculated spectra from the five static and dynamic calculation procedures in the S–H stretch region which occurs in the region between 2400 and 2600 cm^–1^ and the usually more intense absorption region above this associated with the N–H stretch, which can also prove informative. While, experimentally, the peaks in this region will be very broad, because of the homogeneous broadening caused by the strong hydrogen bonding in all the polymorphs, the differences seen in the calculated spectra can prove insightful in understanding the energetics involved within the different polymorphs along with the disorder seen within these systems.

In all the static calculations, the signature of the S–H stretch appears to be a doublet of absorptions separated by ∼50 cm^–1^. Figure 11a shows the spectra from the static calculations for each of the four polymorphs. Form I has the lowest frequency absorption for the S–H stretch, followed by Forms IV, II, and III, in that order. Form III is the only polymorph that does not exhibit the doublet splitting of this vibration, and it is also the polymorph with the highest-frequency S–H stretching vibration. Form II shows strong absorption at 2560 cm^–1^, because of an N–H stretching vibration, which is part of the N–H**···**O hydrogen-bonded network in this polymorph. In the high-frequency region associated with N–H and S–H stretching, only Form I shows any significant absorption associated with the S–H stretching motion at ∼2400 cm^–1^. Experimentally,^40^ the S–H stretching frequency in Form I is 2551 cm^–1^ and the N–H stretching frequency is 3064 and 3166 cm^–1^.

Figure 11b shows the calculated high-frequency infrared spectra for the two unit cells of Form I used to represent domain disorder with substantial differences seen between the two spectra. The SH**···**O hydrogen bonding motif shows a shift to lower frequency relative to the SH**···**S bonding motif at ∼3000 cm^–1^ and to higher frequency for the absorption at ∼3200 cm^–1^ associated with an NH**···**O hydrogen bond. For the SH**···**S bonding pattern, there is a doublet pattern of absorption associated with the S–H bond between 2430 and 2460 cm^–1^. While for the SH**···**O pattern, there is a single, weaker peak at 2560 cm^–1^ for the SH**···**O hydrogen-bonded pattern. Section S7 in the SI compares the calculated spectra of the two hydrogen-bonding motifs over the full frequency range.

Figure 11c shows the static calculated spectra for the disordered supercells DCI32a and DCI32b. Much like the terahertz spectral region, there are spectral similarities to both the SH**···**S and SH**···**O unit cells shown in Figure 11b. Again, as with the terahertz spectra, this is not a simple addition of spectral features but rather a new spectrum showing some similarity associated with the differing hydrogen bonding environments. Figure 11d shows the dynamic calculated spectra for each of the four polymorphs. It is instructive to compare these calculations with the equivalent static calculations using PDielec shown in Figure 11a. In both, Form I shows the lowest frequency absorption while Form II shows the highest, a direct comparison is made particularly difficult here, because of the very large spectral line widths that are observed in the dynamic calculations, however, unlike in the terahertz spectral region, the overall integrated intensities and spectral shape are similar in both plots. Finally, Figure 11e shows the dynamic calculated spectrum for the disordered supercell of Form I, DCI32a. The inclusion of disorder increases the spectral widths, compared to the dynamic calculation without disorder, but both show three peaks centered at ∼2500, 3000, and 3250 cm^–1^. One thing of special interest in this spectral region is that, although its difficult to make a direct comparison in intensities between the static and dynamic calculations, because of the differing widths, the integrated spectral intensities are similar in both types of calculations, unlike the terahertz spectral region, where large differences were observed.

##### 3.3.2.1 Temperature Dependence of Ordered Form I

**Figure 12:**
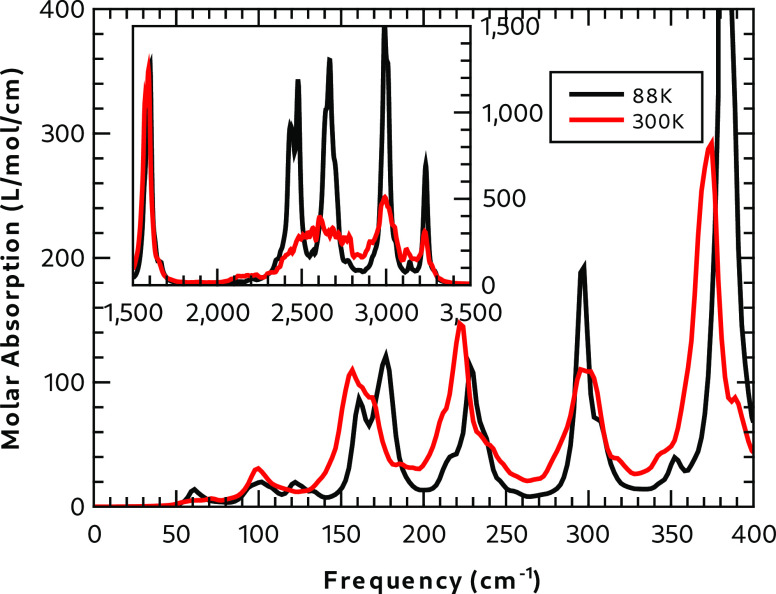
Effect of the temperature on the spectra of Form I from the molecular dynamics calculations of SCI8.

One advantage of the dynamic calculations is that any effects of the temperature can be explicitly included in the calculation. Figure 12 compares the absorption spectra of Form I calculated at 88 and 300 K using supercell SCI8. The figure shows that, in the low-frequency regime, the high-temperature absorption peaks are shifted to lower frequency, because of the larger cell size. This effect is noticeable up to ∼1700 cm^–1^. Apart from this systematic effect, the spectra up to 1700 cm^–1^ are very similar but with a broadening of the high-temperature absorption peaks. The window function used for the convolution of the dipole moment correlation function was chosen to provide narrow absorption peaks in the low-frequency end of the spectrum (see Section S5.4 in the SI). Above 1700 cm^–1^, there is a significant change in the shape of the absorption peaks. The low-temperature simulation shows four distinct peaks: a doublet peak at 2429 and 2479 cm^–1^, arising from the S–H stretch, and peaks at 2667, 2997, and 3233 cm^–1^, all of which are associated with N–H stretching of N–H**···**O hydrogen-bonded moieties. At 300 K, the absorption peaks associated with the S–H and the lowest-frequency N–H stretches broaden, so there is one broad absorption band with two distinct absorption peaks at higher frequencies.

##### 3.3.2.2 Temperature Dependence of Dispersed Disorder in Form I

**Figure 13:**
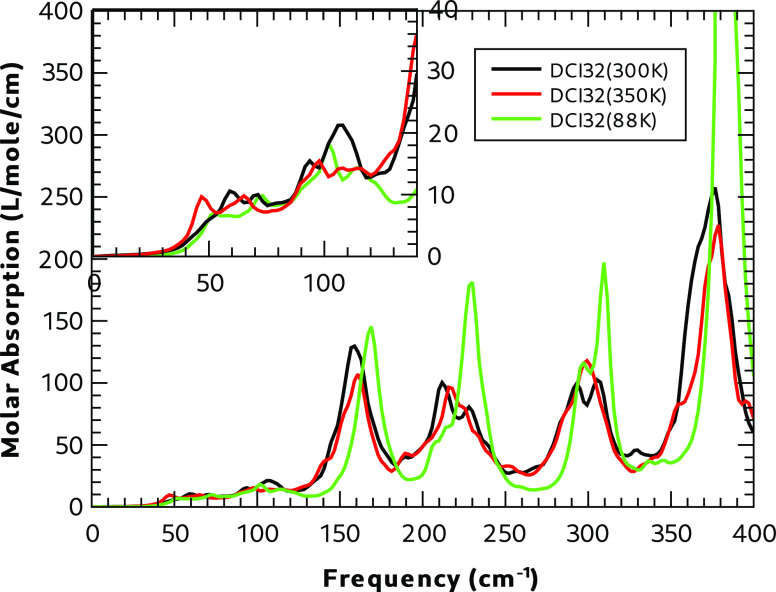
MD IR absorption in the low-frequency region of DCI32.

**Figure 14:**
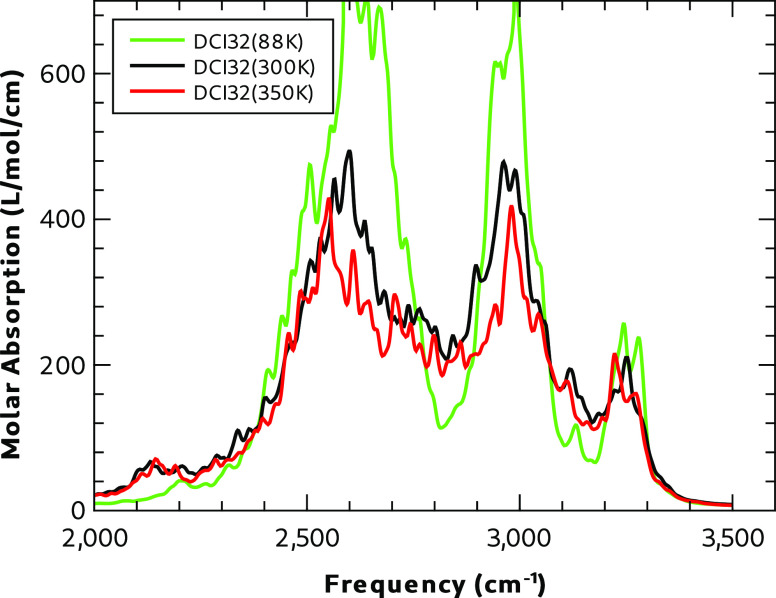
MD IR absorption in the high-frequency region of DCI32.

The MD simulations of supercell DCI32 at 88, 300, and 350 K were used to calculate the absorption spectrum of the dispersed disorder in Form I. The results from simulations at the two higher temperatures lead to very similar absorption profiles (see Figures 13 and 14). In the frequency regime between 100 and 400 cm^–1^, the low-temperature spectrum has sharper absorptions, which are shifted to a higher wavenumber, consistent with a lattice vibration in a smaller volume. The predicted low-frequency spectrum (Figure 13) is calculated from the cell dipole moment by using a window function to give narrower peaks and greater resolution than that used for the frequencies above 400 cm^–1^. The high-frequency region shows weak absorption above 2000 cm^–1^, presumably from the S–H stretch vibration. Above this frequency are more intense and broad transitions with three significant peaks associated with the N–H stretching vibration.

## 4 Comparison with Experiment

**Figure 15:**
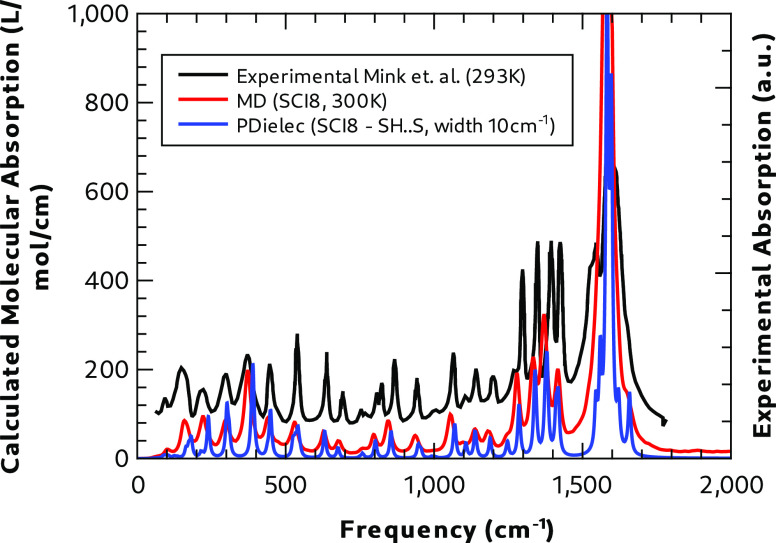
Comparison of Form I calculated and experimental^15^ infrared absorption spectra at 300 K, below 2000 cm^–1^.

The far-infrared spectrum measured at 293 K by Mink et al.^15^ is compared with the spectra calculated for Form I without disorder by static DFT (SH**···**S bonding pattern) and MD (SCI8 supercell at 300 K) in Figure 15. The experimental spectrum was obtained from the paper by digitizing the relevant plot. All peaks in the experimental spectrum correlate well with both sets of calculations, neither of which take account of the S–H**···**O hydrogen bonding motif. Equally good agreement between the calculated and experimental spectra is also seen when the calculations include such hydrogen bonding, which indicates that this region of the spectrum does not discriminate well between the hydrogen bond motifs seen at high temperature in Form I. The intense peak above 1500 cm^–1^ is owing to deformation of the ammonium ion, while the four peaks below 1500 cm^–1^ are mainly methylene group deformations. Close comparison of both calculated spectra and experiment shows an improved correlation between the experimental results and the dynamical calculation rather than the static calculation. This is largely because, in the dynamic calculation, no assumption has been made about harmonicity or spectral peak shape, which leads to a general shift to lower frequencies, compared to the static calculation, particularly for modes below 500 cm^–1^. The increased integrated absorption observed for dynamic calculations at lower frequencies also seems to correlate well with the relative absorption seen in the experiment, particularly for the two larger peaks below 300 cm^–1^.

**Figure 16:**
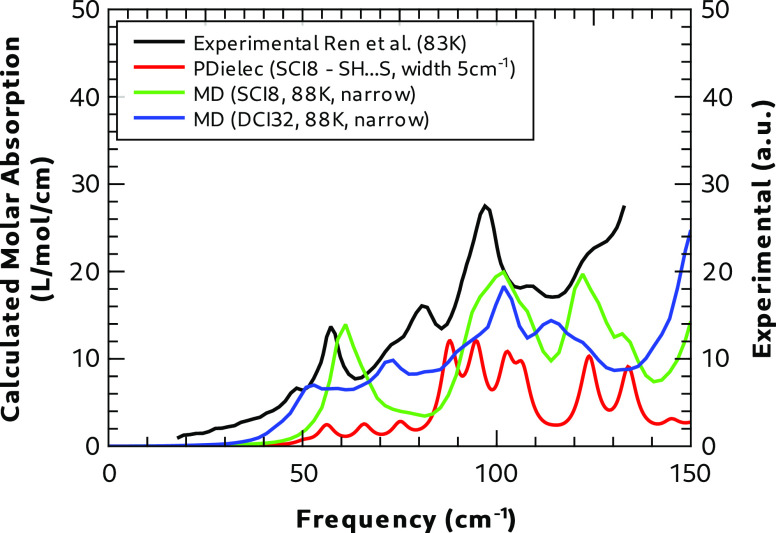
Comparison of Form I calculated and experimental^16^ terahertz absorption spectra at 83 K.

As mentioned previously, the terahertz region should be more sensitive to the type of intermolecular bonding found in the crystal. Experimental results are available in this region from the work of Korter et al.^12^ and Ren et al.^16^ Here, we compare our calculations with spectra digitized from the publications of Ren et al.^16^Figure 16 compares the calculated spectra of Form I with and without disorder from MD at 88 K and static DFT calculations of the SH**···**S bonding pattern with the experimental spectrum at 83 K. Above 50 cm^–1^, the static calculation predicts four weak absorption peaks of similar intensity, which, in the MD calculations, have overlapped into a single broad peak with significantly higher integrated intensity. The window used for the dipole correlation function in this case was chosen to produce narrower peak widths. The MD of the SCI8 structure without disorder predicts broad absorption peaks at 100 and 122 cm^–1^, which correlate well with the experiment. While the peak at 122 cm^–1^ is present in the static calculation, peaks at ∼100 cm^–1^ show significantly more structure and less broadening than is seen experimentally or in the dynamic calculation of the nondisordered calculation. Experimentally, there is a peak at 81 cm^–1^ and a broad shoulder at 49 cm^–1^ that do not correlate with either of these calculated spectra.

The dynamic spectrum calculated using the disordered supercell (DCI32) correlates best with experiment. While there are some differences between the two MD calculations above 100 cm^–1^, which it could be argued improve correlation with the experiment, it is the large differences below 100 cm^–1^ that seem to be key. In particular, there is now a broad feature in the calculated spectrum that stretches from 40 cm^–1^ upward that seems to correlate with the experiment, at least in terms of peak position, if not in terms of relative intensity of the many modes involved. In particular, this is by far the closest match to the peaks in the experiment at 81 and 49 cm^–1^ that is not seen in the other calculations. From this comparison, it seems clear that both dynamics and disorder have an influence on the measured terahertz spectrum of Form I.

**Figure 17:**
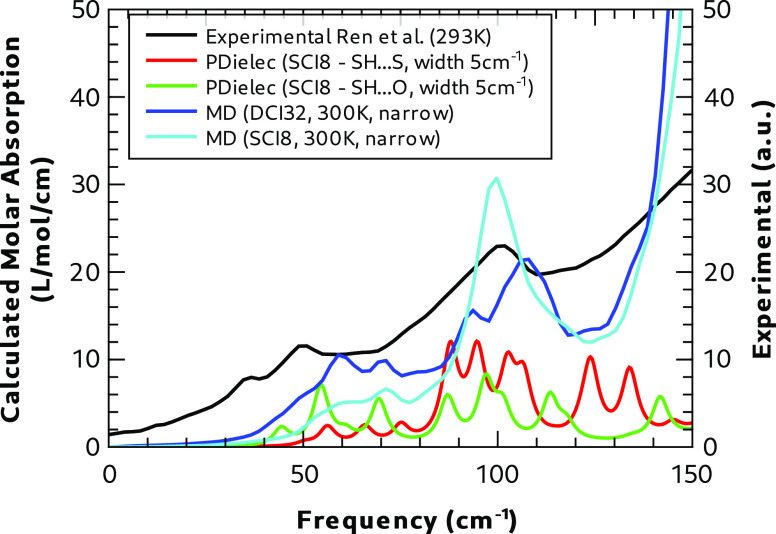
Comparison of Form I calculated and experimental^16^ terahertz absorption spectra at 293 K.

The high-temperature experimental spectrum taken at 293 K is shown in Figure 17, where it is compared with several different calculations. The experimental spectrum is broader than the low-temperature one, and the peak maximum has moved to lower frequencies, as would be expected. In the plot, we show calculations using static calculations of both hydrogen bonding motifs in Form I along with dynamic calculations including a SH**···**S-only motif (SCI8) and the larger cell containing disorder with a mixture of SH**···**S and SH**···**O motifs (DCI32), both at 300 K.

Static calculations of either hydrogen bonding motif show little correlation with the experiment, in terms of either peak position or relative peak intensities. Both dynamic calculations show much better overall correlation with the experiment, with both calculations and the experiment showing a broad feature below 80 cm^–1^ consisting of several modes, and a second broad feature centered at ∼100 cm^–1^. In the disordered system the mode centered at ∼100 cm^–1^ splits into two clear peaks with a width and relative intensity that much more closely resembles the experiment. This splitting of modes in the disordered case might also explain the experimental peak at 81 cm^–1^ in the 83 K experimental spectrum, which was not well-reproduced by the dynamic calculation at 88 K that did not include disorder. Both dynamic calculations predict a similar feature below 80 cm^–1^, although the relative intensities of the individual peaks vary between the calculations; both calculations overpredict the frequency of these modes, compared to the experiment, by 20 cm^–1^. The results of Korter et al.^12^ do not shed any more light on the lowest frequency peak as the signal at this very low frequency is quite noisy.

It is clear that the dynamics included in the MD calculations are important in interpreting the measured spectral parameters at these low frequencies, in terms of both peak position and relative peak intensity. The differences upon the inclusion of disorder into the calculation are subtle, but it should be noted that the changes between the experimental spectra recorded at 293 and 83 K are small, particularly given the likely change in intermolecular hydrogen bonding that has been shown through crystallography. This notwithstanding, the splitting of the peak centered at ∼100 cm^–1^ in the disordered calculation, coupled to the changes in relative intensity of peaks that this leads too, suggests that the terahertz spectrum of Form I is sensitive to the disorder known to exist in Form I.

However, note that, bearing in mind the sensitivity of the heat capacity to thermal history,^6,7^ it is possible that the experimental measurements at low temperatures have been performed on samples that have not been able to equilibrate, while previous Raman measurements proposed the influence of disordered water^9^ on the spectrum that was not controlled in the experimental infrared and terahertz measurements or included in the calculations presented here.

## 5 Conclusions

Static energy calculations of the rotation of the C–C–S–H angle showed that all four polymorphs are likely to show disorder in the sulfhydryl hydrogen bonding pattern at higher temperatures. This was confirmed, at least in the case of Form IV, by MD calculations, which showed a change in the hydrogen bonding pattern during the simulation. This result is also consistent with the experimental observation of transitions in the C–C–S–H torsion and C–C–S angle bands of the dynamical susceptibility of the hydrogen atoms in Form II between 150 and 200 K.^17^

The static free-energy calculations showed that Forms III and IV become more stable than Form I or II as the pressure increases. This agrees with the experimental stability of these polymorphs only at higher pressures. However, the pressure at which this crossover was calculated to occur (6 GPa) is considerably greater than the pressures required experimentally (2.6 and 1.7 GPa for Forms III and IV, respectively). This may be owing to the inclusion of only harmonic contributions to the free energy when it is clear from the IR spectral calculations presented here that there is a high degree of anharmonicity in these systems, particularly at low frequencies, that will significantly affect the vibrational contribution to the free energies in these systems.

The MD calculations of the dispersed disordered model for Form I showed several changes in the hydrogen bonding motif during the simulation, and this supports the case for this model over the domain disordered one. Attempts to further support this claim by comparing the calculated IR spectra from these models to experiments are frustrating. The predictions for both models are very similar above 150 cm^–1^ and all compare well with the experiment, suggesting that the mid-IR region appears to be insensitive to the disorder and even the S–H stretch region is difficult to interpret as the broad experimental peak widths mask the effect of the different hydrogen bonding environments.

In the terahertz region, the agreement between experiment and static calculations is poor, and the importance of the dynamics of the system to the resultant spectrum is clear. Below 100 K, anharmonic effects and disorder are expected to be small and the agreement between the calculated spectrum (88 K, based on SCI8) and the experimental spectrum of Ren et al.^16^ is quite good, but there are some features missing. The inclusion of the disorder into the system by using the DCI32 cell, in conjunction with the added benefits of the dynamics, improves the correlation between the calculation and experiment. This is surprising considering that crystallography would suggest that there is much less disorder at these temperatures. The correlation could be improved further below 100 cm^–1^, and this could be owing to the amount of disorder at these temperatures with the DCI32, assuming an even mix of both hydrogen-bonding motifs. For instance, the peak at 50 cm^–1^ in the experimental spectrum could be owing to some S–H**···**O bonding that has become frozen in on preparation of the sample as static calculations indicate that only this hydrogen bonding motif gives rise to such a low-frequency absorption. Alternatively, it could be owing to the boson peak that has been identified in the low-temperature Raman spectrum at 40 K,^9^ which was associated with disorder of the small amount of water in the sample. Comparisons between experiment and theory are further complicated by light scattering from air voids in the sample, which is known to give rise to a background rise in absorption and has not been taken into account here.^21^

A compelling reason for performing MD calculations for the calculation of IR spectra is to capture the anharmonic effects that can be expected. Our results here show that the MD calculations are very useful below ∼300 cm^–1^, where they capture features that static calculations do not. Above this threshold, the agreement between MD and static is very good and probably does not justify the computational expense of the MD calculation. Below this threshold and especially below 150 cm^–1^ there are significant differences in the comparisons between the methods, in particular, the static methods predict lower absorption than that predicted by MD. The reason for this is not clear, since, at higher frequencies, the absorption is very similar using either method. One possibility could be that the dipole moment derivatives found through MD are enhanced by being in an anharmonic region. A similar idea was presented by Bordallo et al.,^17^ who suggested that, once the rotational motions became free, there would be a strong coupling with the phonons, giving rise to much larger amplitude motion. But the simulation of Form I at 88 K using MD still showed an enhanced absorption at low frequencies, and the motion at such a low temperature is expected to be harmonic, where the methyl rotors are not free to rotate.

Another important point, in terms of the dynamic calculations, is the impact of numerical processing of the dipole moment variation on the quality of the predicted spectra from MD calculations. Because of the relatively short simulation times and the size of the simulation cell, it is common practice to apply a window filter to the dipole moment correlation function. This window ensures that the dipole moment decays as it would in a real system, with the decay constant linked to the lifetime of the phonons and the width of peaks. The fact that this is imposed on the system means that peak widths are not a true reflection of the phonon lifetimes. Having said that, the comparison of the 88 and 300 K MD simulations of Form I did show some vibrational broadening that is not just a function of the windowing function and provides calculated spectra with widths comparable to those observed experimentally.

While this study has focused on l-cysteine and its polymorphs, it is likely that disorder in any hydrogen-bonded materials, or, even more generally, flexibility that allows a large range of dihedral angles to be explored, will influence the terahertz spectral properties of the material. In the short term, it is important to investigate the disagreement between experimental and calculated spectra to explore how general an effect this is, while longer term, it will be interesting to investigate if THz spectral measurements can be used to rapidly quantify disorder in a crystalline material.

Computational methods are continually improving and recent developments in machine learning for force fields^41^ will allow molecular dynamics simulations of the type reported here to to be extended in time and space, allowing simulation to capture more of the essential physics, which is required for these systems.

## Supporting Information Available

A summary of the experimental crystal structures; complete protocols for calculations using VASP, Phonopy and CP2K; the development of the theory for calculating the permittivity from an MD simulation; an overview of the ordered and disordered supercells used in the calculations; a summary of the optimization and free energy calculations using VASP; an overview of the static calculations of the IR and THz spectra; a summary of the MD simulations of the various supercells; an overview of the dynamic calculations of the IR and THz spectra including a comparison between spectra calculated using the molecular dipole moment fluctuations included within the Travis code and the total cell dipole fluctuations described in this article (PDF)
