# Supplementary material for: Exploring the Stability and Disorder in the Polymorphs of L-Cysteine through Density Functional Theory and Vibrational Spectroscopy
Source: Cryst Growth Des. 2023 Jun 29;23(8):5734–47. doi: 10.1021/acs.cgd.3c00375 (PMC10401577; doi:10.1021/acs.cgd.3c00375)
Supplement: Supplementary file 1 — cg3c00375_si_001.pdf [file cg3c00375_si_001.pdf]

Exploring the Stability and Disorder in the Polymorphs of L-Cysteine through Density Functional Theory and Vibrational Spectroscopy - Supplementary Information

John Kendrick* and Andrew David Burnett

University of Leeds, Leeds, LS2 9JT, UK

E-mail: j.kendrick@leeds.ac.uk

Contents

1 Experimental Crystal Structures **5**

2 VASP Protocol **7**

2.1 VASP Energy Cutoff 7

2.2 VASP Dispersion Correction Methods for Form I 8

3 Phonopy Protocol **9**

4 CP2K Protocol **9**

4.1 CP2K Energy Cutoffs 9

5 Calculation of Dielectric Permittivity from MD Calculations **11**

5.1 Static Permittivity 11

5.2 Frequency Dependent Permittivity 13

5.3 The Total Permittivity 13

5.4 The Discrete Dipole Moment Correlation Function 14

5.5 Calculation of the Absorption Spectrum 15

6 Super-cells used in VASP and CP2K calculations **16**

7 VASP Results **17**

7.1 Optimisation of all Polymorphs at Experimental Pressures 17

7.2 Optimisation of all Polymorphs at Zero Pressure 17

7.3 Variation of Polymorph Geometry with Pressure 18

7.4 VASP Study of SH...S and SH..O Hydrogen Bonding in Form I 24

7.5 Behaviour of Torsion Angles 24

7.6 Form I Unit-Cells and Super-Cells 25

7.7 Calculated Infrared and THz Frequencies for SH...S and SH...O Hydrogen-bonding Motifs . 29

7.7.1 Frequencies 29

7.7.2 IR Absorption 31

7.8 Super-cell Infrared Spectra 34

7.8.1 Comparison of DCI8a and DCI8b 34

7.8.2 Comparison of DCI16 and DCI16b 36
7.8.3 Comparison of DCI32a and DCI32b 38

8 CP2K Results **40**

8.1 Molecular Dynamics Calculations of Super-cells 40
8.2 NVT Calculations of Super-cells 44
8.3 IR Absorption from Molecular Dipole Fluctuations 48
8.4 IR Absorption from Cell Dipole Fluctuations 52

List of Tables

1 Experimental Unit Cell Information 6
2 Optimised unit-cell parameters - no dispersion correction 7
3 Frequencies in cm^{-1} of the lowest 19 vibrational modes calculated with no dispersion correction 8
4 Effect of dispersion correction on Form I Optimised unit-cell parameters 9
5 CP2K behaviour of energy with cutoff 10
6 CP2K behaviour of energy with relative cutoff 10
7 Electronic components of the permittivity at zero frequency 14
8 Window parameters used in the calculation of correlation functions 15
9 Super-cells used in the VASP and CP2K calculations 16
10 VASP optimised unit-cell parameters for polymorphs at experimental pressures 17
11 VASP optimised unit-cell parameters for polymorphs at experimental pressures - percentage deviations from experiment 17
12 VASP optimised unit-cell parameters for polymorphs at zero pressure 18
13 VASP optimised unit-cell parameters for polymorphs at zero pressure - percentage deviations from experiment 18
14 VASP internal energy as a function of pressure 21
15 VASP volume as a function of pressure 22
16 Vibrational Helmholtz free energy at 300K 22
17 Total Helmholtz free energy at 300K 22
18 Energies and optimised unit-cell parameters for different H-bonding patterns 24
19 Torsion angles from experiment and from VASP optimisations 25

20 Average super-cell parameters calculated by CP2K 40

1 Experimental Crystal Structures

A summary of experimental determination of the four polymorphs of L-Cysteine is shown in Table 1. The results include the change in Form I as a function of temperature[1] and study of the effect of pressure and the stability of all the polymorphs.[2] The Table also provides the Cambridge Structural Database (CSD) codes.[3] Not all structures had an unambiguous assignment of the positions of the hydrogen atoms bonded to sulfur. In the case of Form IV, no such hydrogen positions were determined. Where calculations on Form IV have been performed hydrogens have been placed 1.4 Å from the sulfur atom pointing to the nearest hydrogen bond acceptor (either another sulfur or an oxygen atom) before geometry optimisation was performed. In the case of room temperature Form I structures, the structures corresponding to the S-H...S hydrogen and the S-H...O bonding patterns were chosen for optimisation. The S-H...O bonding pattern was created by rotating the C-C-S-H torsion angle to move the hydrogen into position where it could bond favourable with the oxygen acceptor atom.

Table 1: Experimental Unit Cell Information

Form	CSD code	P(GPa)	T(K)	a(Å)	b(Å)	c(Å)	$\beta/^\circ$	Vol(Å^3)	Symmetry	τ_1*	τ_2*	H-bond
I	LCYSTN12/21[4]	0.0	293	8.116	12.185	5.426	90.0	536.55	$P2_12_12_1$			unknown
I	LCYSTN22[5]	0.0	30	8.144	11.937	5.416	90.0	526.44	$P2_12_12_1$	87.0		SH...S
I	LCYSTN23[2]	0.0	293	8.111	12.162	5.421	90.0	534.76	$P2_12_12_1$	-67.3	74.2	disorder
I	LCYSTN27[2]	1.8	293	7.415	12.000	5.332	90.0	474.40	$P2_12_12_1$			unknown
I	LCYSTN28[1]	0.0	105	5.420	8.150	12.011	90.0	530.57	$P2_12_12_1$	-69.0	86.9	disorder
I	LCYSTN29[1]	0.0	125	5.421	8.136	12.037	90.0	530.92	$P2_12_12_1$	-77.6	86.6	disorder
I	LCYSTN30[1]	0.0	150	5.418	8.127	12.068	90.0	531.36	$P2_12_12_1$	-78.1	85.0	disorder
I	LCYSTN31[1]	0.0	175	5.419	8.120	12.095	90.0	532.21	$P2_12_12_1$	-79.8	83.5	disorder
I	LCYSTN32[1]	0.0	200	5.420	8.116	12.117	90.0	533.05	$P2_12_12_1$	-75.3	84.9	disorder
I	LCYSTN33[1]	0.0	225	5.422	8.112	12.141	90.0	533.96	$P2_12_12_1$	-79.3	83.6	disorder
I	LCYSTN34[1]	0.0	250	5.423	8.112	12.153	90.0	534.65	$P2_12_12_1$	-77.2	86.5	disorder
I	LCYSTN35[1]	0.0	275	5.425	8.114	12.171	90.0	535.71	$P2_12_12_1$	-80.4	85.3	disorder
I	LCYSTN36[1]	0.0	295	5.423	8.122	12.180	90.0	536.48	$P2_12_12_1$	-78.4	87.0	disorder
II	LCYSTN04[6]	0.0	120	9.441	5.222	11.337	109.0	528.47	$P2_1$	-64.6	80.8	both
III	LCYSTN24[2]	2.6	293	8.056	10.488	5.347	90.0	451.79	$P2_12_12_1$	-72.4		SH...O
III	LCYSTN25[2]	4.2	293	7.998	10.298	5.284	90.0	435.20	$P2_12_12_1$	-66.1		SH...O
IV	LCYSTN26[2]	1.7	293	8.105	5.442	10.916	94.9	479.72	$P2_1$			unknown

* τ_1 and τ_2 refer to the C-C-S-H dihedral angle of each of the symmetry unique molecules in the unit-cell

2 VASP Protocol

2.1 VASP Energy Cutoff

Density Functional Theory (DFT) calculations were performed with the VASP package[7] using the Perdew-Burke-Ernzerhof (PBE) functional[8] and the Projector Augmented Wave (PAW) pseudo-potentials[9] distributed with VASP 5.4.1. The selection of plane wave cutoff was based on a series of calculations of the optimised unit cell of Form I and the frequencies of the gamma point phonon modes at 600, 700 and 800 eV which are reported in Table 2 and Table 3. No dispersion correction was used for these calculations. Based on these results all further DFT calculations use a plane wave cutoff of 600 eV and a reciprocal space k-point resolution of 0.2 $Å^{-1}$

Table 2: Optimised unit-cell parameters - no dispersion correction

Cutoff (eV)	600	700	800
a (Å)	8.2991	8.2984	8.3005
b (Å)	12.1541	12.1534	12.1577
c (Å)	5.4989	5.4980	5.4993
Volume ($Å^3$)	554.66	554.50	554.96

Table 3: Frequencies in cm^{-1} of the lowest 19 vibrational modes calculated with no dispersion correction

Mode	600 eV	700 eV	800 eV
1	−2.3	−1.4	−3.5
2	−2.3	−1.0	−2.2
3	−1.3	−0.7	−1.2
4	41.4	43.0	42.7
5	43.6	44.9	44.5
6	43.8	48.6	46.1
7	50.0	52.1	48.6
8	61.2	60.4	62.0
9	61.9	64.4	65.4
10	69.4	69.8	70.5
11	74.1	75.0	75.1
12	80.7	83.7	84.6
13	83.1	86.9	86.2
14	85.2	87.0	86.2
15	88.8	92.1	92.2
16	89.8	94.3	92.7
17	90.3	94.3	94.1
18	92.5	95.9	96.1
19	96.3	99.8	97.3

2.2 VASP Dispersion Correction Methods for Form I

The effect of different dispersion methods was explored using optimisation of the Form I unit-cell. The methods evaluated included the Grimme DFT-D3 method[10] with Becke-Johnson[11] damping (GD3/BJ) with no additional Axilrod-Teller-Muto (C9) correction, the charge dependent Tkatchenko-Scheffler method[12] with (TSi) and without iterative Hirshfeld partitioning (TS), the dDsC dispersion correction method[13] and the Many Body Dispersion (MBD) method.[14]

The wavefunction was converged so that the energy changed less than 1×10^{-8} eV and the atom positions and unit cell dimensions were optimised so that all forces were less than 5×10^{-4} eV/Å.

The results of calculations of the optimised unit-cell dimensions of Form I and their percentage deviation from experiment are reported in Table 4. The method that gives the closest agreement with experiment is MBD, with the lowest average deviation in unit cell parameters. However, it was found that MBD in this version of VASP did not scale well with system size and proved difficult to apply to the large unit cells which were used later in the project. It was therefore decided to use the GD3/BJ dipersion correction as this performed nearly as well as MBD and had no problems in treating the larger unit-cells. The GD3/BJ

dispersion has been shown to be reliable in previous work.[15]

For full geometry optimisations of the unit-cell and the molecular geometries, the default optimiser within VASP were used. For those calculations which required constraints the GADGET optimiser[16,17] was used, which has an interface to the VASP package. The optimisations were terminated when the following criteria were met; predicted internal coordinate changes were less than 0.02 (Bohr or radians), the gradients were less than 0.0004 (Hartree/Bohr or Hartree/radian) and the energy change was less than 1.0×10^{-5} Hartree.

Table 4: Effect of dispersion correction on Form I Optimised unit-cell parameters [*]

Method	a(Å)	b(Å)	c(Å)	Vol(Å^3)	δa(%)	δb(%)	δc(%)	δV(%)
None	8.2991	12.1541	5.4989	554.66	1.9	1.8	1.5	5.4
GD3/BJ	8.1564	11.8717	5.4152	524.36	0.2	−0.5	0.0	−0.4
TS	8.2014	11.8633	5.4246	527.79	0.7	−0.6	0.2	0.3
Tsi	8.2056	11.8529	5.4409	529.18	0.8	−0.7	0.5	0.5
dDsC	8.1247	11.8493	5.3874	518.65	−0.2	−0.7	−0.5	−1.5
MBD	8.1692	11.8837	5.4201	526.19	0.3	−0.4	0.1	−0.1
Experimental	8.1435	11.9365	5.4158	526.44				

[*] Experimental values are taken from X-ray crystallography results at 30K[5]

3 Phonopy Protocol

Phonopy[18] was used to calculate the quasi harmonic free energies of each polymorph as a function of pressure and temperature. The super-cells used to calculate the atomic displacements for the calculation were 1x1x2, 1x2x1, 1x1x2 and 1x2x1 for Forms I, II, III and IV respectively. Single point calculations were performed with VASP at these displacements using the same settings a described in Section 2.

4 CP2K Protocol

4.1 CP2K Energy Cutoffs

Ab Initio molecular dynamics calculations were performed with CP2K package.[19] As was used in VASP, all calculations employed the PBE exchange-correlation potential along with the GD3/BJ dispersion correction with no additional Axilrod-Teller-Muto (C9) correction to allow a fair comparison with VASP. The Molopt double zeta valence plus polarisation basis sets[20] were used with the GTH PBE pseudopotentials.[21]

CP2K has two energy cutoffs which affect the accuracy of the calculation; the cutoff and the relative cutoff.

With the relative cutoff set at 60 Rydberg, the cutoff was varied and the effect on the first cycle of the self consistent procedure was calculated. The results are show in Table 5. Based on these results a cutoff of 450 Rydberg was chosen. With this value of the cutoff, the relative cutoff was changed and the results shown in Table 6. The final values of the cutoff and the relative cutoff used in the subsequent calculations were 450 and 50 Rydberg respectively.

Table 5: CP2K behaviour of energy with cutoff

Cutoff (Rydberg)	Energy (Hartree)
50	−2225.452 48
100	−2239.309 61
150	−2239.457 98
200	−2239.310 33
250	−2239.281 23
300	−2239.267 15
350	−2239.257 72
400	−2239.253 19
450	−2239.252 71
500	−2239.252 66

Table 6: CP2K behaviour of energy with relative cutoff

Cutoff (Rydberg)	Energy (Hartree)
10	−2241.374 86
20	−2239.347 85
30	−2239.257 72
40	−2239.252 76
50	−2239.252 73
60	−2239.252 71
70	−2239.252 71
80	−2239.252 71
90	−2239.252 71
100	−2239.252 71

Molecular dynamics calculations were performed using both the NPT and NVT ensembles. The canonical sampling through velocity rescaling (CSVR) thermostat was used to constrain the temperature with a time constant of 100 fs and the pressure was constrained with a Nose-Hoover-Chain with the same time constant. The time step for the simulations was 0.5 fs.

After equilibration of the NPT calculations the average cell dimensions were determined from a simulation of at least 3 ps. The average cell dimensions from the NPT calculations were then used in the NVT

ensemble simulations. NVT equilibration was at least 2 ps before a production run of at least 30 ps.

5 Calculation of Dielectric Permittivity from MD Calculations

The development of the theory for calculating the permittivity from an MD simulation closely follows the appendix in the paper by Chen and Li.[22] SI units are used here and account is taken of the fact that the dipole moment of the cell is not zero, but it may have a permanent dipole moment.

5.1 Static Permittivity

The displacement field is related to the polarisation of the system due to the applied field.

$$\mathbf{D} = \mathbf{P} + \epsilon_0 \mathbf{E} = \epsilon \mathbf{E} \tag{1}$$

Here ϵ_0 is the permittivity of free space and $\mathbf{P}$ is the induced polarisation due to an electric field. The permittivity, ϵ, is given by the relationship between the displacement field and the applied field $\mathbf{E}$; The induced polarisation and the permittivity are also related to the susceptibility χ

$$\mathbf{P} = \epsilon_0 \chi \mathbf{E} \tag{2}$$

$$\epsilon = \epsilon_0 (\mathbf{1} + \chi) \tag{3}$$

or defining the relative permittivity (dielectric constant), $\epsilon_{\mathbf{r}}$;

$$\epsilon_{\mathbf{r}} = \frac{\epsilon}{\epsilon_0} = \mathbf{1} + \chi \tag{4}$$

For a molecular dynamics calculation of an infinite system, we have a periodic cell with a fluctuating dipole moment $\mathbf{M}$. The average of the dipole moment vector can be written as;

$$\mathbf{M_{av}} = \langle \mathbf{M} \rangle \tag{5}$$

The average of the square of the dipole moment can be written as;

$$\langle \mathbf{MM} \rangle = \begin{bmatrix} \langle M_x M_x \rangle & \langle M_x M_y \rangle & \langle M_x M_z \rangle \\ \langle M_y M_x \rangle & \langle M_y M_y \rangle & \langle M_z M_z \rangle \\ \langle M_z M_x \rangle & \langle M_z M_y \rangle & \langle M_z M_z \rangle \end{bmatrix} \tag{6}$$

where $\mathbf{M}$ is a vector of the 3 components of the dipole moment, and the average is over the trajectory.

The polarisation and the average of the induced dipole moment $\mathbf{M_I}$ are related by (V is the volume of the cell);

$$\mathbf{P} = \frac{\langle \mathbf{M_I} \rangle}{V} \tag{7}$$

$$\mathbf{M_I} = \mathbf{M} - \mathbf{M_{av}} \tag{8}$$

The expectation value of the dipole moment of a system perturbed by a field $\mathbf{E}$ according to Chen and Li (equation A6) is;

$$\langle \mathbf{M} \rangle_E = \langle \mathbf{M} \rangle + \beta \langle \mathbf{M}(\mathbf{M} - \langle \mathbf{M} \rangle) \rangle \cdot \mathbf{E} \tag{9}$$

Here $\beta = \frac{1}{kT}$. We are interested in the induced dipoles which are the terms only depending on the field. This gives;

$$\langle \mathbf{M_I} \rangle_E = \beta \langle \mathbf{M}(\mathbf{M} - \langle \mathbf{M} \rangle) \rangle \cdot \mathbf{E} \tag{10}$$

Since the polarisation is related to the field through the permittivity tensor (Equation 1);

$$\epsilon \cdot \mathbf{E} = \epsilon_0 \mathbf{E} + \mathbf{P} \tag{11}$$

$$\epsilon_\mathbf{r} \cdot \mathbf{E} = \mathbf{E} + \frac{\mathbf{P}}{\epsilon_0} \tag{12}$$

The induced polarisation can be expressed in terms of the average induced dipole moments, using equation 7.

$$\epsilon_\mathbf{r} \mathbf{E} = \mathbf{E} + \frac{\beta}{V \epsilon_0} \langle \mathbf{M_I} \rangle_E \tag{13}$$

Substituting the expectation value of the induced dipole moment (equation 9) and cancelling out the field, gives;

$$\epsilon_{\mathbf{r}} = \mathbf{1} + \frac{\beta}{V\epsilon_0}\langle \mathbf{M}(\mathbf{M} - \langle \mathbf{M} \rangle)\rangle \tag{14}$$

5.2 Frequency Dependent Permittivity

The starting equation for this is equation A13 in the paper by Chen and Li;

$$\epsilon_{\mathbf{r}}(\omega) = \frac{\beta}{\epsilon_0 V}\mathbf{L} + \mathbf{1} \tag{15}$$

$$L_{ij} = \langle M_i(0)M_j(0)\rangle F\phi_{ij} \tag{16}$$

$$F\phi_{ij} = \int_0^\infty e^{-i2\pi\omega t}\frac{dC_{ij}(t)}{dt}dt \tag{17}$$

$$F\phi_{ij} = 1 - i2\pi\omega \int_0^\infty e^{-i2\pi\omega t}C_{ij}(t)dt \tag{18}$$

$$C_{ij}(t) = \frac{\langle M_i(t)M_j(0)\rangle}{\langle M_i(0)M_j(0)\rangle} \tag{19}$$

Here $C_{ij}(t)$ is the dipole correlation function for dipole moments M_i and M_j and needs to be calculated from the trajectory.

5.3 The Total Permittivity

The sections above deal with the atomic motion contribution to the permittivity but the calculation of the absorption of spectrum in the infrared region requires the total permittivity and this requires the inclusion of the electronic polarisation at zero frequency ($\epsilon_{\mathbf{optical}}$). The assumption being that for insulators the electronic contribution is constant for the frequency range of interest in infrared spectroscopy. This term is calculated routinely in most DFT packages. In the calculations here the term has been taken from the VASP calculations of the phonon spectra.

$$\epsilon_{\mathbf{total}}(\omega) = \epsilon_{\mathbf{optical}}(0) + \epsilon_{\mathbf{r}}(\omega) \tag{20}$$

The zero frequency electronic components of the permittivity are summarised in Table 7.

Table 7: Electronic components of the permittivity at zero frequency

Cell	xx	yy	zz	xz
Form I	2.755	2.712	2.804	0
Form II	2.723	2.818	2.642	−0.103
Form III	2.872	2.771	2.979	0
Form IV	2.809	2.886	2.746	0.011
DCI32a	2.733	2.727	2.741	0

5.4 The Discrete Dipole Moment Correlation Function

The dipole moment correlation function was calculated from the cell dipole moments calculated by CP2K at 0.5 fs time intervals. A typical trajectory will contain over 60,000 data points. A discrete version of Equation 17 was used to calculate $F\phi_{ij}$ as Equation 18 seemed to have more numerical problems. The time derivative of the dipole moment correlation function was calculated using numerical differentiation. Various signal processing ideas were used in processing the correlation function. If there is a sequence of calculated dipole moments at regular intervals the sequence can be written as a vector $\mathbf{M_i}$. If the sequence is long enough it can be sampled at different starting points along the sequence, the starting points being separated by a correlation depth, N_{corr}. The correlation depth is chosen to be an integer power of two so that the discrete fast Fourier transform can be used in the calculation of equation 17. In this way a single trajectory can be split into several, hopefully independent trajectories, each of which can be used to calculate a dipole correlation function which can be averaged.

The correlation function can now be written as a vector $\mathbf{C}_{i,j}$ of length N_{corr}. For a perfect simulation of sufficient length and sufficient size, the function should decay to zero in an exponential manner as the phonons scatter have a lifetime related to the decay of the correlation function. In practice the systems that are simulated are small and are often not simulated for long enough to determine the system phonon lifetimes. To compensate for this a window function, $\mathbf{W}$ is applied to the correlation function to ensure that it behaves in a physical manner.

Thus the new correlation function is written as a convolution of the window function with the correlation function;

$$\mathbf{C}'_{i,j} = \mathbf{W} \odot \mathbf{C}_{i,j} \tag{21}$$

The elements of the window function are written as an exponential;

$$\mathbf{W}(k) = e^{-(k-1)a} \tag{22}$$

where the index k runs from 1 to N_{corr}. The parameter a determines the decay rate of the exponential and as the Fourier transform of an exponential is a Lorentzian, it also imposes a Lorentzian line shape onto the signal. In addition to applying a window to the correlation function, the function is padded with zeros so that the total number of points in the signal is pN_{corr} where p is the padding factor.

Two sets of parameters were used to process the correlation function in this work as shown in the table below. A similar approach is taken by Travis.

Table 8: Window parameters used in the calculation of correlation functions

Description	N_{corr}	Decay parameter (a)	Padding factor (p)
Default	8192	5.0	4
Narrow	16384	4.0	2

The default settings are used in the paper unless specified otherwise. The narrow settings are used to give narrower peaks and more definition to the peaks. This latter setting is especially useful for the terahertz region of the spectrum, which otherwise seems to have absorption peaks which are too broad when compared to experiment. The draw back of using the "narrow" settings is that the calculated spectrum is noisier.

5.5 Calculation of the Absorption Spectrum

The trace of the total permittivity tensor is used as an estimate of the permittivity of a powder containing randomly oriented crystallites. From this the absorption coefficient (A) is calculated from the resulting imaginary component of the refractive index (k). In the equations below the dependence on the frequency and hence the wavelength (λ) is implicit. The units of A are inverse distance.

$$\epsilon_{total} = \epsilon_r + \epsilon_{optical} \tag{23}$$

$$(n + ik)^2 = Tr\epsilon_{total} \tag{24}$$

$$A = 4\pi\frac{n}{\lambda} \tag{25}$$

6 Super-cells used in VASP and CP2K calculations

Super-cells for VASP and CP2K calculations were created by extending the initial unit-cell in the a, b or c direction. The choice of which dimension to extend when enlarging the cell was to double the size of the smallest cell dimension. A super-cell is indicated by the use of the “SC” or “DC” designation followed by the polymorph, followed by the number of molecules in the cell and optionally a letter to distinguish between cells. Thus SCII8 refers to a super-cell of Form II with 8 molecules in the super-cell. Where there is some disorder in hydrogen bonding pattern the designation “DC” is used instead of “SC” and therefore DCI32 would refer to a disordered cell of Form I containing 32 molecules. Where more than one super-cell of the same dimensions was created but with different molecular geometries they are labelled a or b.

Table 9 shows the CSD code of the reference unit cell, the designation of the super-cell, the dimensions of the super-cell, and the number of molecules and atoms in the cell for each polymorph considered.

Table 9: Super-cells used in the VASP and CP2K calculations

Polymorph	CSD Code	Cell designation	Cell extension	Molecules	Atoms
Disorder Form I	LCYSTN22	DCI8	1x1x2	8	112
Disorder Form I	LCYSTN22	DCI16	2x1x2	16	224
Disorder Form I	LCYSTN22	DCI32	2x1x4	32	448
Form I	LCYSTN22	SCI8	1x1x2	8	112
Form II	LCYSTN04	SCII8	1x2x1	8	112
Form III	LCYSTN24	SCIII8	1x1x2	8	112
Form IV	LCYSTN26	SCIV8	1x2x1	8	112

7 VASP Results

7.1 Optimisation of all Polymorphs at Experimental Pressures

It is assumed that ambient pressure (0.000 101 GPa) is adequately represented computationally by calculations at 0 GPa. Table 10 shows the optimised unit cell parameters using a pressure appropriate to the corresponding experimental conditions. Table 11 shows the percentage deviations of the calculated unit-cell parameters from the experimental values. Form I shows particularly good agreement between the calculated and experimental unit-cell parameters which were measured at 30 K.

Table 10: VASP optimised unit-cell parameters for polymorphs at experimental pressures*

Polymorph	P(GPa)	a(Å)	b(Å)	c(Å)	$\alpha/°$	$\beta/°$	$\gamma/°$	Vol(Å^3)	Energy(eV)
Form I	0.0	8.087	11.900	5.421	90.0	90.0	90.00	521.65	−323.555
Form II	0.0	9.438	5.199	11.218	90.0	109.0	90.00	520.48	−323.464
Form III	2.6	7.949	10.511	5.348	90.0	90.0	90.00	446.83	−322.858
Form III	4.2	7.874	10.315	5.288	90.0	90.0	90.00	429.46	−322.456
Form IV	1.7	8.073	5.402	10.927	90.0	95.8	90.00	474.08	−323.157

* This table is also reproduced in the main text of the paper

Table 11: VASP optimised unit-cell parameters for polymorphs at experimental pressures - percentage deviations from experiment

Polymorph	CSD	a(%)	b(%)	c(%)	α(%)	β(%)	γ(%)	Vol.(%)
Form I	LCYSTN22[5]	−0.70	−0.31	0.09	0.00	0.00	0.00	−0.91
Form II	LCYSTN04[6]	−0.03	−0.44	−1.05	0.00	−0.02	0.00	−1.51
Form III	LCYSTN24[2]	−1.33	0.22	0.02	0.00	0.00	0.00	−1.10
Form III	LCYSTN25[2]	−1.55	0.17	0.07	0.00	0.00	0.00	−1.32
Form IV	LCYSTN26[2]	−0.40	−0.74	0.10	0.00	0.90	0.00	−1.18

7.2 Optimisation of all Polymorphs at Zero Pressure

Results for Forms I and II have already been shown in the previous section as they we determined experimentally at ambient pressure. But the results are repeated here for comparison purposes. Form III is shown twice in the Tables 12 and 13 because these results were obtained by starting the optimisation with the two different crystal structures obtained at different experimental pressures. The agreement between the results is a measure of the accuracy of the geometry optimisation.

Table 12: VASP optimised unit-cell parameters for polymorphs at zero pressure

Polymorph	a(Å)	b(Å)	c(Å)	$\alpha/°$	$\beta/°$	$\gamma/°$	Vol(Å^3)	Energy(eV)
Form I	8.087	11.900	5.421	90.0	90.0	90.00	521.65	−323.555
Form II	9.438	5.199	11.218	90.0	109.0	90.00	520.48	−323.464
Form III	8.046	11.131	5.481	90.0	90.0	90.00	490.83	−323.269
Form III	8.049	11.129	5.482	90.0	90.0	90.00	491.05	−323.270
Form IV	8.153	5.472	11.372	90.0	95.2	90.00	505.28	−323.372

Table 13: VASP optimised unit-cell parameters for polymorphs at zero pressure - percentage deviations from experiment

Polymorph	CSD	a(%)	b(%)	c(%)	α(%)	β(%)	γ(%)	Vol.(%)
Form I	LCYSTN22[5]	−0.70	−0.31	0.09	0.00	0.00	0.00	−0.91
Form II	LCYSTN04[6]	−0.03	−0.44	−1.05	0.00	−0.02	0.00	−1.51
Form III	LCYSTN24[2]	−0.13	6.13	2.50	0.00	0.00	0.00	8.64
Form III	LCYSTN25[2]	0.64	8.07	3.75	0.00	0.00	0.00	12.83
Form IV	LCYSTN26[2]	0.59	0.54	4.18	0.00	0.28	0.00	5.33

7.3 Variation of Polymorph Geometry with Pressure

The molecular geometries of molecules in the unit cell were calculated as a function of pressure from 0 to 20 GPa. The effects of pressure are shown in Figures 1 to 5.

Figure 1 shows the changes in unit-cell volumes which occur as the pressure is changed from 0 to 20 GPa. The plot shows that unit-cell volume of Form III, which is the most dense polymorph over the whole pressure range. The other polymorph unit-cell volumes are shown relative to it.

There is a discontinuity in the volume of the unit-cell of Form I around 8 GPa

The molecular conformations of the molecules in each polymorph can be distinguished by the C-C-C-S torsion angle, which can be gauche+ (g+), gauche- (g-) or trans (t). Polymorphs II and IV each have two different molecular conformations in the unit-cell. Figures 1 to 5 show the behaviours of the important torsion angles and the C-S-H bond angle as a function of pressure. Only Form I shows discontinuities in the behaviour of these geometric variable at around 8 GPa and around 16 GPa.

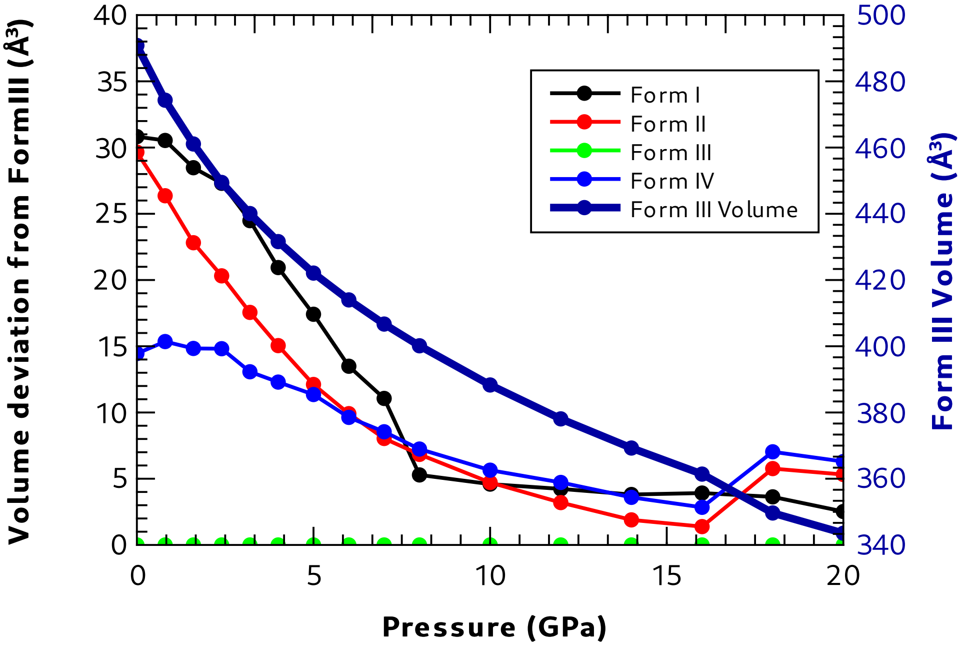

Figure 1: Effect of pressure on polymorph unit-cell volumes. The unit-cell volumes are shown relative to the volume of Form III

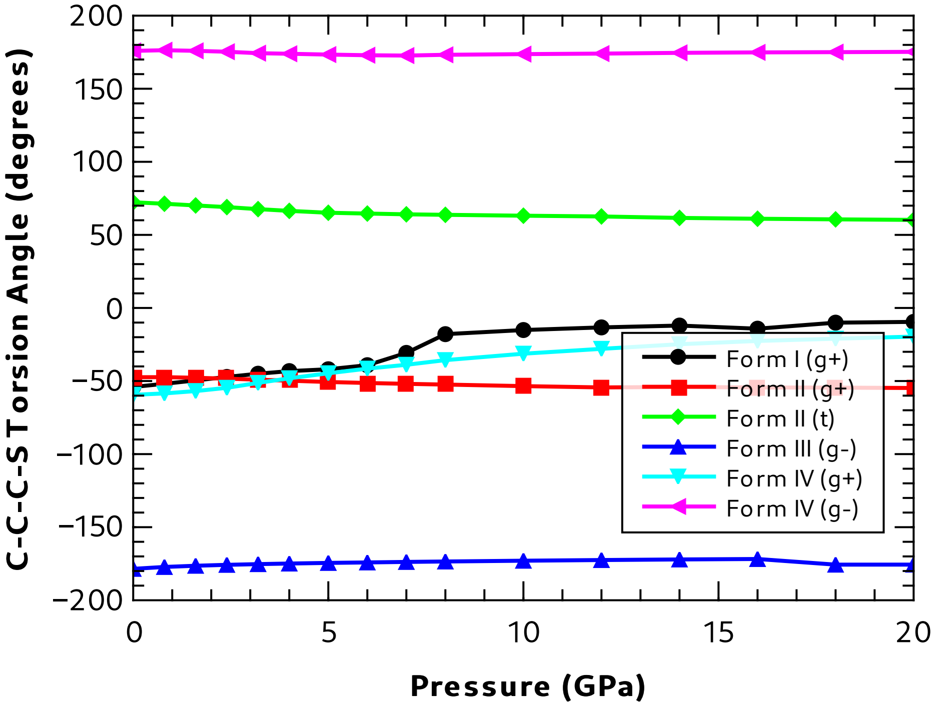

Figure 2: Effect of pressure on the C-C-C-S torsion angle

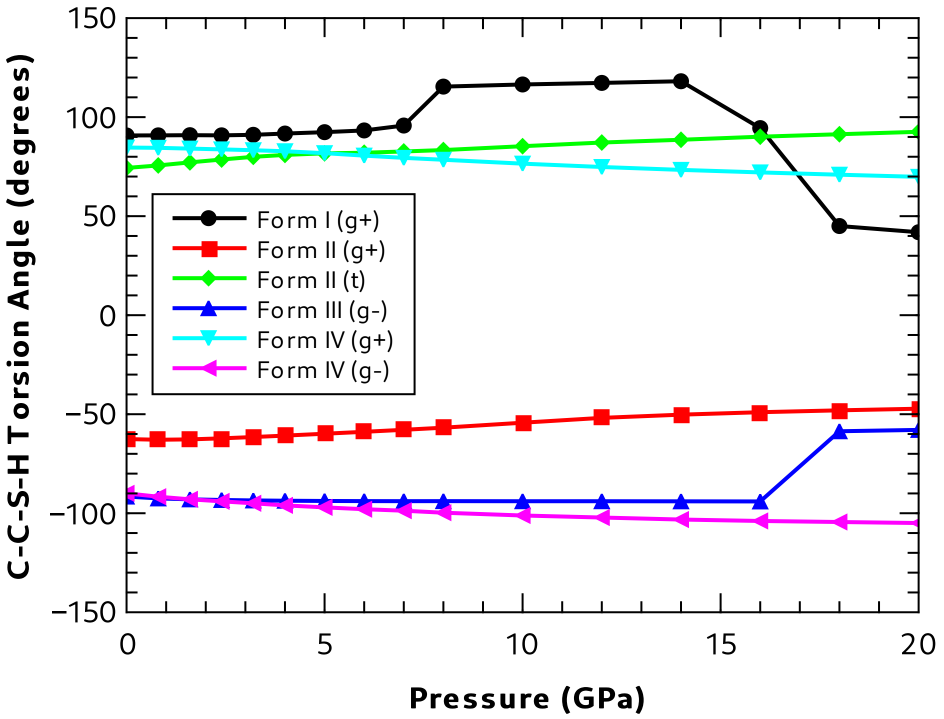

Figure 3: Effect of Pressure on the C-C-S-H torsion angle

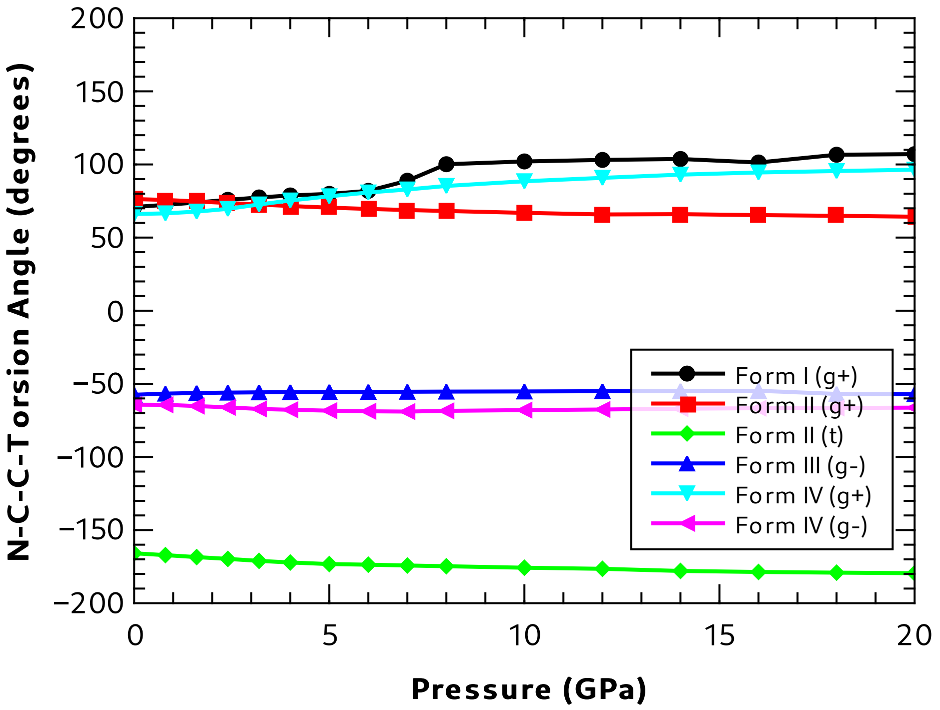

Figure 4: Effect of pressure on the N-C-C-S torsion angle

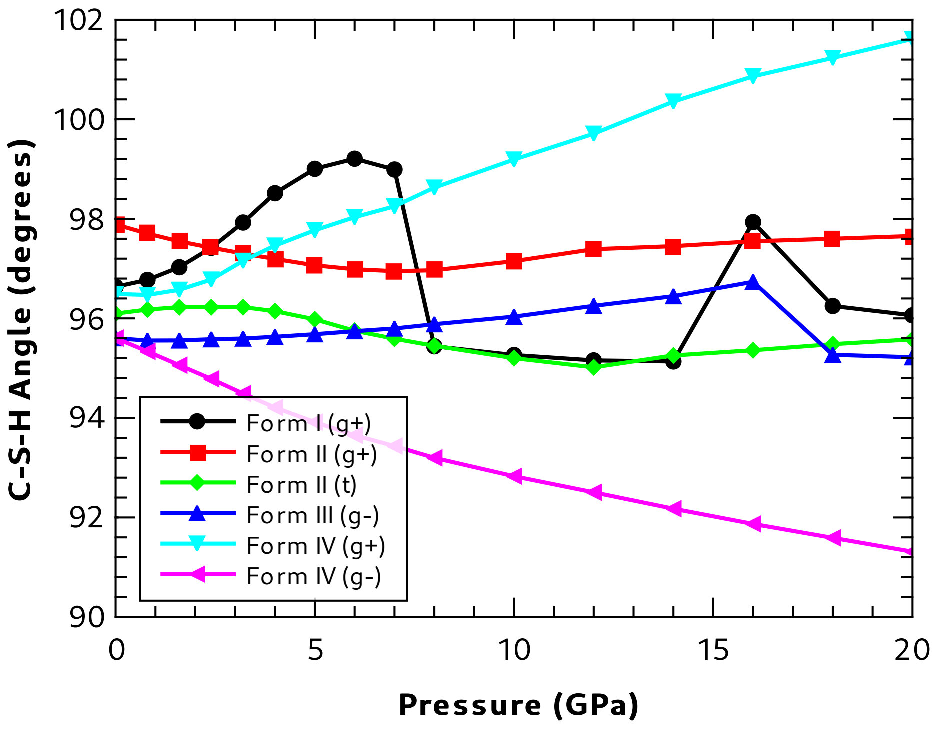

Figure 5: Effect of pressure on the C-S-H angle *
*This table is also reproduced in the main text of the paper

A systematic study was performed of the effect of pressure on each polymorph. Table 14 shows the calculated internal energies of the polymorphs as a function of pressure. Table 15 shows the calculated volume of the polymorph unit-cells as a function of pressure. Using Phonopy the phonon contributions to the free energy were calculated using the harmonic approximation. The vibrational Helmholtz free energy is shown in Table 16 and the total Helmholtz free energy is shown in Table 17.

Table 14: VASP internal energy as a function of pressure*

Pressure (GPa)	Form I	Form II	Form III	Form IV
−1.6	−7799.49	Unstable	−7792.43	−7794.63
−0.8	−7804.08	−7801.56	−7797.23	−7799.69
0.0	−7804.44	−7802.23	−7797.55	−7800.01
0.8	−7802.77	−7800.27	−7795.80	−7798.41
1.6	−7799.40	−7796.61	−7792.81	−7795.38
2.4	−7795.04	−7791.78	−7788.78	−7791.41
3.2	−7789.43	−7786.16	−7784.36	−7786.24
4.0	−7782.45	−7779.77	−7779.36	−7780.81
5.0	−7773.20	−7770.83	−7772.43	−7773.28
6.0	−7762.75	−7761.82	−7765.29	−7764.69
7.0	−7752.60	−7752.40	−7757.76	−7756.08

* Energies are in kJ/mol of molecule

Table 15: VASP volume as a function of pressure*

Pressure (GPa)	FormI	FormII	FormIII	FormIV
−1.6	574.21		549.27	563.98
−0.8	541.83	550.80	514.37	527.05
0.0	521.65	520.48	490.83	505.28
0.8	504.84	500.66	474.30	489.65
1.6	489.56	483.89	461.08	475.91
2.4	476.76	469.78	449.46	464.28
3.2	464.55	457.63	440.07	453.14
4.0	452.54	446.65	431.60	443.89
5.0	439.51	434.21	422.09	433.45
6.0	427.48	423.90	413.99	423.62
7.0	417.76	414.74	406.70	415.24

* Volumes are in Å^3

Table 16: Vibrational Helmholtz free energy at 300K*

Pressure (GPa)	FormI	FormII	FormIII	FormIV
−1.6	255.91		256.13	255.86
−0.8	258.36	258.58	259.14	257.68
0.0	260.06	260.14	261.24	259.98
0.8	261.39	261.38	263.05	261.51
1.6	262.88	262.66	264.58	263.15
2.4	263.88	263.81	265.92	264.34
3.2	265.11	264.97	267.03	265.74
4.0	265.94	265.95	268.13	266.84
5.0	266.72	267.24	269.41	268.28
6.0	267.76	268.30	270.49	270.11
7.0	268.46	269.44	271.57	271.33

* Energies are in kJ/mol of molecule

Table 17: Total Helmholtz free energy at 300K*

Pressure (GPa)	FormI	FormII	FormIII	FormIV
−1.6	−7543.58		−7536.29	−7538.78
−0.8	−7545.72	−7542.98	−7538.09	−7542.01
0.0	−7544.39	−7542.09	−7536.31	−7540.03
0.8	−7541.38	−7538.89	−7532.74	−7536.90
1.6	−7536.52	−7533.95	−7528.24	−7532.24
2.4	−7531.16	−7527.97	−7522.86	−7527.07
3.2	−7524.32	−7521.19	−7517.33	−7520.50
4.0	−7516.52	−7513.82	−7511.23	−7513.97
5.0	−7506.48	−7503.59	−7503.02	−7504.99
6.0	−7494.99	−7493.52	−7494.81	−7494.58
7.0	−7484.14	−7482.96	−7486.19	−7484.74

* Energies are in kJ/mol of molecule

The internal energy relative to Form I is shown in Figure 6 and the relative total Helmholtz free energies at 300K as a function of pressure are shown in Figure 7.

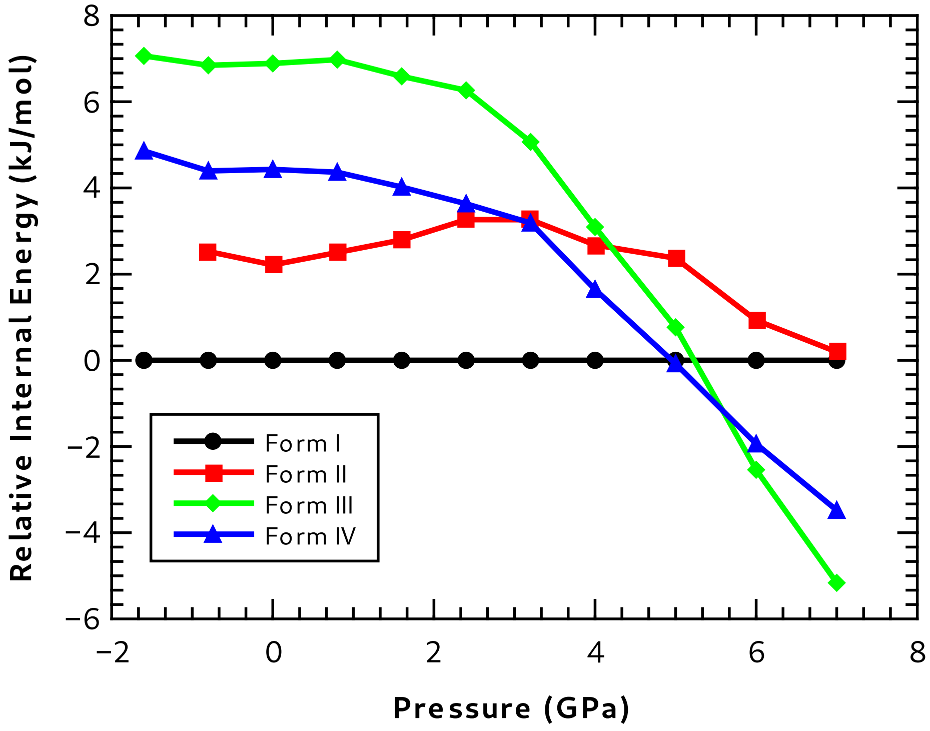

Figure 6: Relative internal energy with respect to Form I as a function of pressure

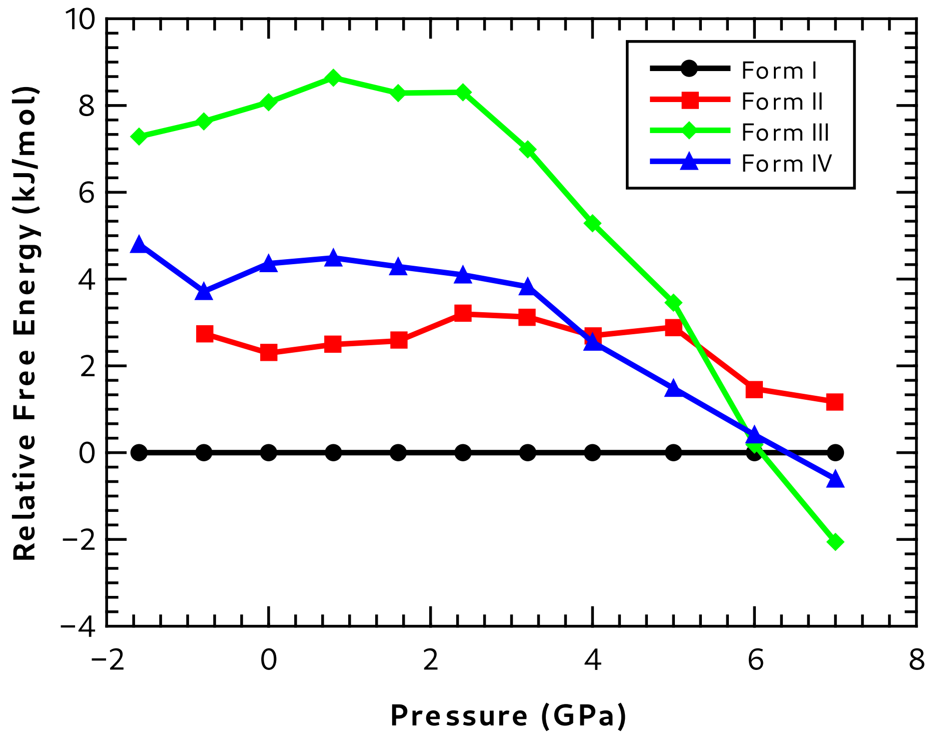

Figure 7: Relative Helmholtz Energy with respect to Form I as a function of pressure

7.4 VASP Study of SH...S and SH..O Hydrogen Bonding in Form I

The calculations summarised in Table 18 show the results of optimising a single unit-cell maintaining the experimental space group symmetry ($P2_12_12_1$). The hydrogen bonding pattern SH...S refers to the low temperature bonding pattern observed experimentally. The SH...O bonding pattern refers to a unit cell created from the experimental one with the C-C-S-H torsion angle rotated so that the the S-H is directed at a nearby oxygen atom and the unit cell and atoms are subsequently optimised to lower the energy

Table 18: Energies and optimised unit-cell parameters for different H-bonding patterns

Dispersion	H-bond	Energy(eV)	Δ E(kJ mol^{-1})	a(Å)	b(Å)	c(Å)	V(Å^3)
None	SH...S	−319.498	0.0	8.299	12.154	5.499	554.66
None	SH...O	−319.399	2.4	8.210	12.392	5.481	557.64
GD3/BJ	SH...S	−323.540	0.0	8.156	11.872	5.415	524.36
GD3/BJ	SH...O	−323.315	5.4	7.894	12.222	5.426	523.51

7.5 Behaviour of Torsion Angles

Table 19 summarises and compares the significant torsion angles found by experiment and determined using VASP optimisations of the experimental unit cells. The C-C-S-H torsion angle is a significant coordinate in the description of the different polymorphs of cysteine. The values found in the experimental column refer to the values found in the associated CSD code crystal structure. In the case of Form I, the high temperature structure has disorder and both torsion angles are shown. The VASP torsion angles for this polymorph refer to the optimisations of the unit-cells with SH...S and SH...O hydrogen bonding patterns. The VASP calculations were performed at the pressures indicated. In the case of Form IV there are no reported hydrogens attached to sulfur and the VASP results are optimised from the assumed hydrogen positions as discussed in Section 1.

Table 19: Torsion angles from experiment and from VASP optimisations

Form	CSD Code	Torsion angle	Experiment	VASP
Form I	LCYSTN22[a]			
30K, 0.0GPa		C-C-S-H	86.97	90.75
		N-C-C-S	70.63	70.91
		C-C-C-S	−53.16	−54.07
Form I	LCYSTN23[b]			
293K, 0.0GPa	S-H...S	C-C-S-H	74.20	90.75
		N-C-C-S	65.32	70.91
		C-C-C-S	−58.69	−54.07
	S-H...O	C-C-S-H	−67.31	−69.17[c]
		N-C-C-S	65.32	67.20[c]
		C-C-C-S	−58.69	−58.39[c]
Form II	LCYSTN04			
120K, 0.0GPa	Mol A[a]	C-C-S-H	80.78	74.35
		N-C-C-S	−170.16	−165.86
		C-C-C-S	70.15	72.31
	Mol B[d]	C-C-S-H	−64.57	−62.54
		N-C-C-S	74.40	76.34
		C-C-C-S	−48.92	−47.39
Form III	LCYSTN24[d]			
293K, 2.6GPa		C-C-S-H	−71.54	−92.22
		N-C-C-S	−56.55	−55.65
		C-C-C-S	−175.69	−175.51
Form III	LCYSTN25[d]			
293K, 4.2GPa		C-C-S-H	−66.12	−93.50
		N-C-C-S	−57.30	−55.56
		C-C-C-S	−174.66	−174.77
Form IV	LCYSTN26			
293K, 1.7GPa	Mol A[a]	C-C-S-H	Unkown	83.37
		N-C-C-S	70.99	66.79
		C-C-C-S	−53.54	−57.79
	Mol B[d]	C-C-S-H	Unkown	−92.50
		N-C-C-S	−67.36	−65.44
		C-C-C-S	170.84	175.63

[a] This molecule belongs to an S-H...S hydrogen bonding pattern
[b] Molecules in this structure are disordered
[c] These torsion angles were taken from optimisation of a unit cell with an S-H..O hydrogen bonding pattern
[d] This molecule belongs to an S-H...O hydrogen bonding pattern

7.6 Form I Unit-Cells and Super-Cells

A series of super-cells of Form I were created as described in Table 9; DCI8, DCI16 and DCI32. For each of these super-cells, two different distributions (a and b) of the C-C-S-H torsion angle was chosen

which equal number of torsion angles with values of 90.75 degrees or −69.17 degrees as seen in the high temperature Form I X-ray structure. The atomic positions within each cells were then optimised and the vibrational absorption spectra were calculated.

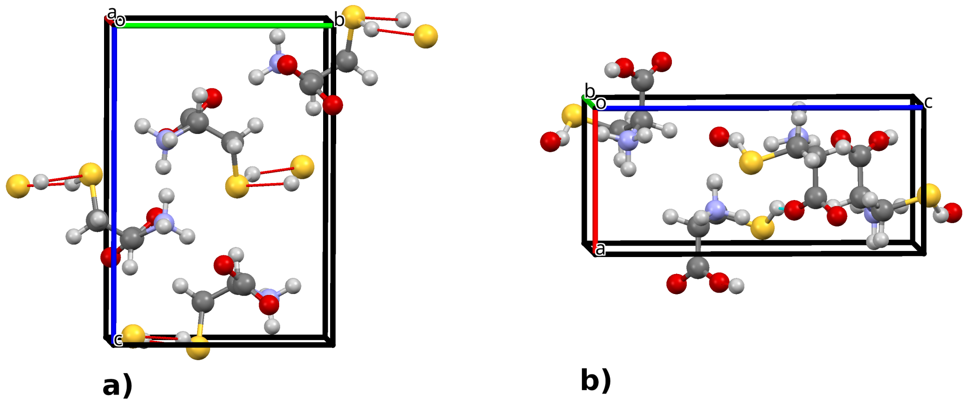

Figure 8: Optimised unit-cell with SH...S hydrogen bonding motif. a) view along a-axis b) view along b-axis

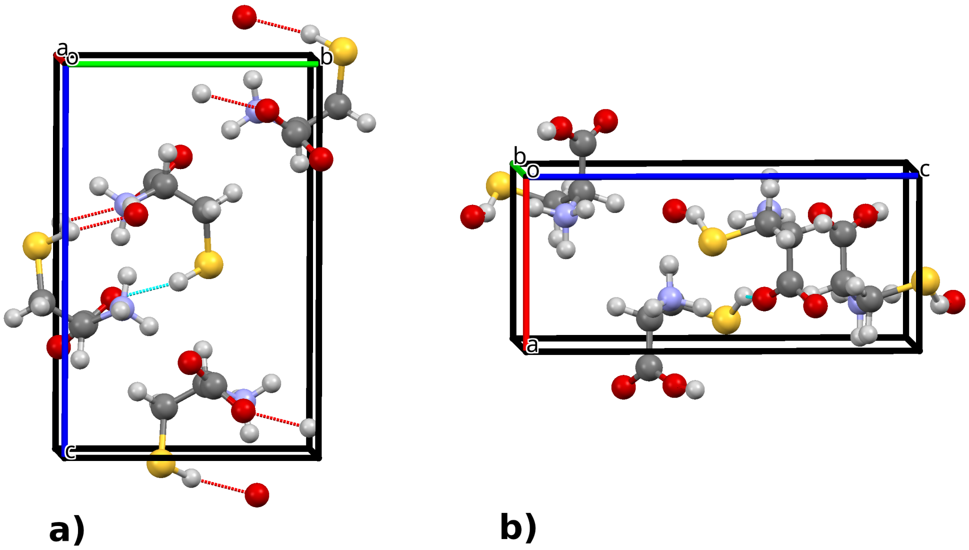

Figure 9: Optimised unit-cell with SH...O hydrogen bonding motif. a) view along a-axis b) view along b-axis

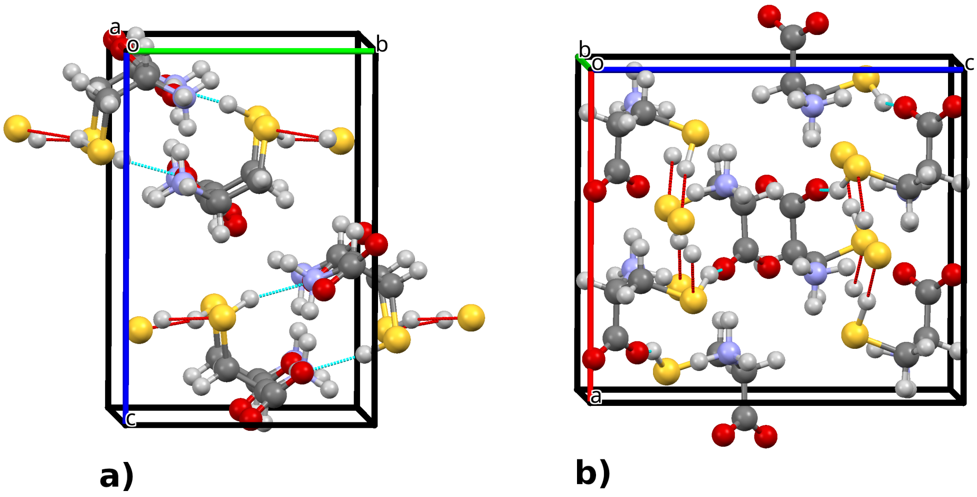

Figure 10: DCI8a a) view along a-axis b) view along b-axis

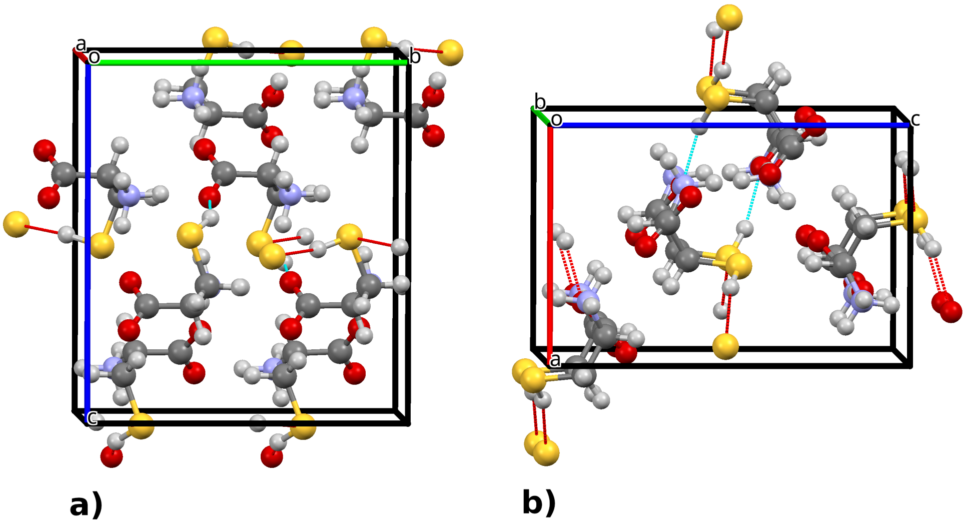

Figure 11: DCI8b a) view along a-axis b) view along b-axis

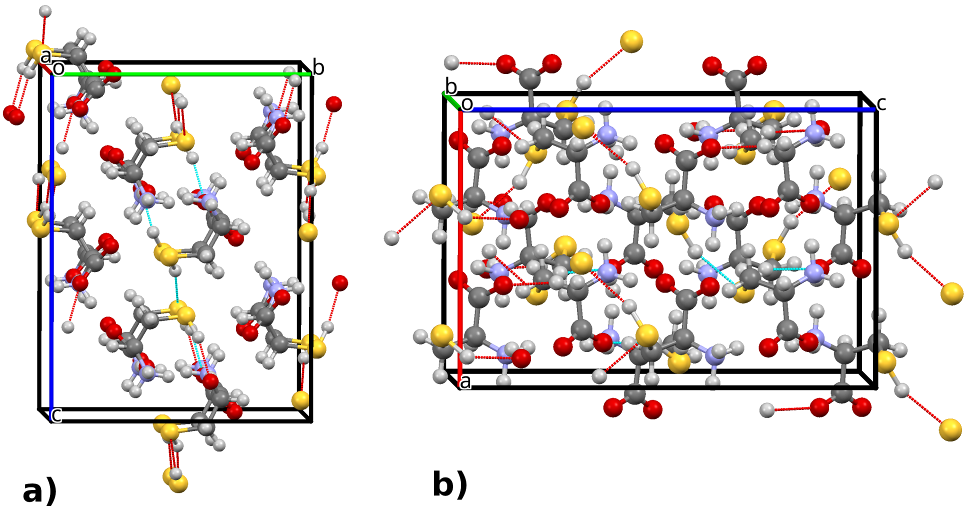

Figure 12: DCI16a a) view along a-axis b) view along b-axis

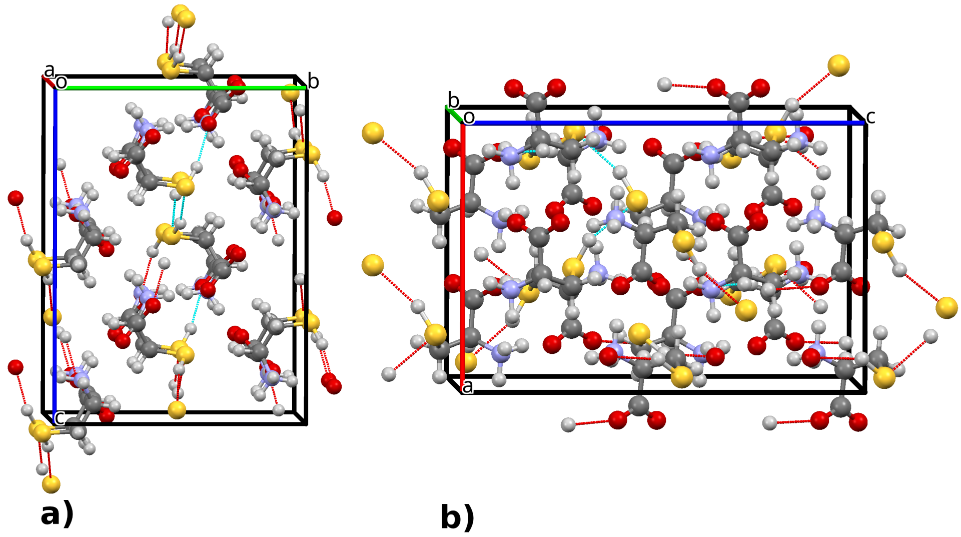

Figure 13: DCI16b a) view along a-axis b) view along b-axis

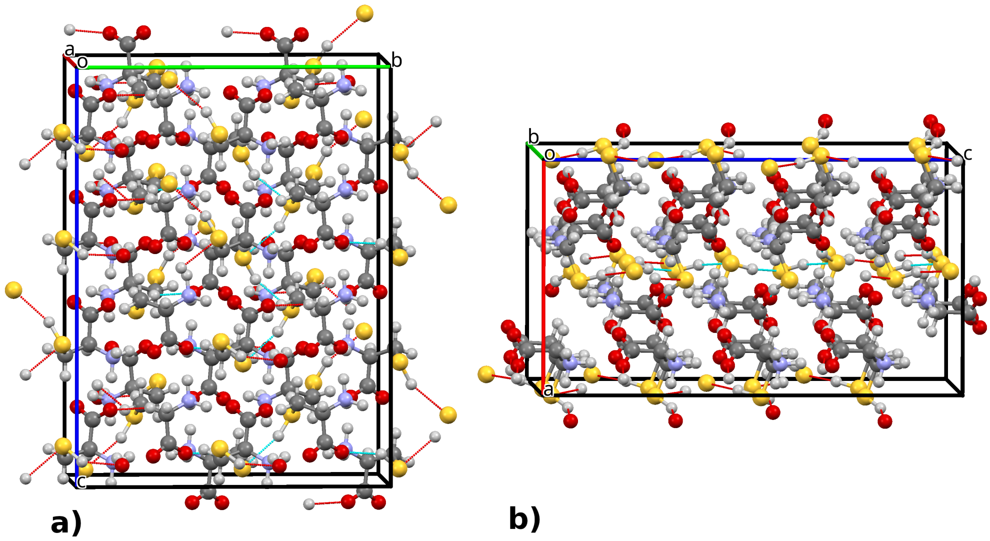

Figure 14: DCI32a a) view along a-axis b) view along b-axis

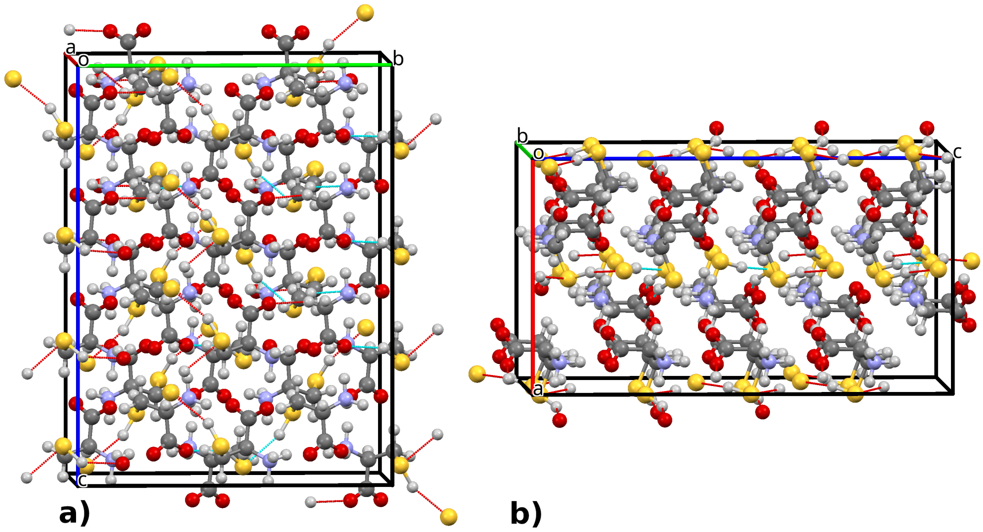

Figure 15: DCI32b a) view along a-axis b) view along b-axis

7.7 Calculated Infrared and THz Frequencies for SH...S and SH...O Hydrogen-bonding Motifs

7.7.1 Frequencies

The frequencies presented here are transverse optical (TO) frequencies calculated by the PDielec package[15] from the VASP output after the centre of mass motion was projected from the dynamical matrix. The atomic masses used were an average over the natural abundance of each elemental isotope.

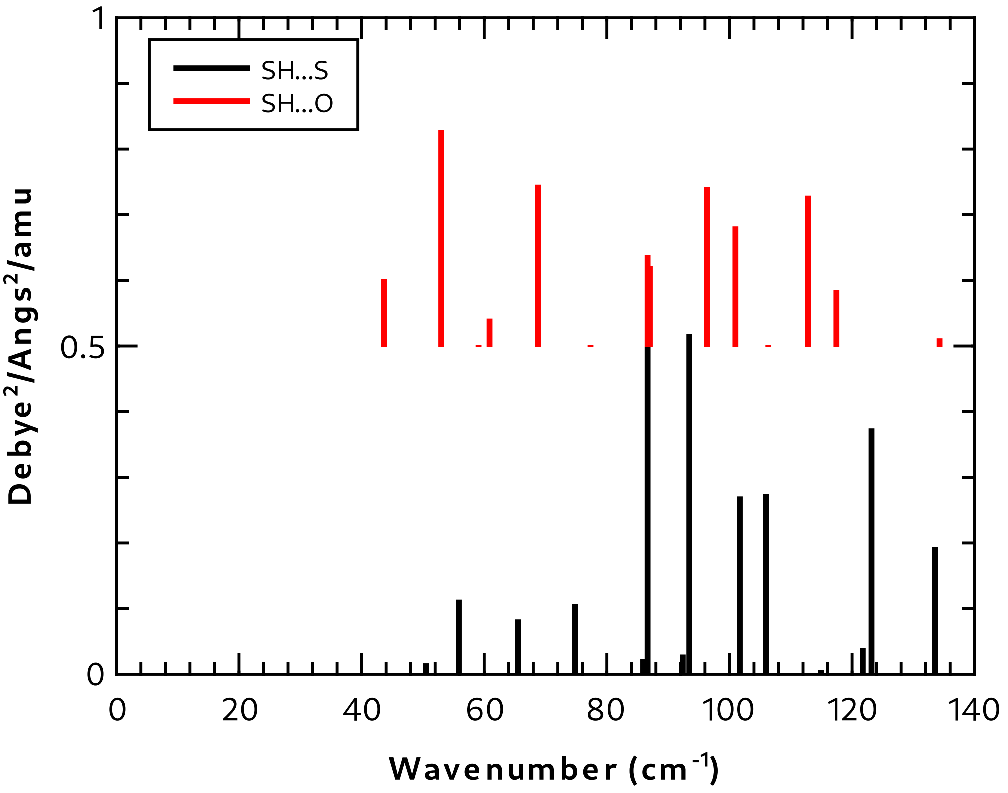

Figure 16: Calculated THz frequencies for SH...S and SH..O hydrogen-bonding motifs

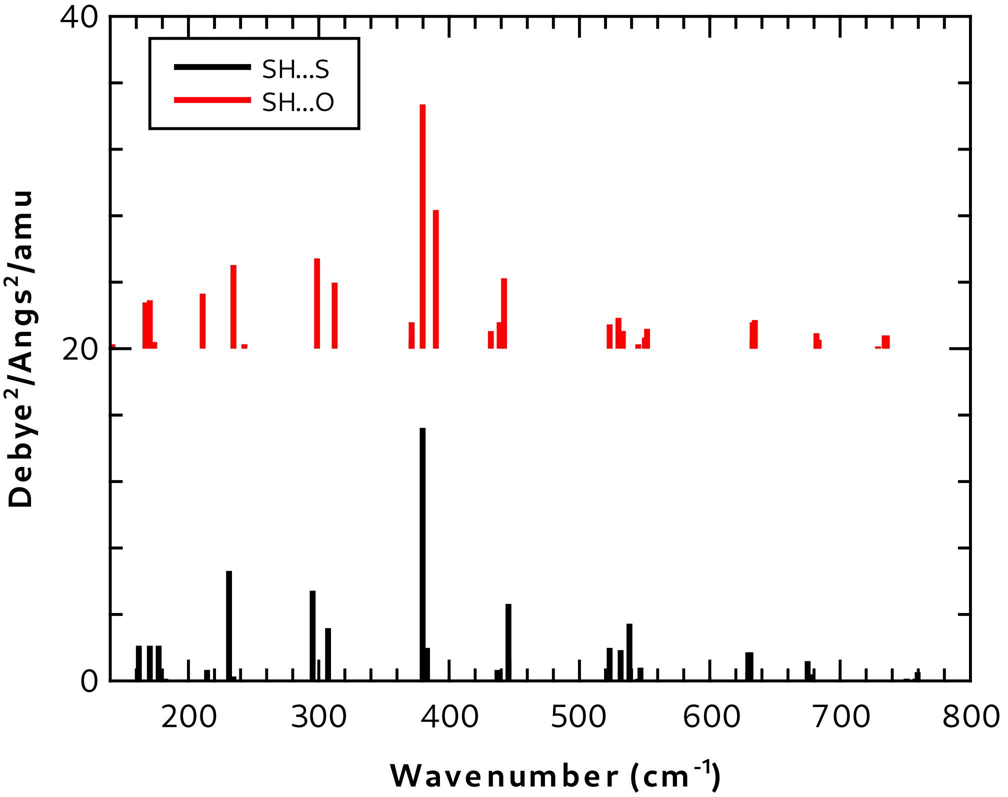

Figure 17: Calculated low frequencies for SH...S and SH..O hydrogen-bonding motifs

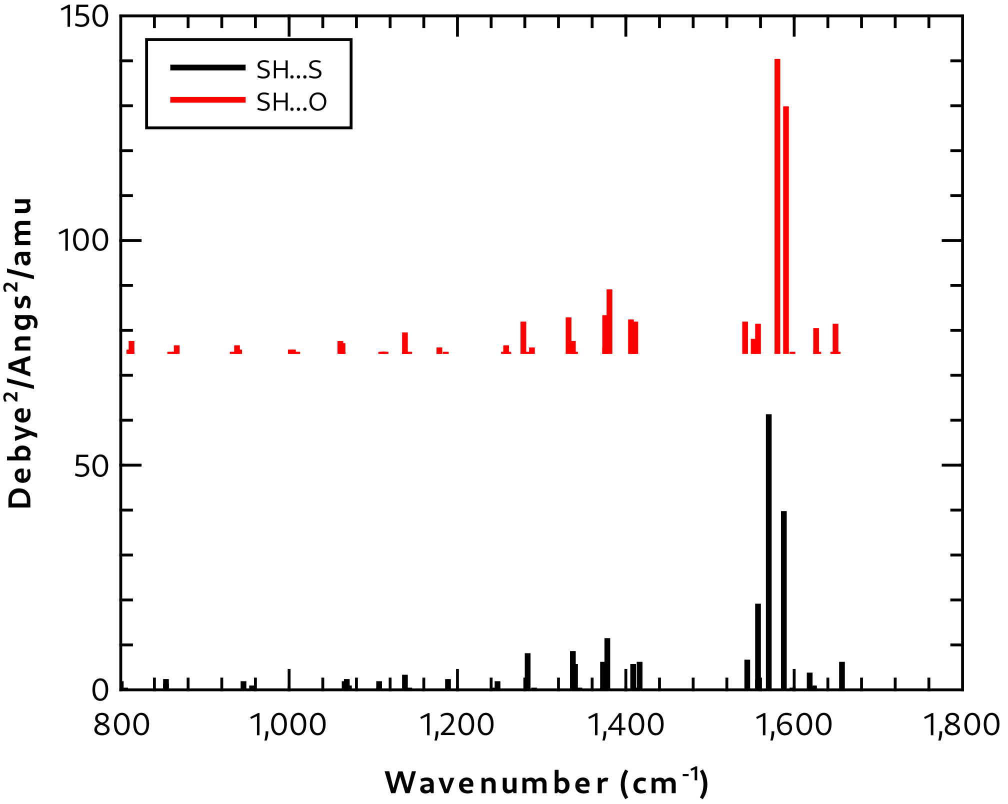

Figure 18: Calculated intermediate frequencies for SH...S and SH..O hydrogen-bonding motifs

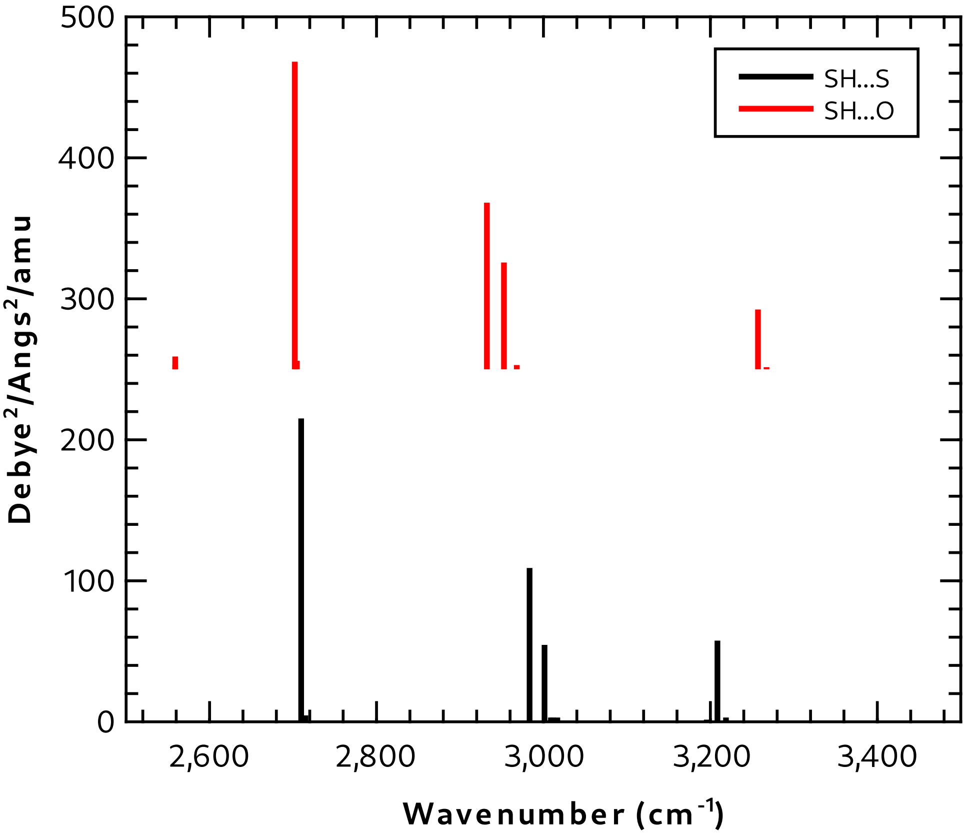

Figure 19: Calculated high frequencies for SH...S and SH..O hydrogen-bonding motifs

7.7.2 IR Absorption

Calculations of the spectra were performed with PDielec package. All results presented in this section assumed 10% by volume of spherical form I crystallites of L-cysteine were packed in a PTFE support. A line width of 5 cm^{-1} was assumed for all absorptions. The Maxwell-Garnett effective medium theory was applied.

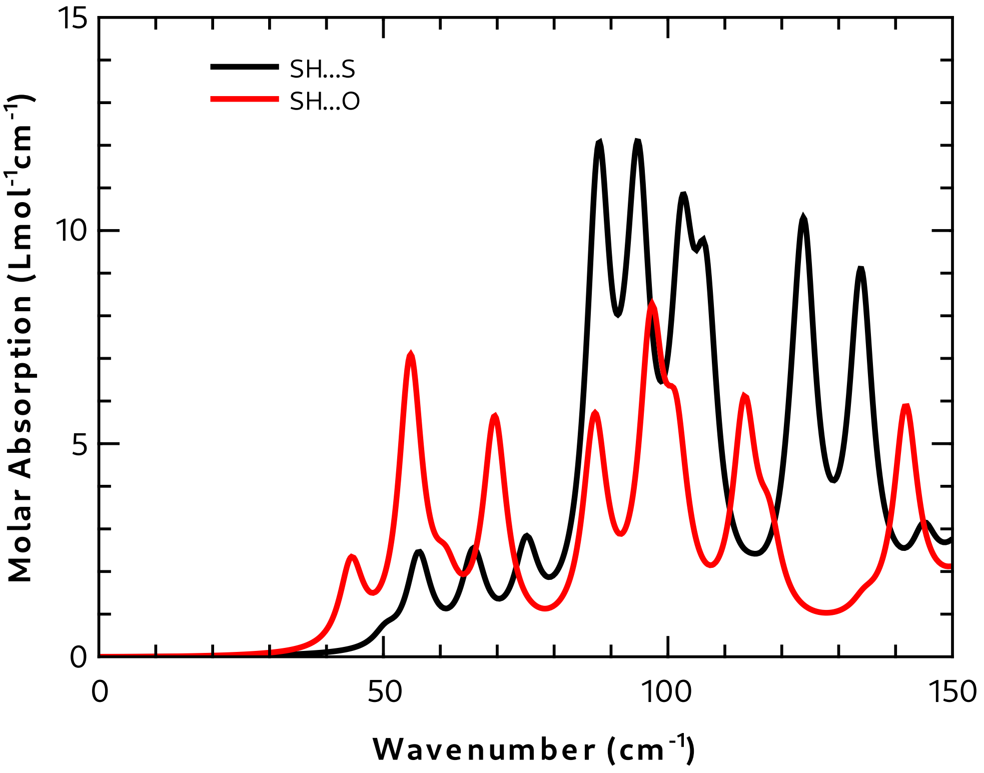

Figure 20: Calculated THz spectra for SH...S and SH..O hydrogen-bonding motifs

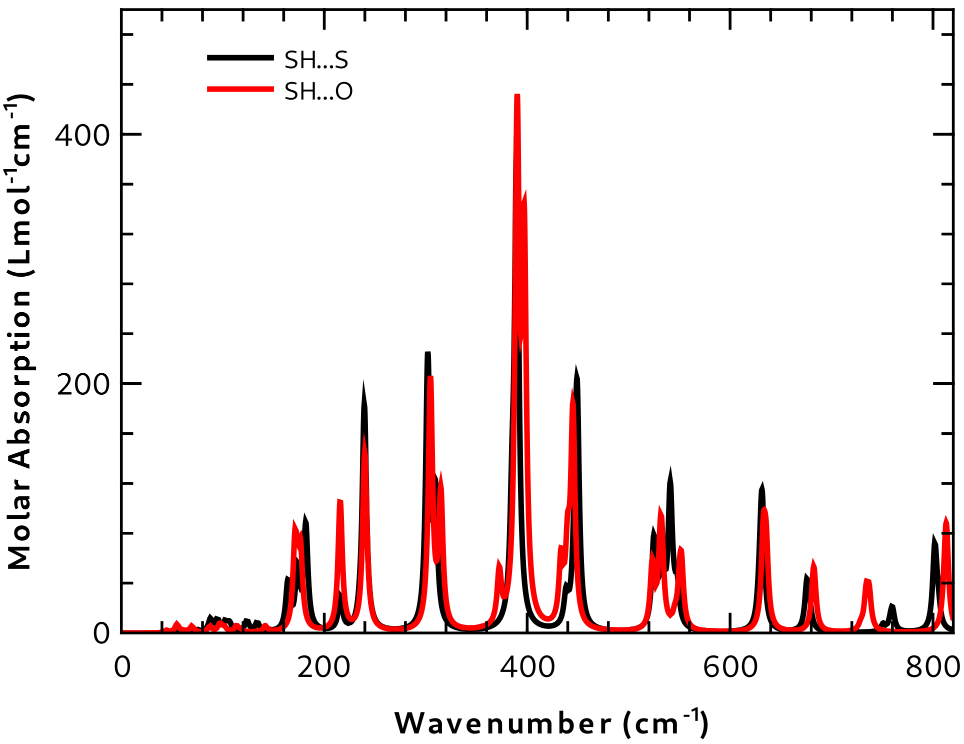

Figure 21: Calculated low frequency spectra for SH...S and SH..O hydrogen-bonding motifs

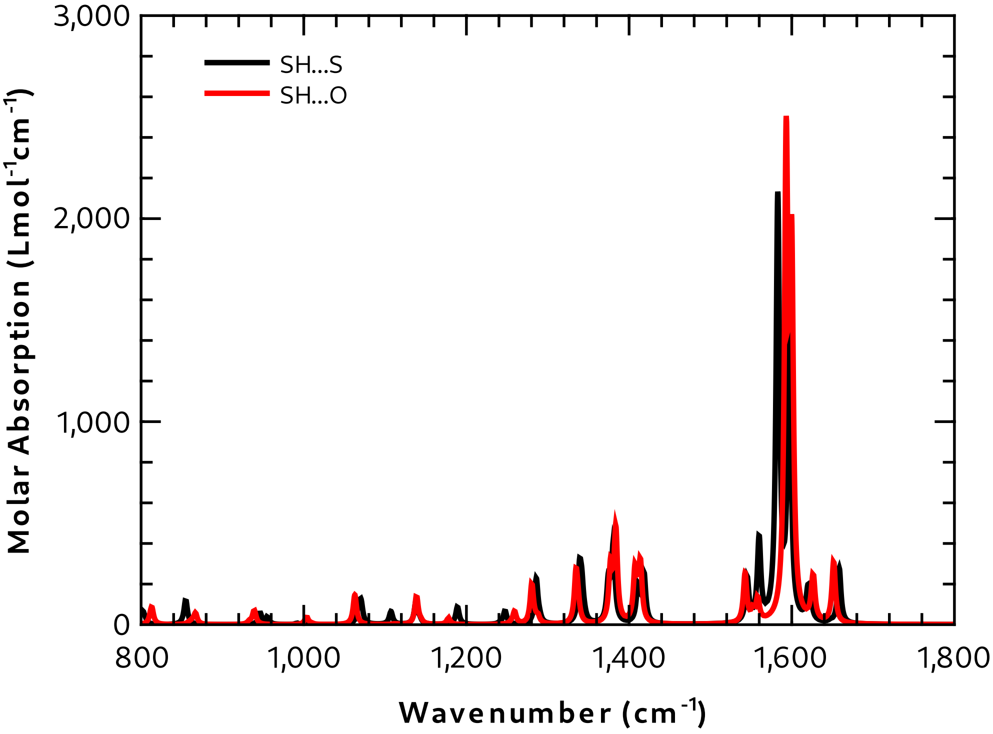

Figure 22: Calculated intermediate frequency spectra for SH...S and SH..O hydrogen-bonding motifs

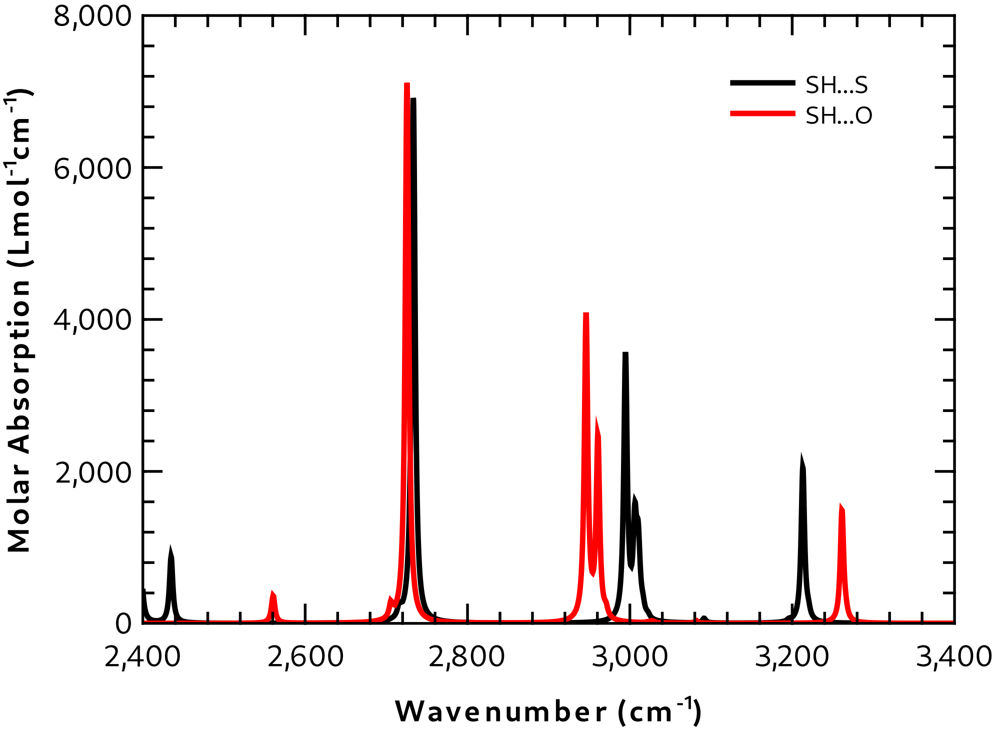

Figure 23: Calculated high frequency spectra for SH...S and SH..O hydrogen-bonding motifs

7.8 Super-cell Infrared Spectra

7.8.1 Comparison of DCI8a and DCI8b

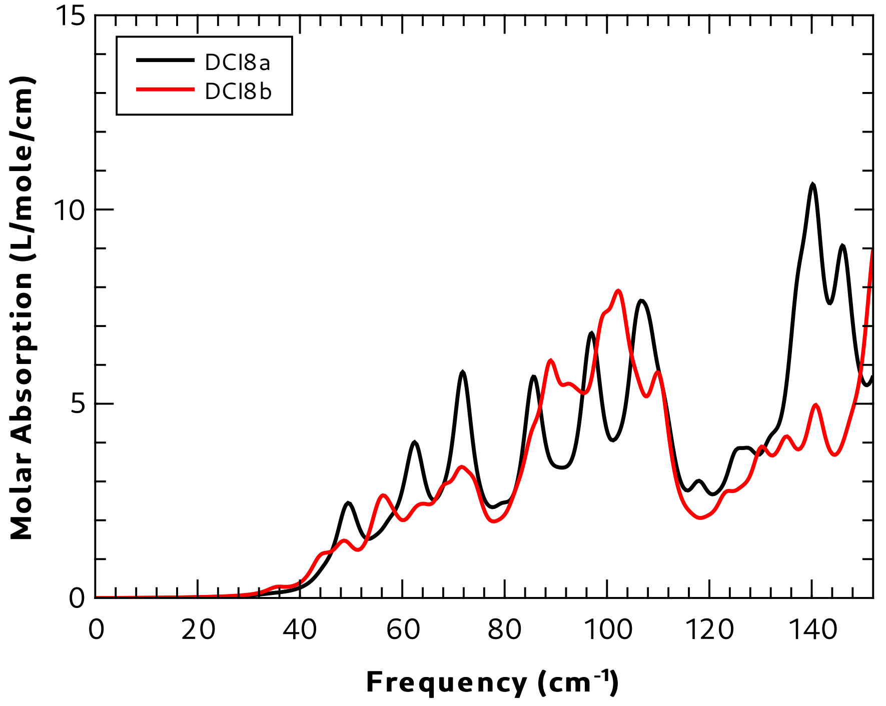

Figure 24: Calculated THz spectra for DCI8a and DCI8b

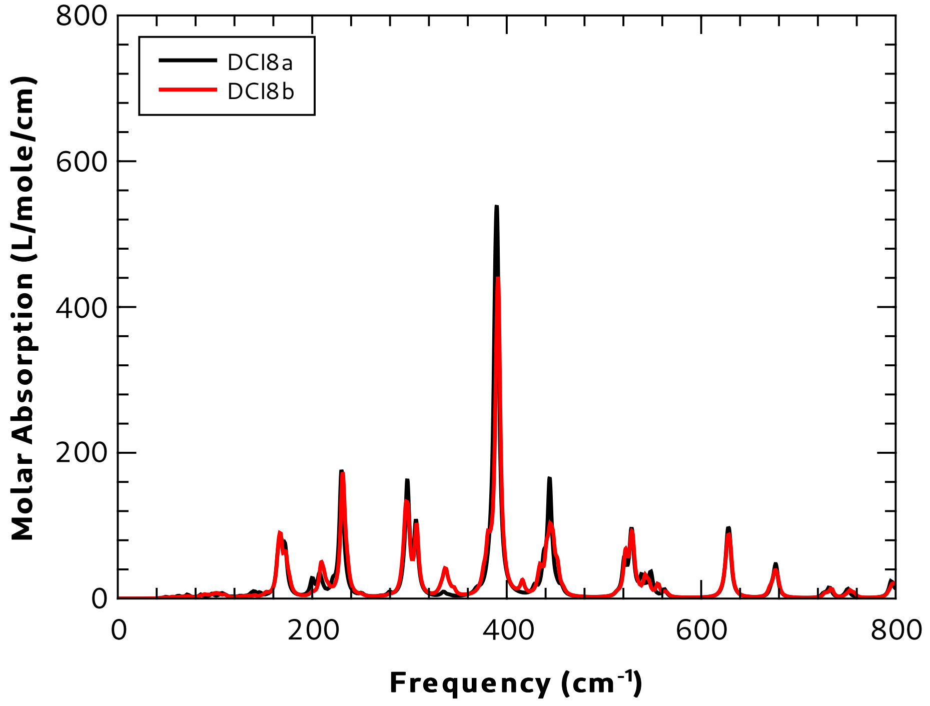

Figure 25: Calculated low frequency spectra for DCI8a and DCI8b

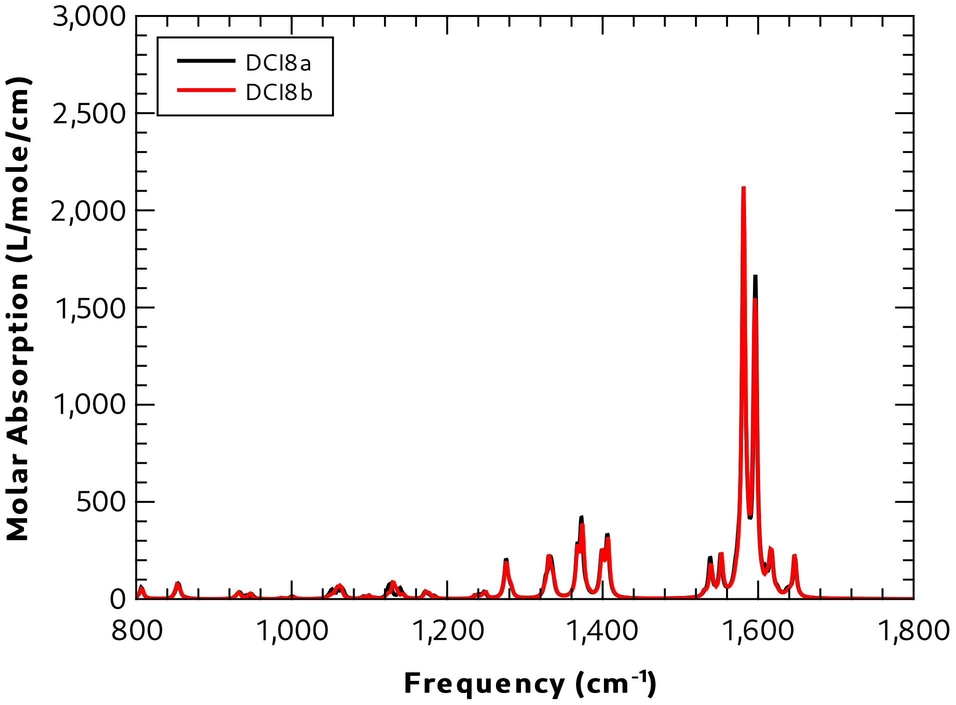

Figure 26: Calculated intermediate frequency spectra for DCI8a and DCI8b

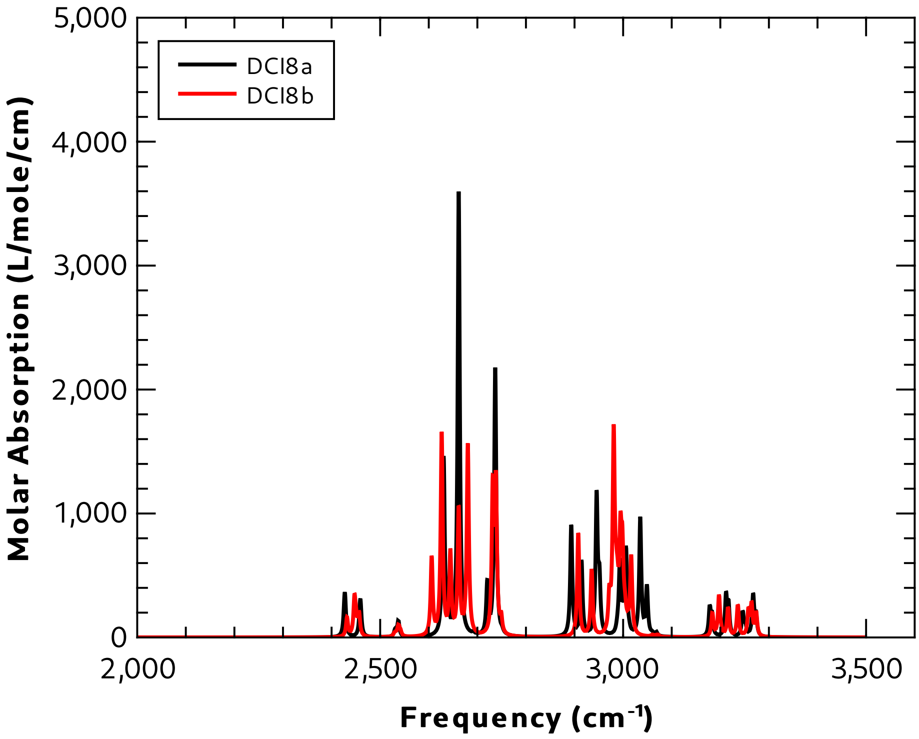

Figure 27: Calculated high frequency spectra for DCI8a and DCI8b

7.8.2 Comparison of DCI16 and DCI16b

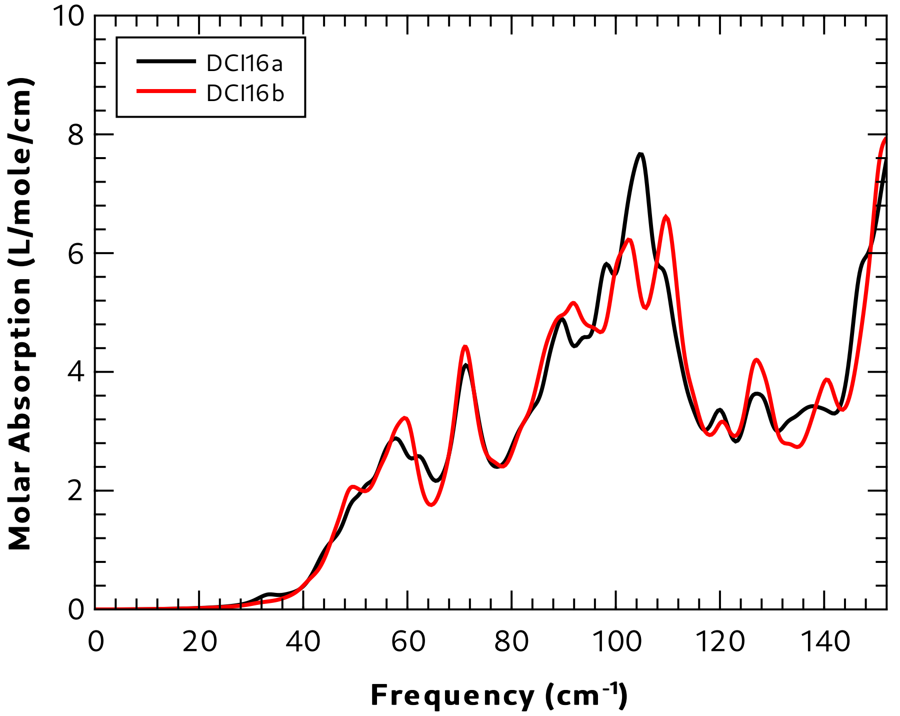

Figure 28: Calculated THz spectra for DCI16a and DCI16b

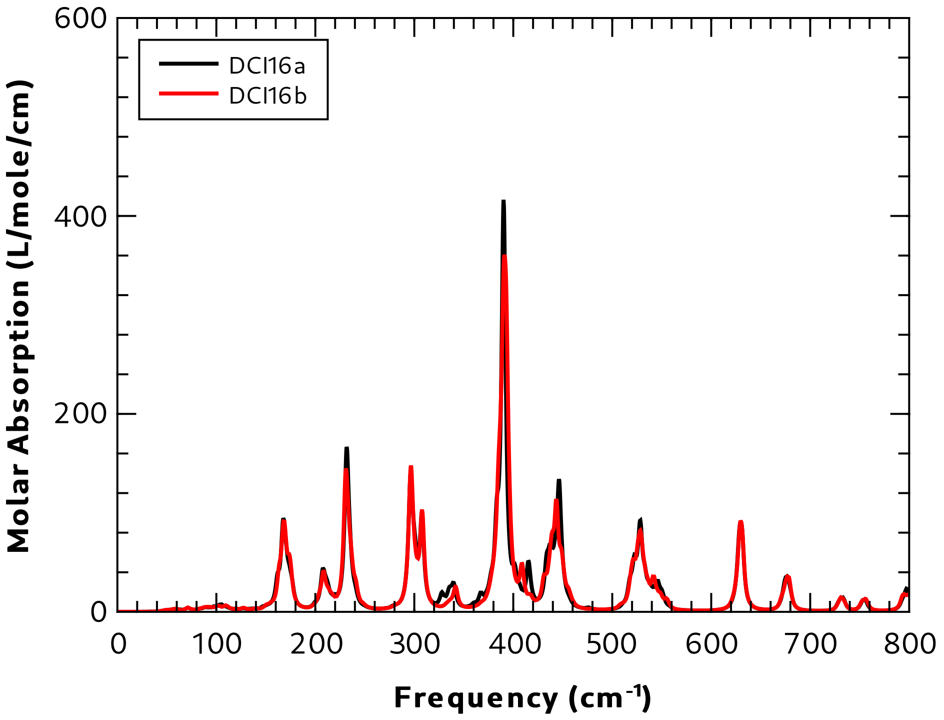

Figure 29: Calculated low frequency spectra for DCI16a and DCI16b

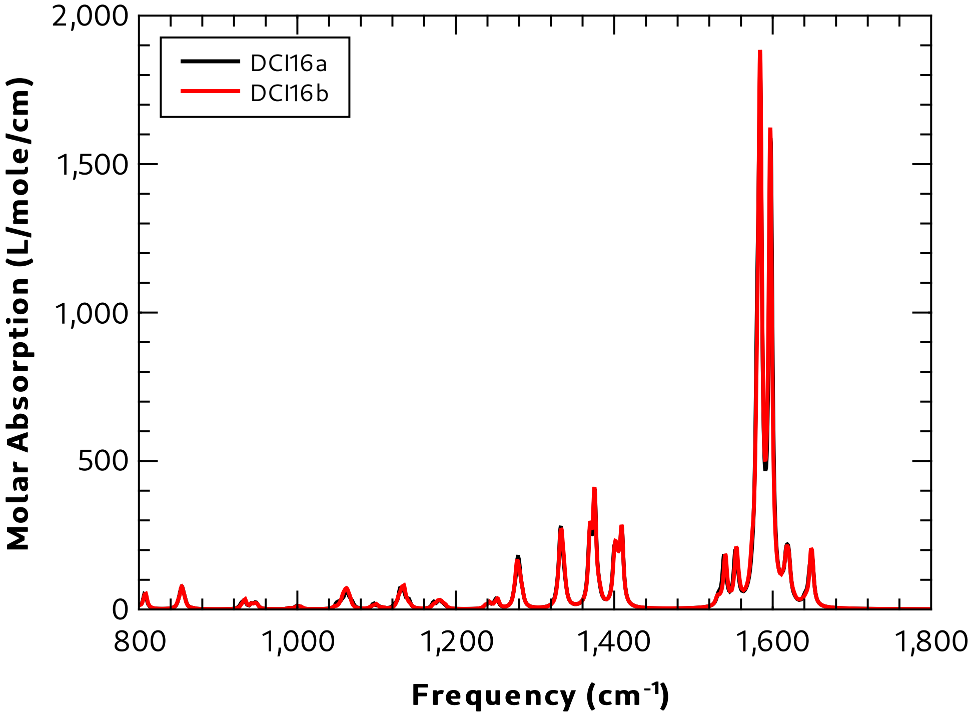

Figure 30: Calculated intermediate frequency spectra for DCI16a and DCI16b

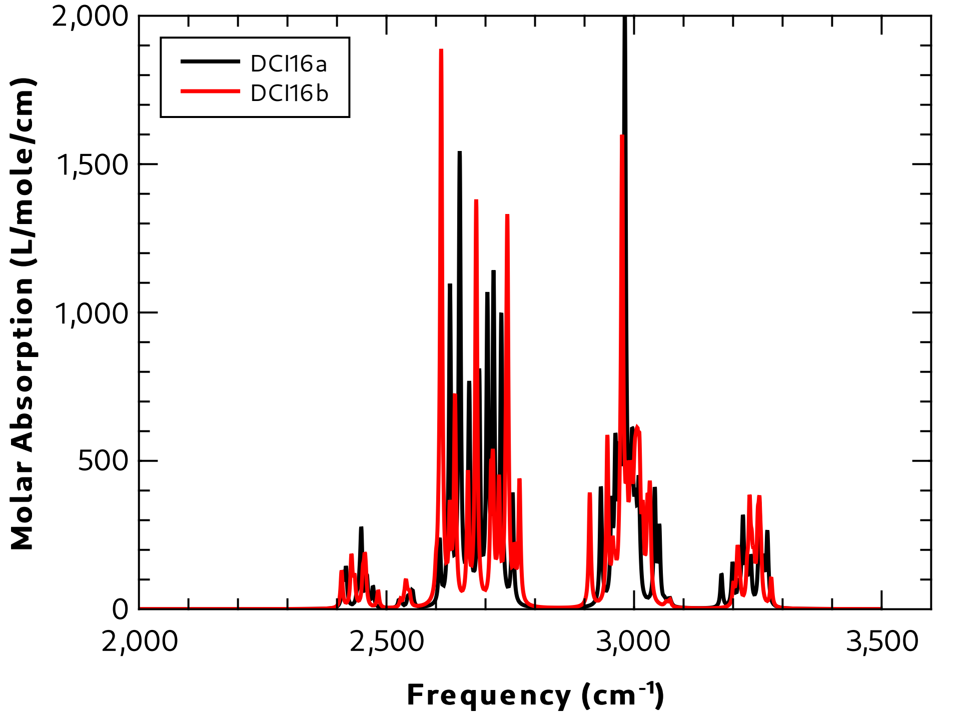

Figure 31: Calculated high frequency spectra for DCI16a and DCI16b

7.8.3 Comparison of DCI32a and DCI32b

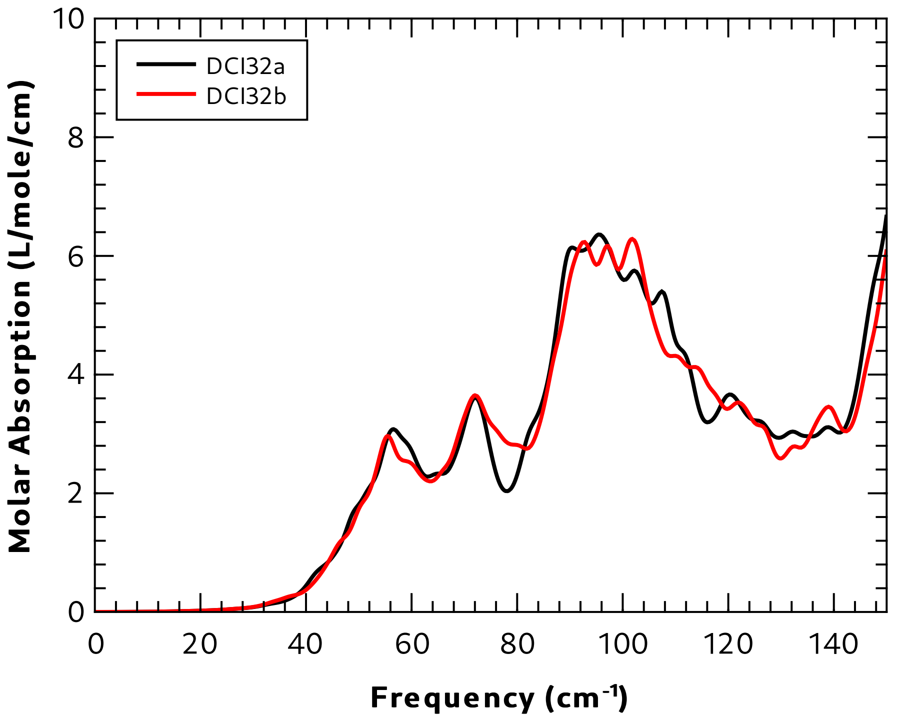

Figure 32: Calculated THz spectra for DCI32a and DCI32b

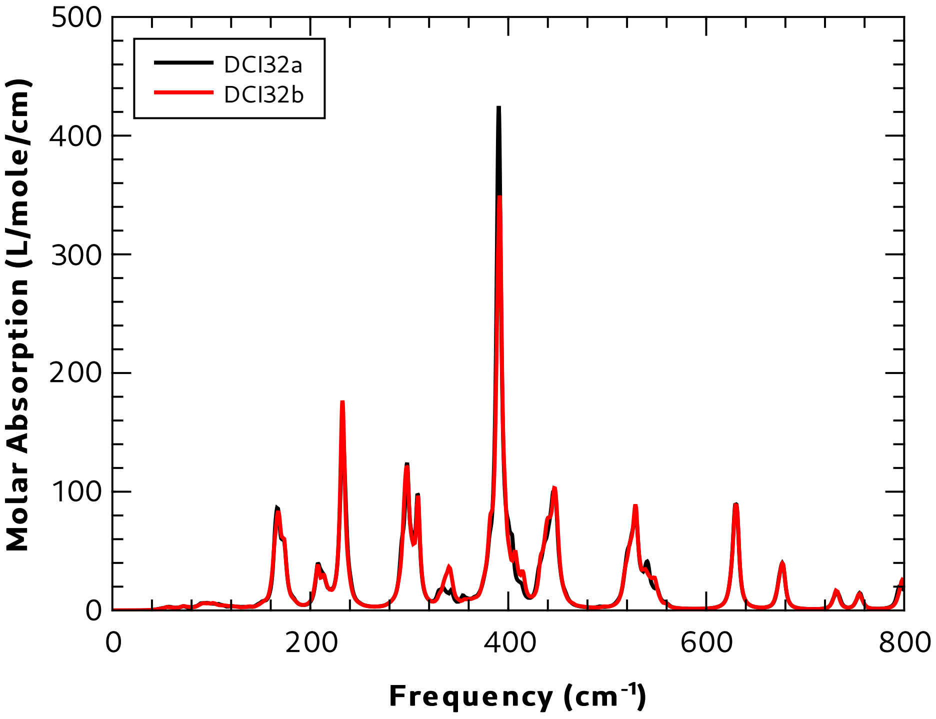

Figure 33: Calculated low frequency spectra for DCI32a and DCI32b

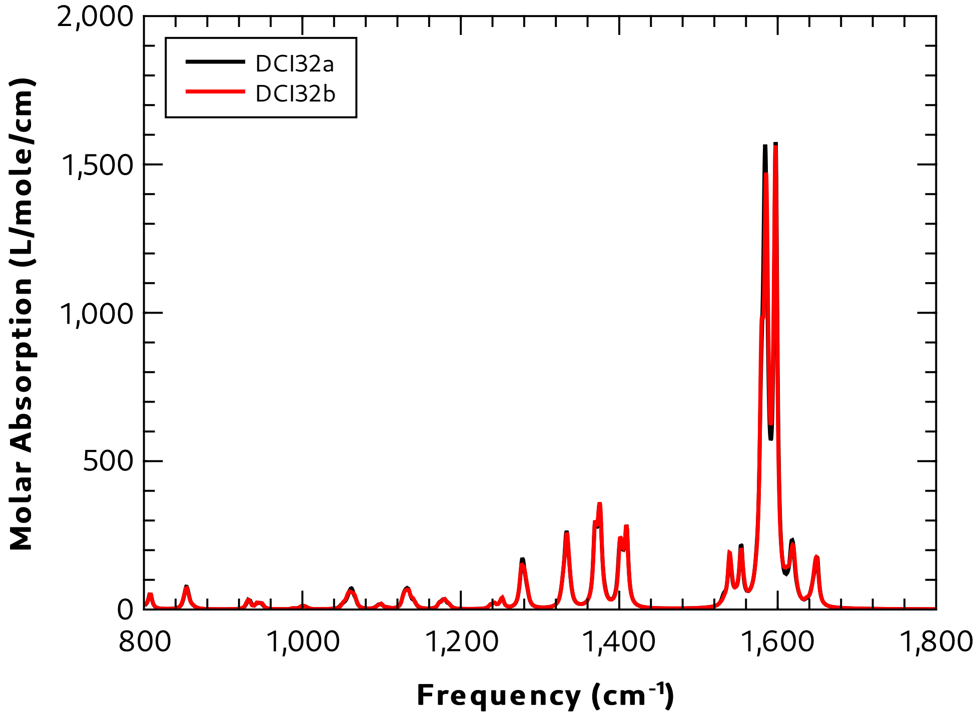

Figure 34: Calculated intermediate frequency spectra for DCI32a and DCI32b

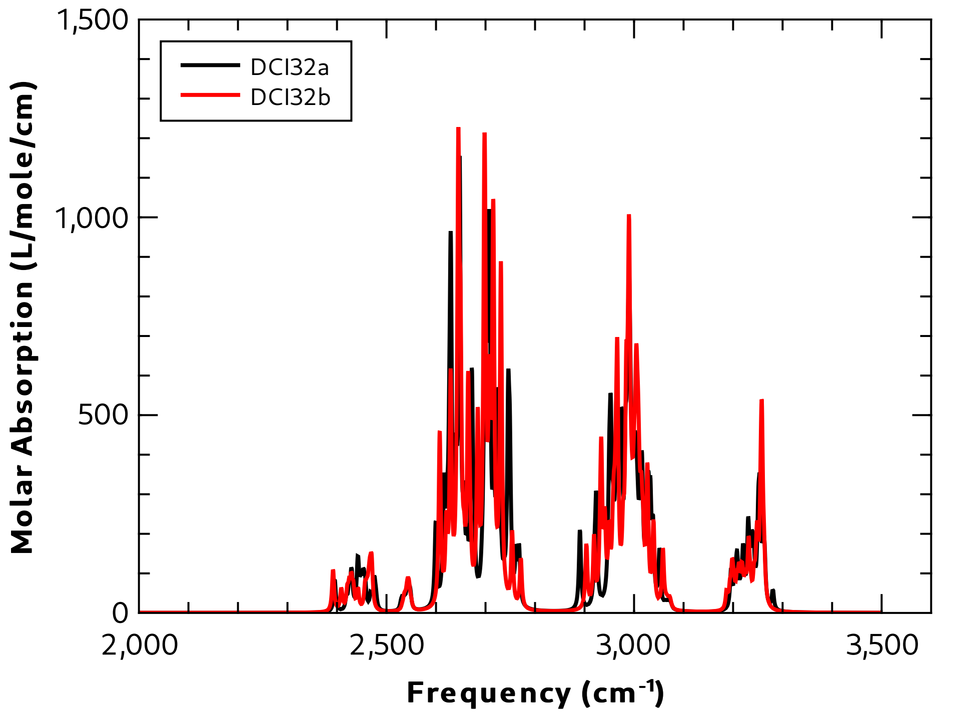

Figure 35: Calculated high frequency spectra for DCI32a and DCI32b

8 CP2K Results

8.1 Molecular Dynamics Calculations of Super-cells

After NPT equilibration of the cell dimensions under the experimental conditions of pressure and temperature, the averaged cell-dimension for super-cells of all known polymorphs as well as that based on the DCI32a (referred to as DCI32 for the reporting of MD results) super-cell were measured to determine the unit-cell parameters to use for subsequent NVT calculations. The results are shown in Table 20. Figures 36 to 39 shows the fluctuations in cell dimensions observed during the NPT calculations along with the arrows which indicate the average values used for the NVT calculations.

Figures 40 to 42 show the fluctuations in cell dimensions of NPT calculations of the DCI32 super-cells at temperatures of 88, 300 and 350 K respectively.

Table 20: Average super-cell parameters calculated by CP2K

Cell	T/K	P/GPa	a /Å	b /Å	c /Å	$\alpha/°$	$\beta/°$	$\gamma/°$
SCI8	88	0.0	10.859	8.084	12.091	90.0	90.0	90.0
SCI8	300	0.0	10.902	8.167	12.056	90.0	90.0	90.0
SCII8	120	0.0	9.361	10.463	11.307	90.0	104.4	90.0
SCIII8	300	2.6	10.858	8.073	10.761	90.0	90.0	90.0
SCIV8	300	1.7	8.121	10.924	11.115	89.8	94.3	90.0
DCI32	88	0.0	12.163	16.165	21.717	90.2	90.0	90.0
DCI32	300	0.0	12.178	16.243	21.700	90.0	90.0	90.0
DCI32	350	0.0	12.203	16.298	21.729	90.0	90.0	90.0

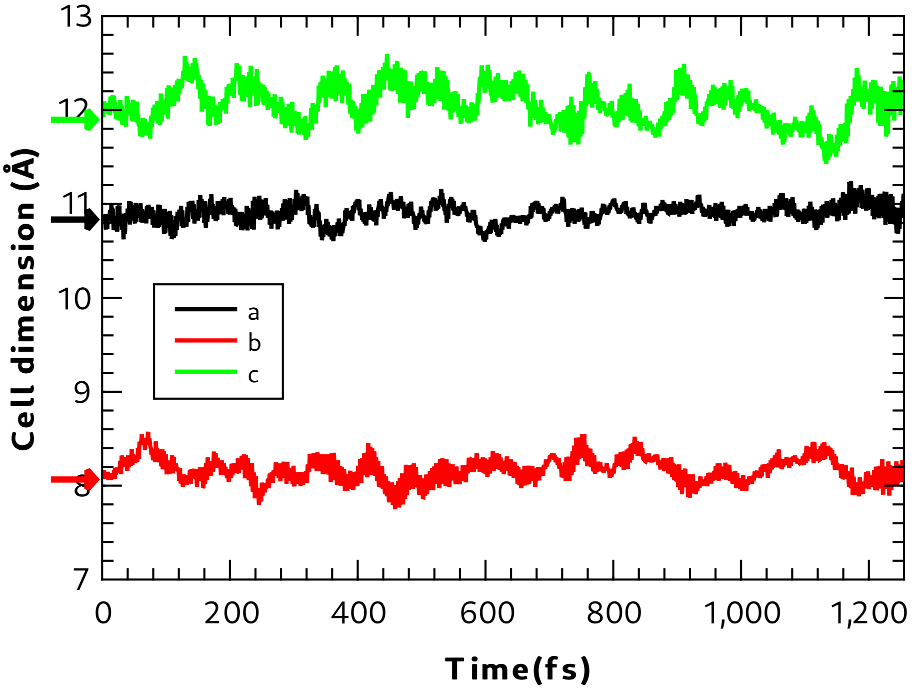

Figure 36: Form I fluctuations in cell dimension during NPT simulations

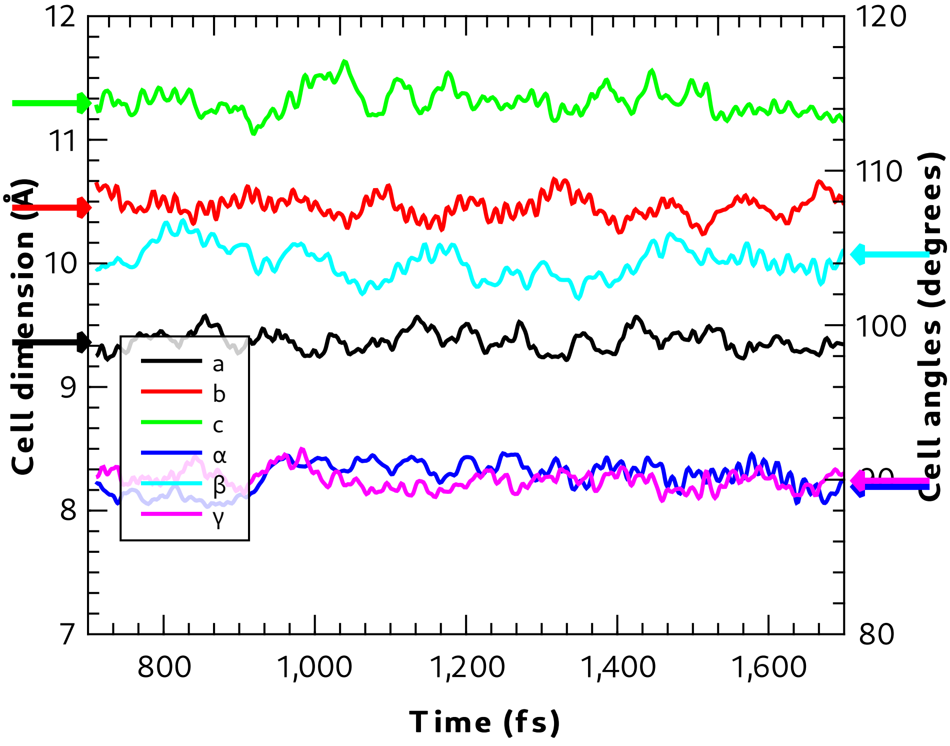

Figure 37: Form II fluctuations in cell dimensions during NPT simulations

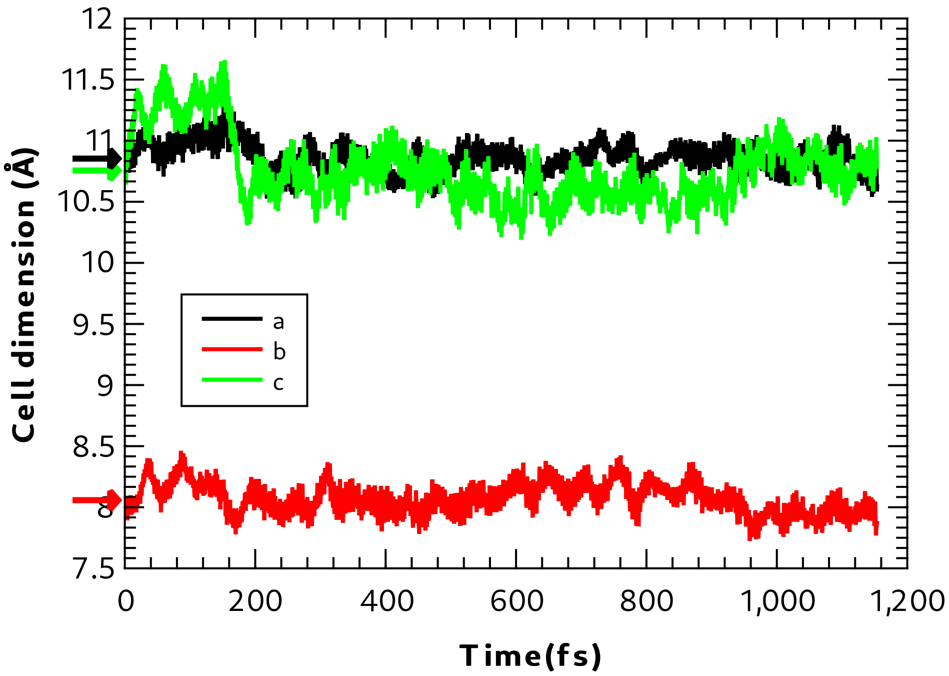

Figure 38: Form III fluctuations in cell dimensions during NPT simulations

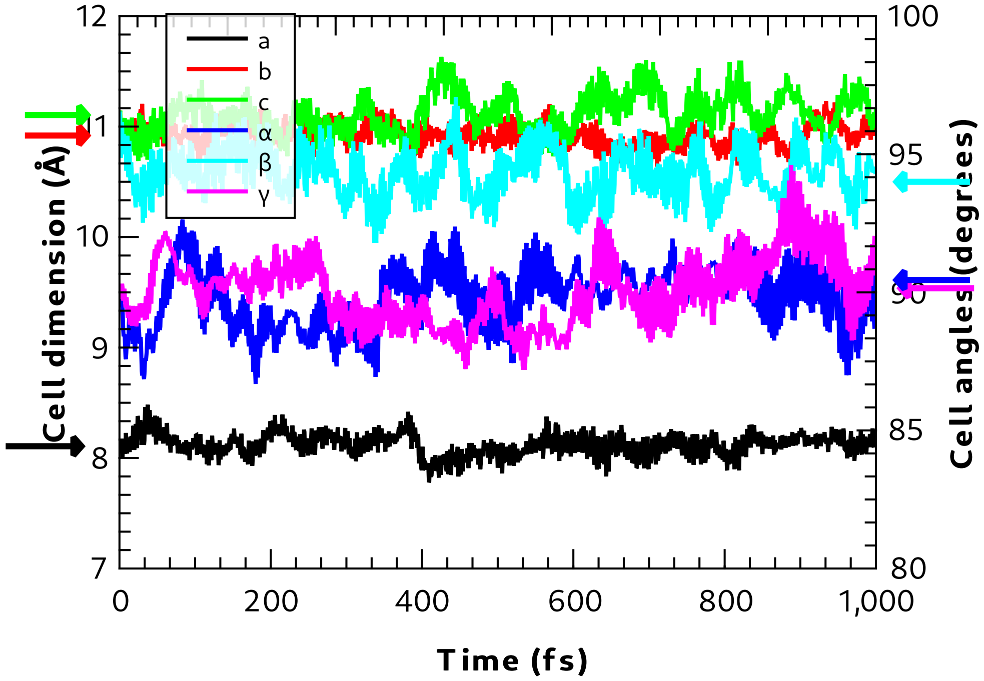

Figure 39: Form IV fluctuations in cell dimensions during NPT simulations

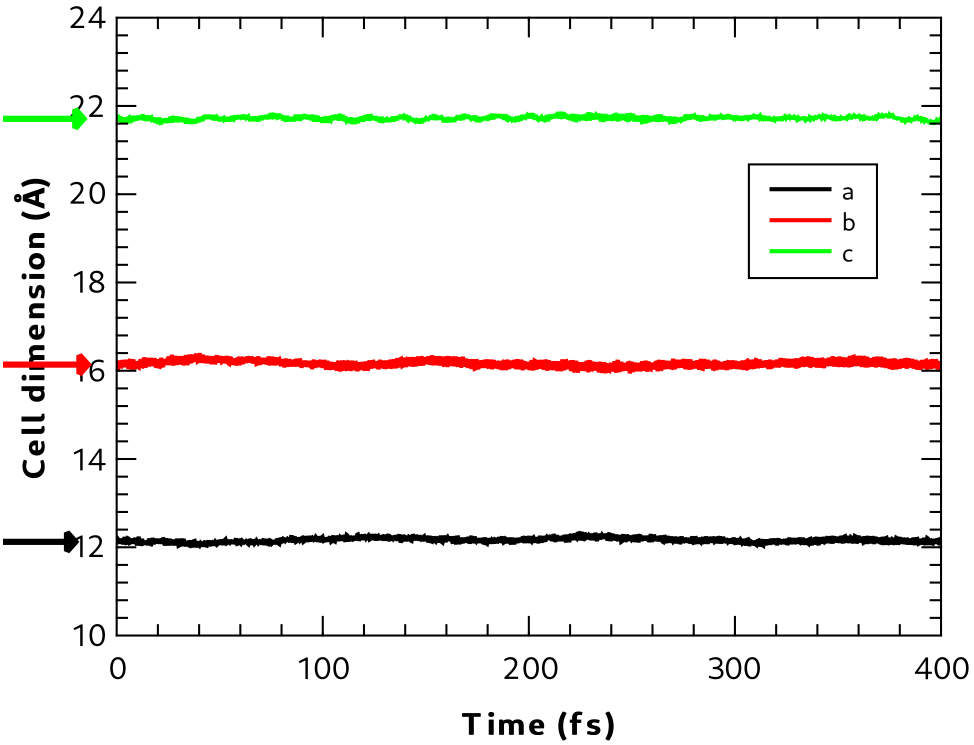

Figure 40: Super-cell DCI32 fluctuations in cell dimensions during NPT simulations at 88 K

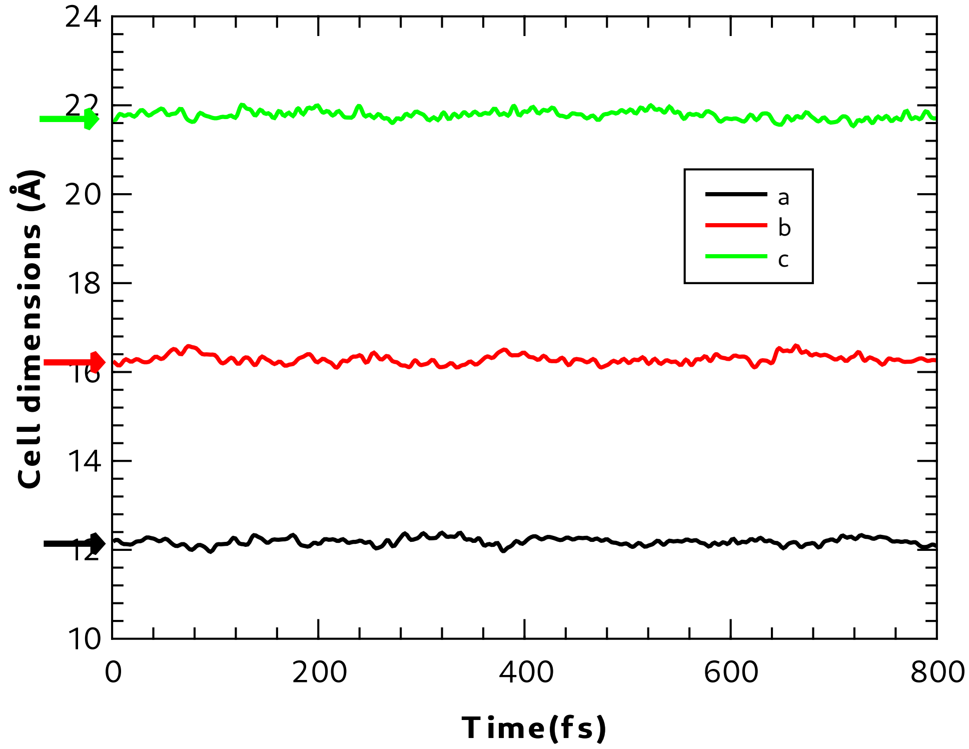

Figure 41: Super-cell DCI32 fluctuations in cell dimensions during NPT simulations at 300 K

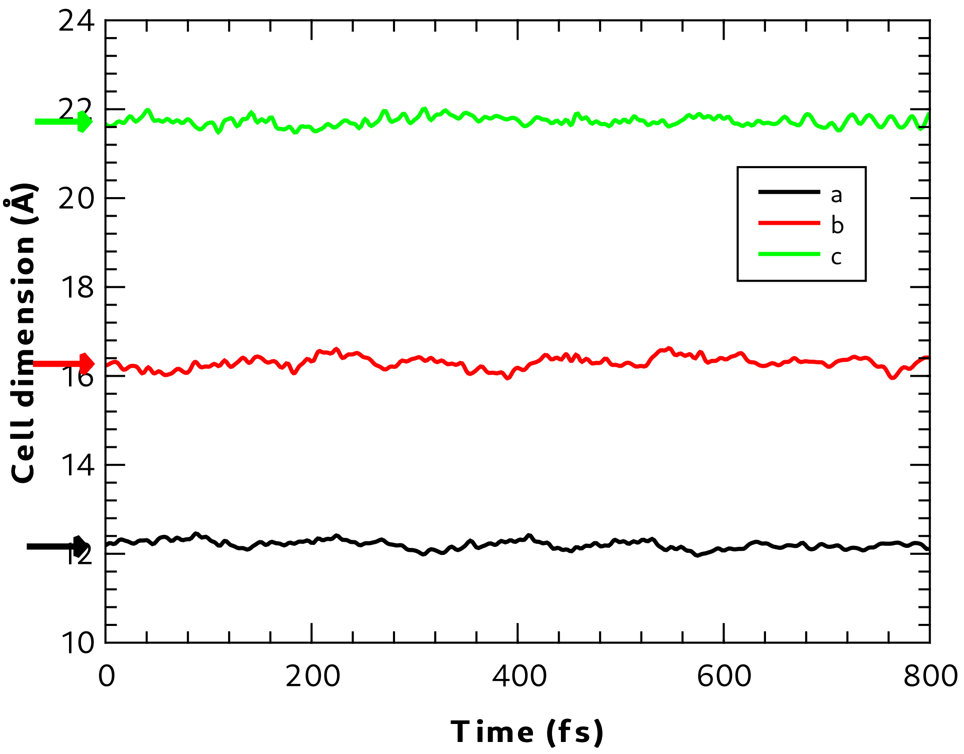

Figure 42: Super-cell DCI32 fluctuations in cell dimensions during NPT simulations at 350 K

8.2 NVT Calculations of Super-cells

NVT calculations were then performed at the average cell dimensions determined from the previous NPT calculations. After equilibration the 30 ps trajectories were analysed to determine the molecular changes in the dipole moment and the C-C-S-H torsion angles using the TRAVIS code.

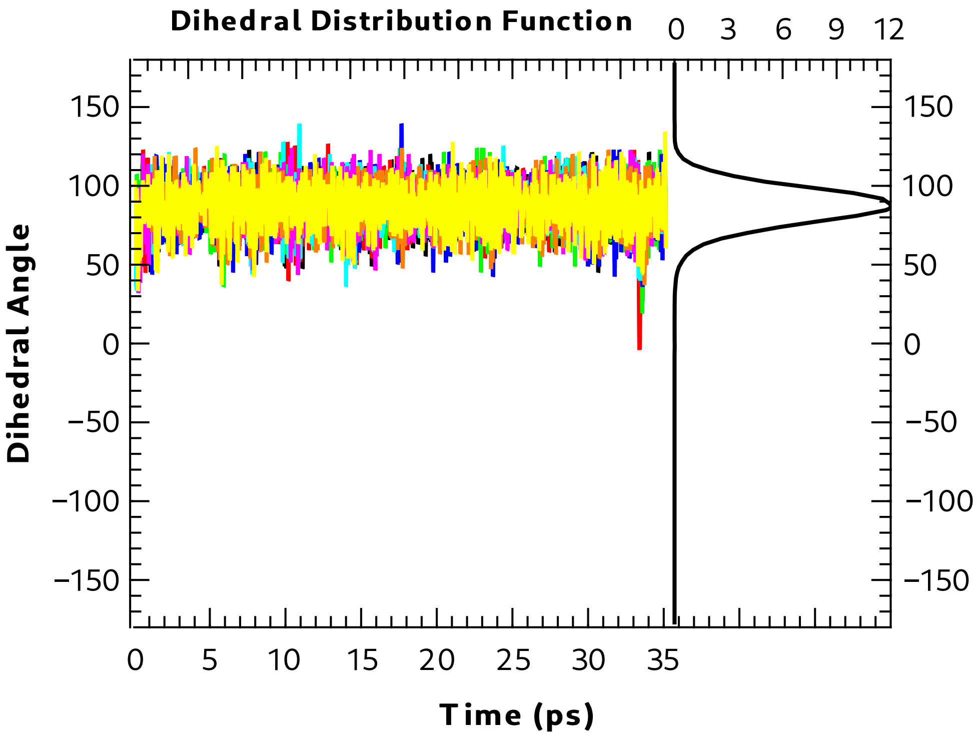

Figure 43: C-C-S-H dihedral angle fluctuations and distribution in Form I at 300 K

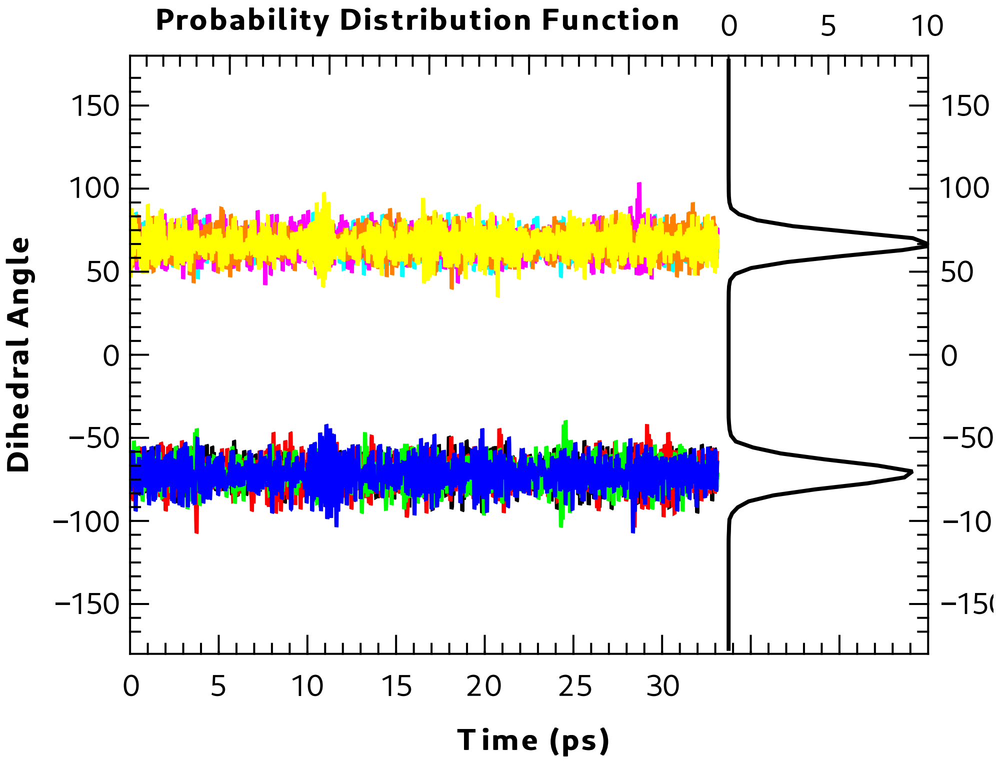

Figure 44: C-C-S-H dihedral angle fluctuations and distribution in Form II at 120 K

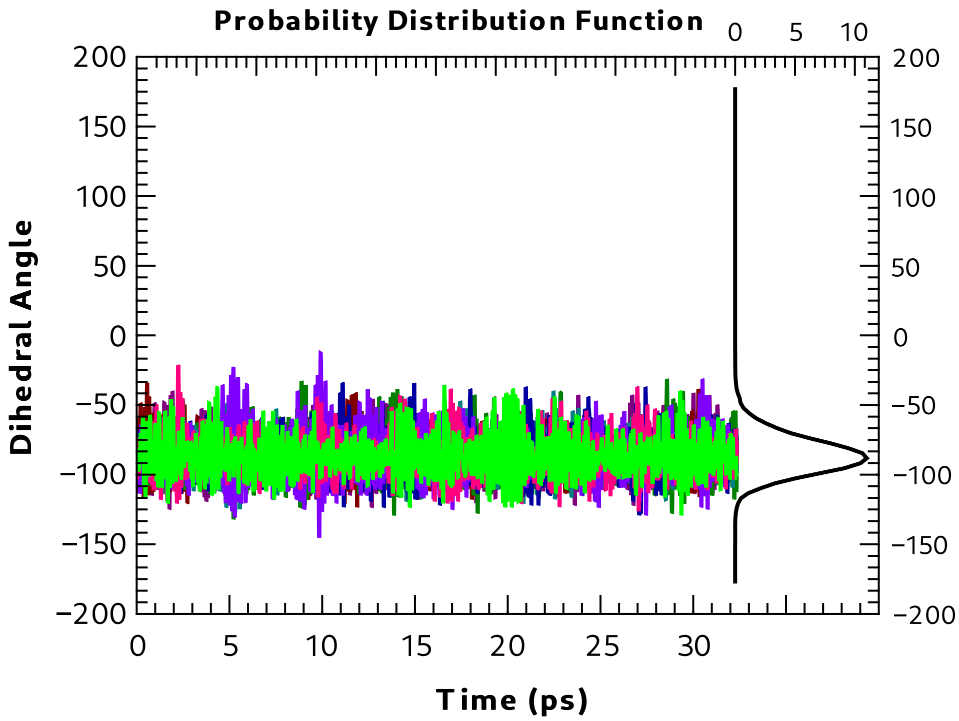

Figure 45: C-C-S-H dihedral angle fluctuations and distribution in Form III at 300 K

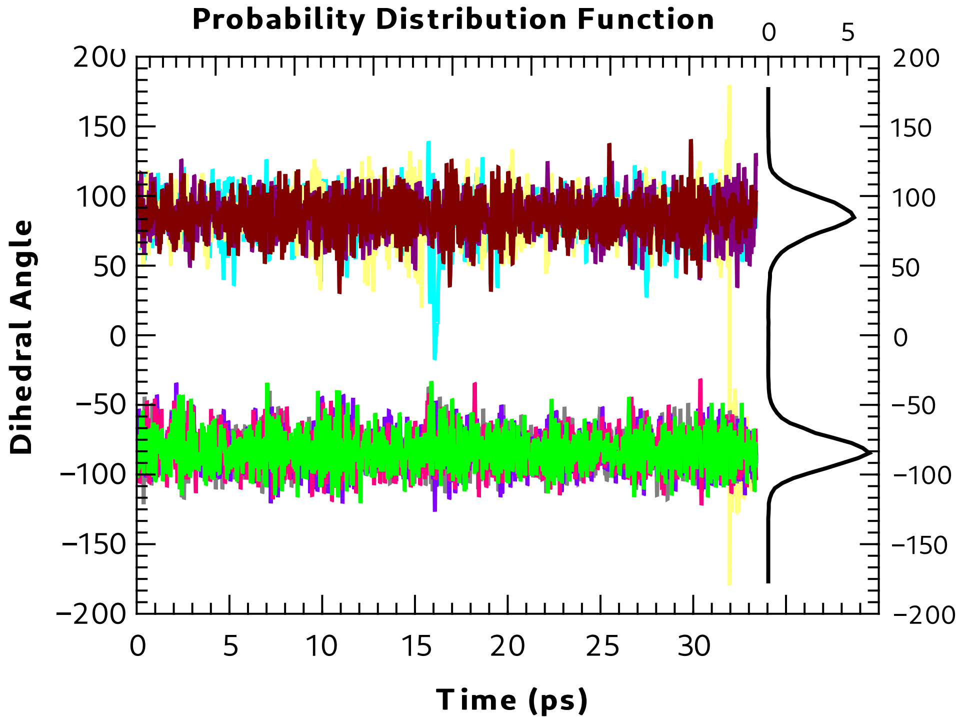

Figure 46: C-C-S-H dihedral angle fluctuations and distribution in Form IV at 300 K

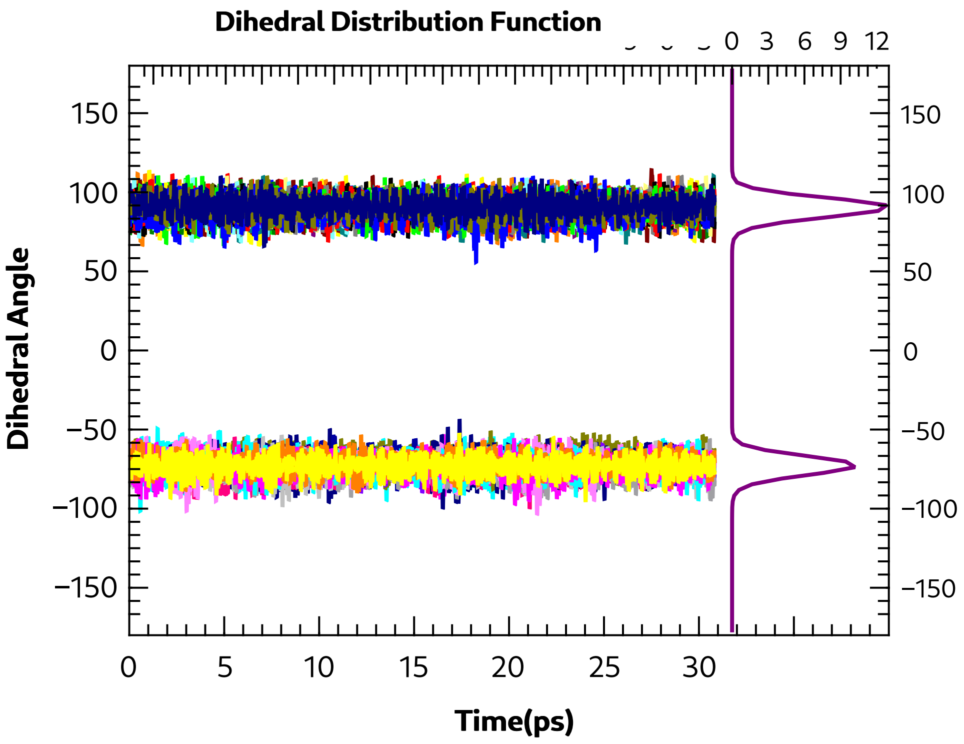

Figure 47: C-C-S-H dihedral angle fluctuations and distribution in DCI32 at 88 K

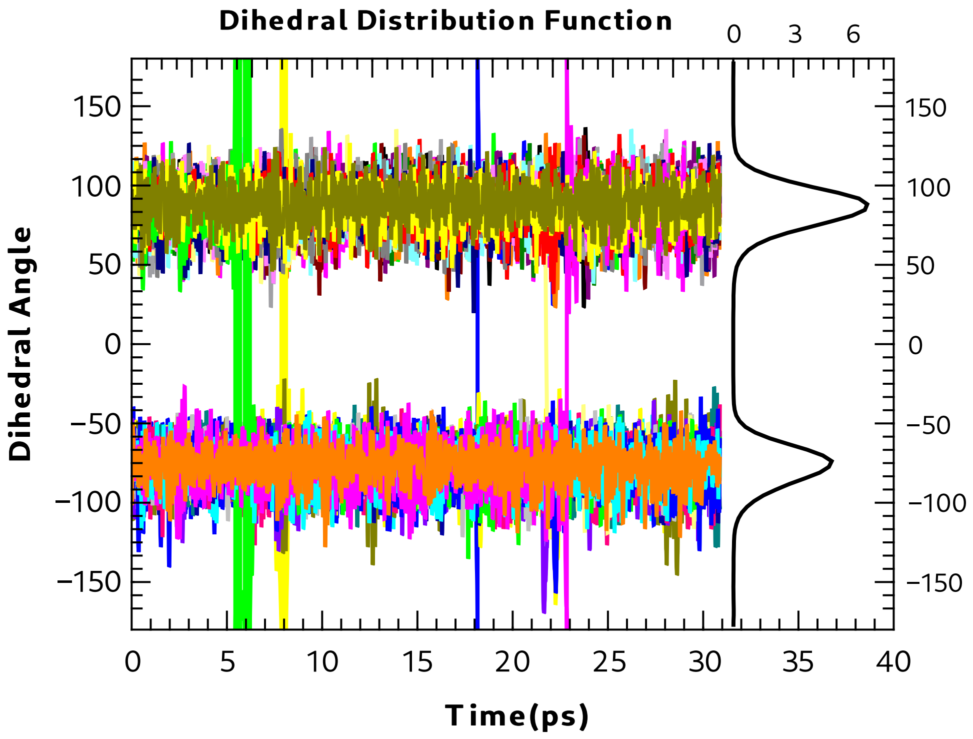

Figure 48: C-C-S-H dihedral angle fluctuations and distribution in DCI32 at 300 K

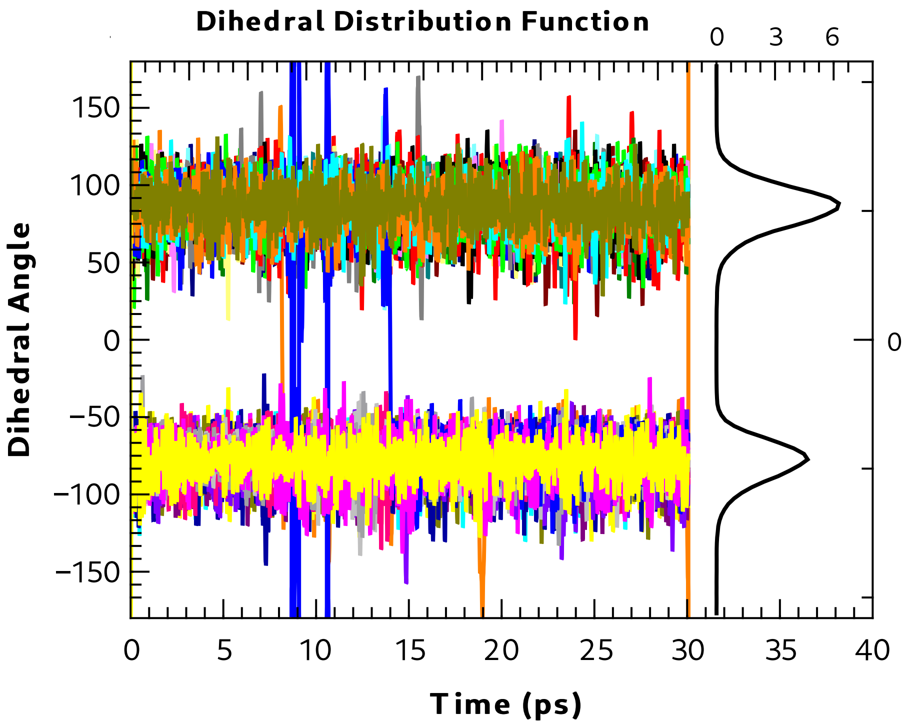

Figure 49: C-C-S-H dihedral angle fluctuations and distribution in DCI32 at 350 K

8.3 IR Absorption from Molecular Dipole Fluctuations

A comparison of the calculated absorption using Travis for the molecular dynamics simulations of the four polymorphs of cysteine are shown in Figures 50 to 53

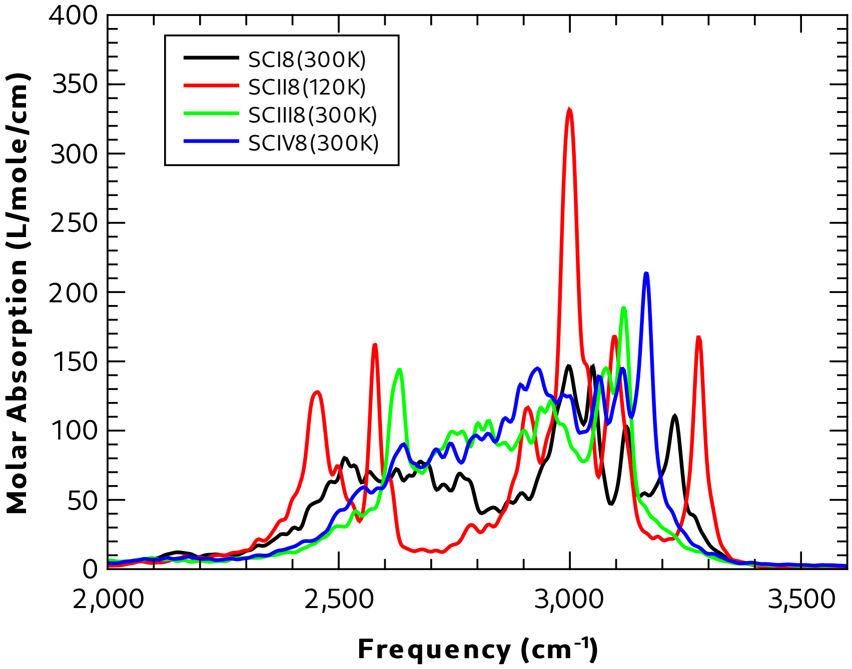

Figure 50: Travis infrared absorption for all polymorphs - high Frequencies

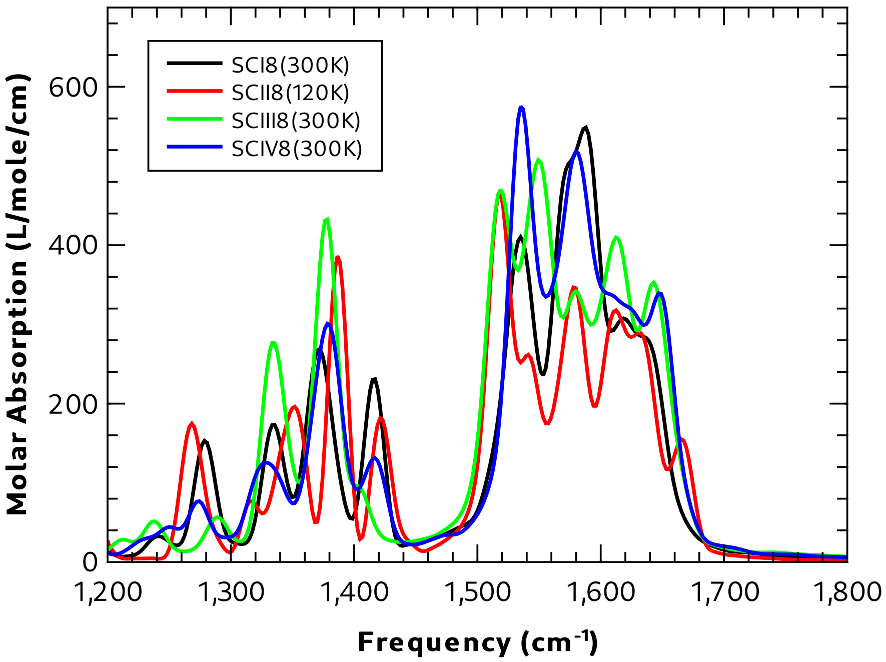

Figure 51: Travis infrared absorption for all polymorphs - Intermediate Frequencies

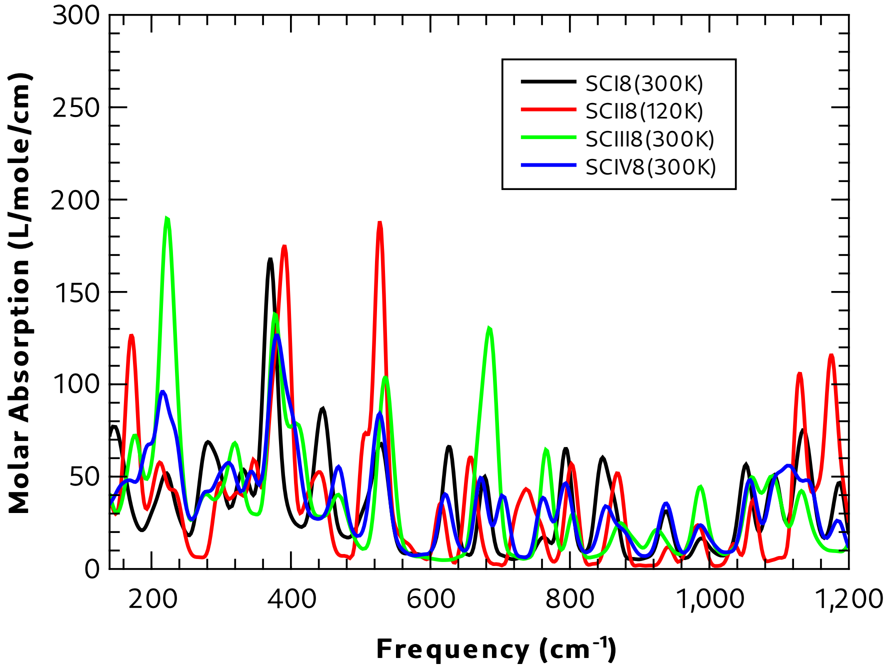

Figure 52: Travis infrared absorption for all polymorphs - low frequencies

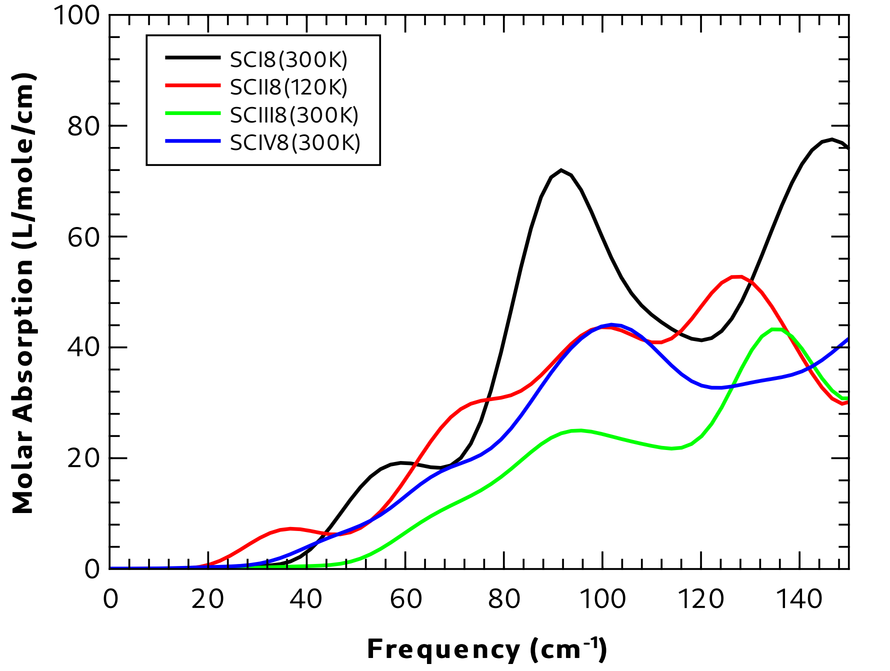

Figure 53: Travis infrared absorption for all polymorphs - THz frequencies

The calculated infrared absorption by Travis for the super-cell DCI32 at temperatures of 88, 300 and 350 K are shown in Figures 54 to 57

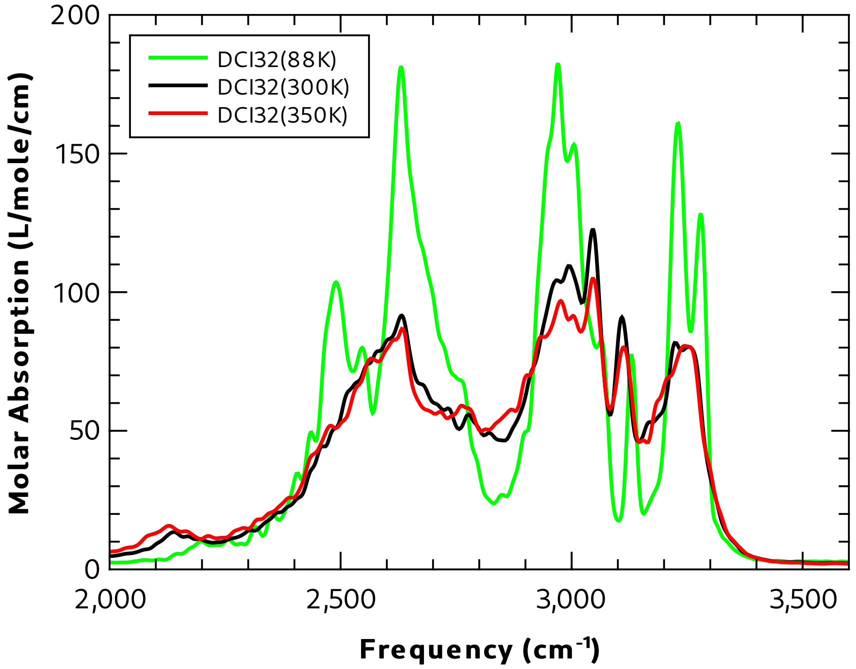

Figure 54: Travis infrared absorption for DCI32 - high frequencies

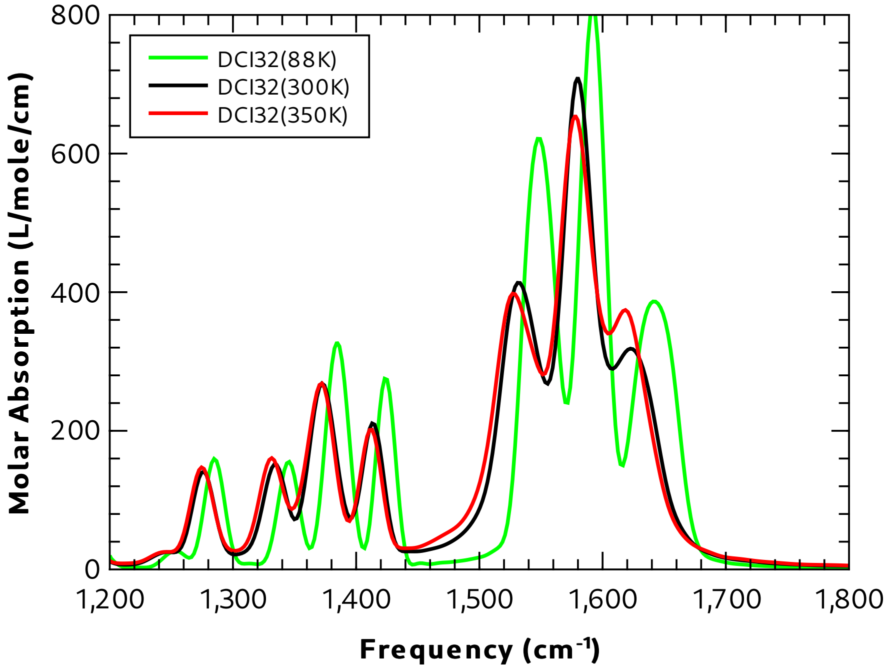

Figure 55: Travis infrared absorption for DCI32 - intermediate frequencies

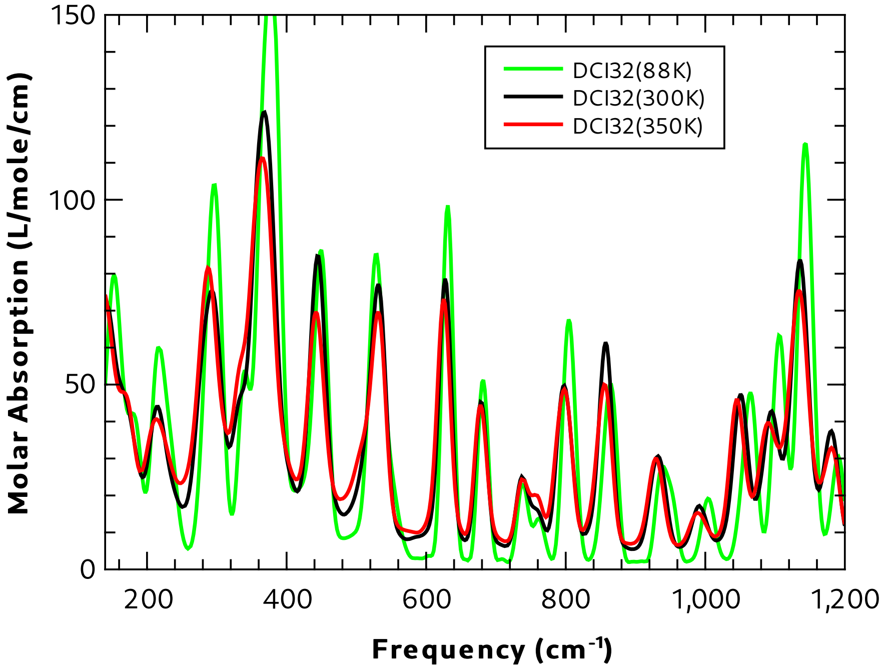

Figure 56: Travis infrared absorption for DCI32 - low frequencies

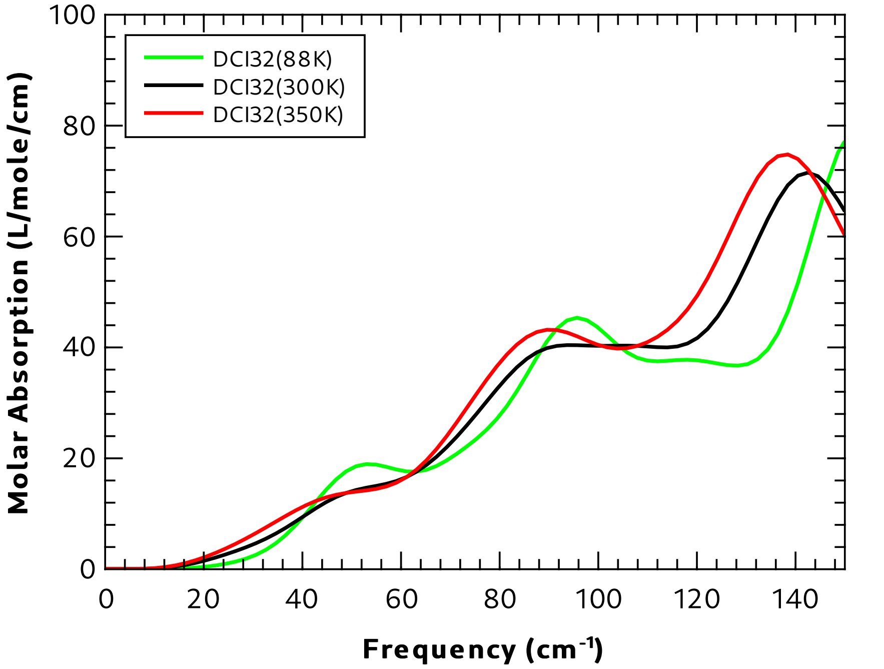

Figure 57: Travis infrared absorption for DCI32 - THz frequencies

8.4 IR Absorption from Cell Dipole Fluctuations

A comparison of the calculated absorption using the super-cell dipole moment from the molecular dynamics simulations of the four polymorphs of cysteine are shown in Figures 50 to 53

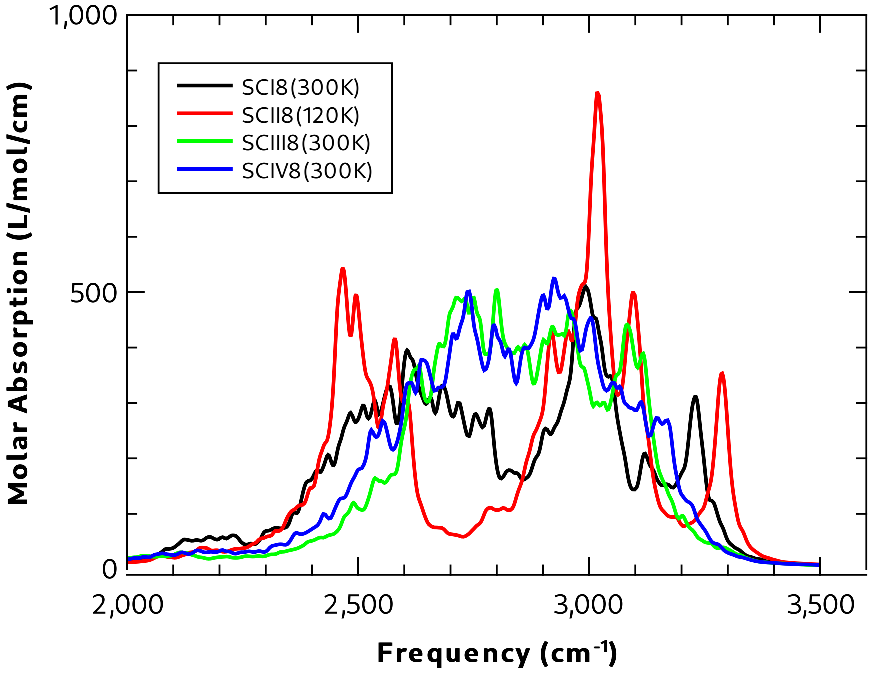

Figure 58: Cell dipole infrared absorption for all polymorphs - high frequencies

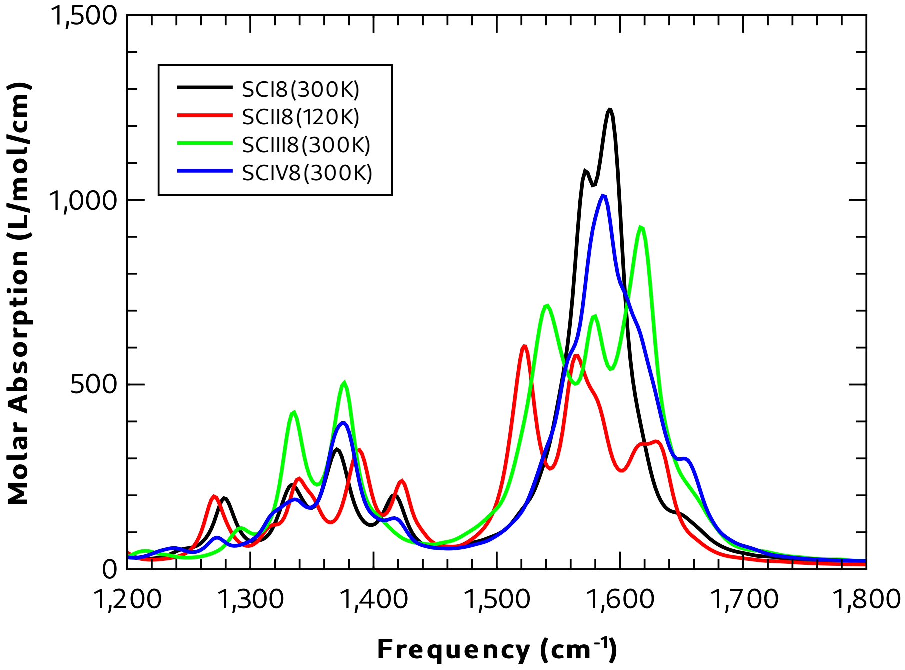

Figure 59: Cell dipole infrared absorption for all polymorphs - intermediate frequencies

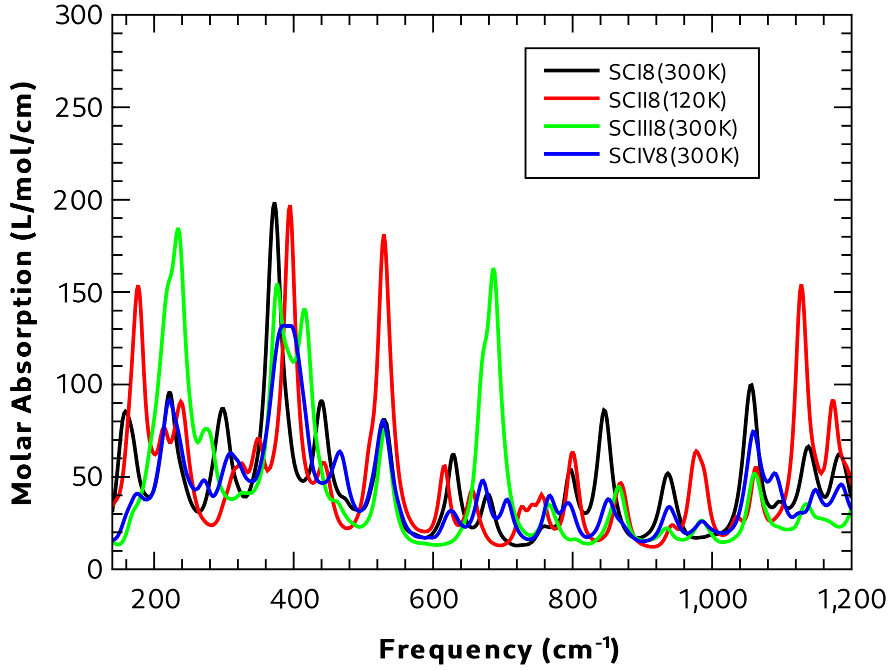

Figure 60: Cell dipole infrared absorption for all polymorphs - low frequencies

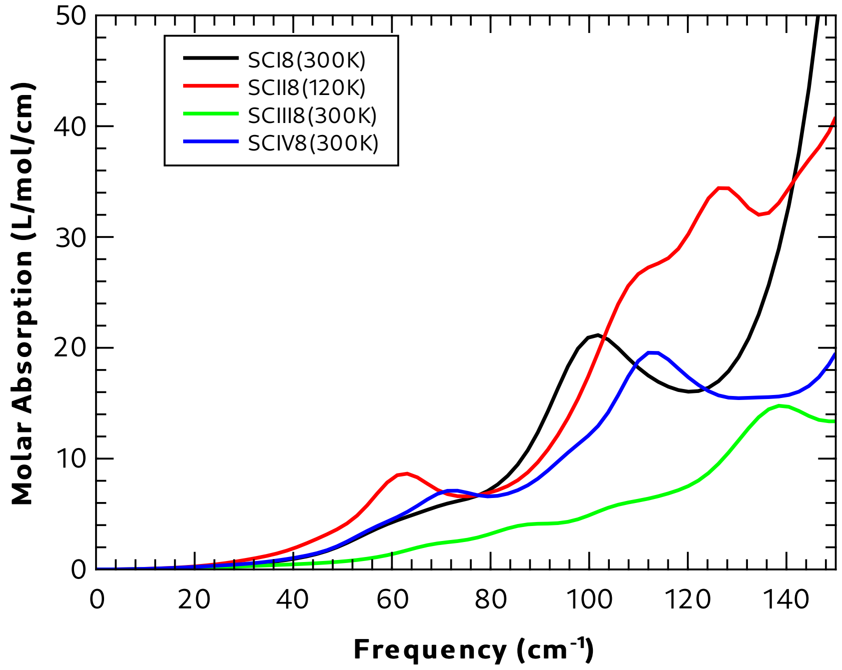

Figure 61: Cell dipole infrared absorption for all polymorphs - THz frequencies

The calculated infrared absorption for the super-cell DCI32 temperatures of 88K, 300K and 350K are shown in Figures 62 to 65

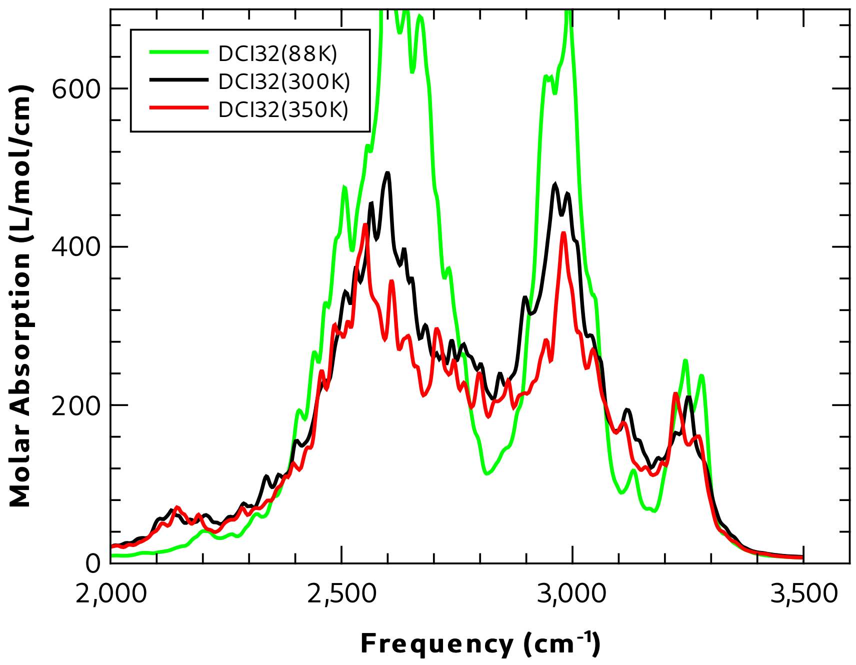

Figure 62: Cell dipole infrared absorption for DCI32 dispersed model - high frequencies

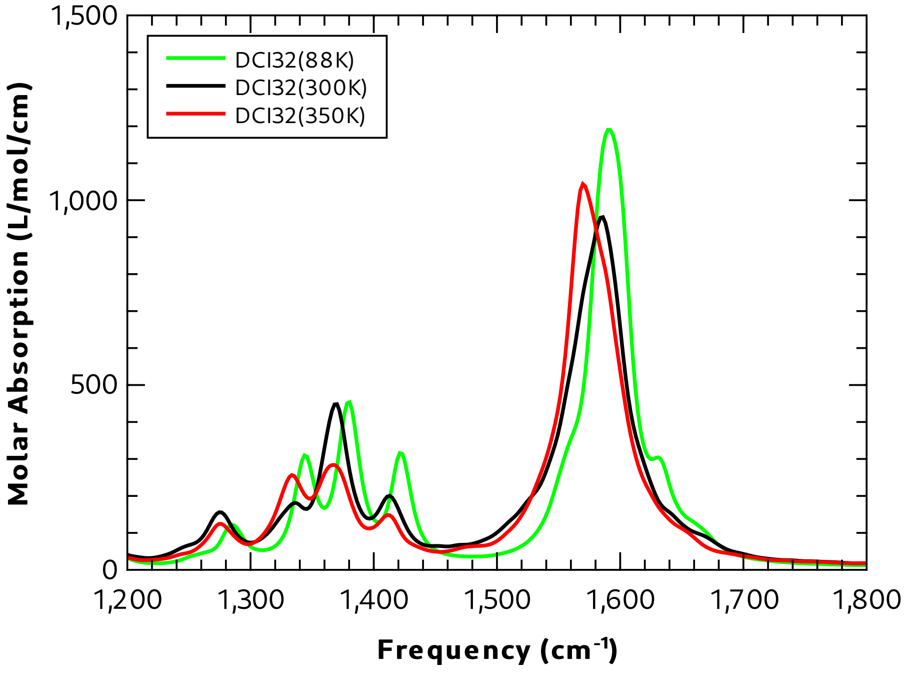

Figure 63: Cell dipole infrared absorption for DCI32 dispersed model - intermediate frequencies

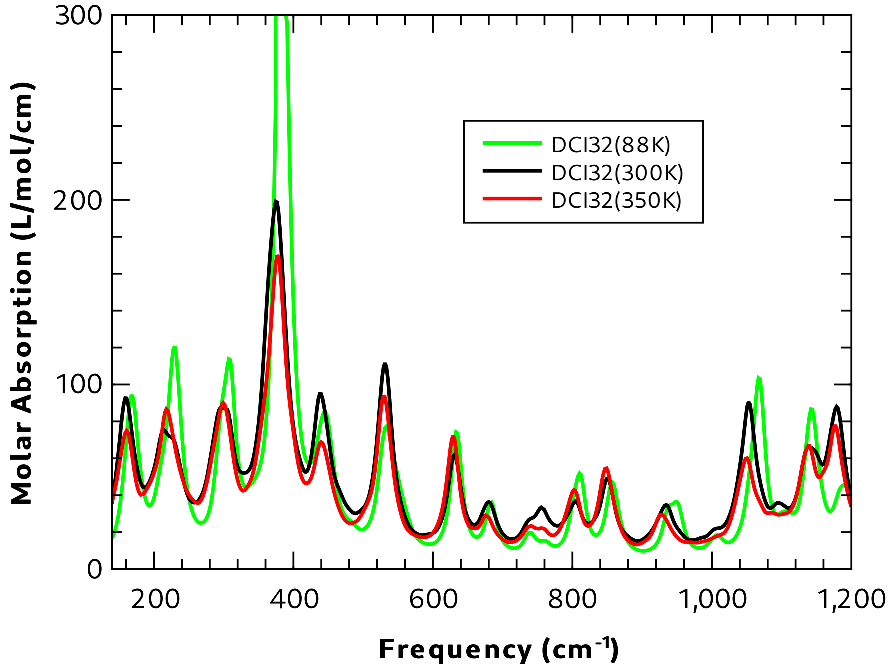

Figure 64: Cell dipole infrared absorption for DCI32 dispersed model - low frequencies

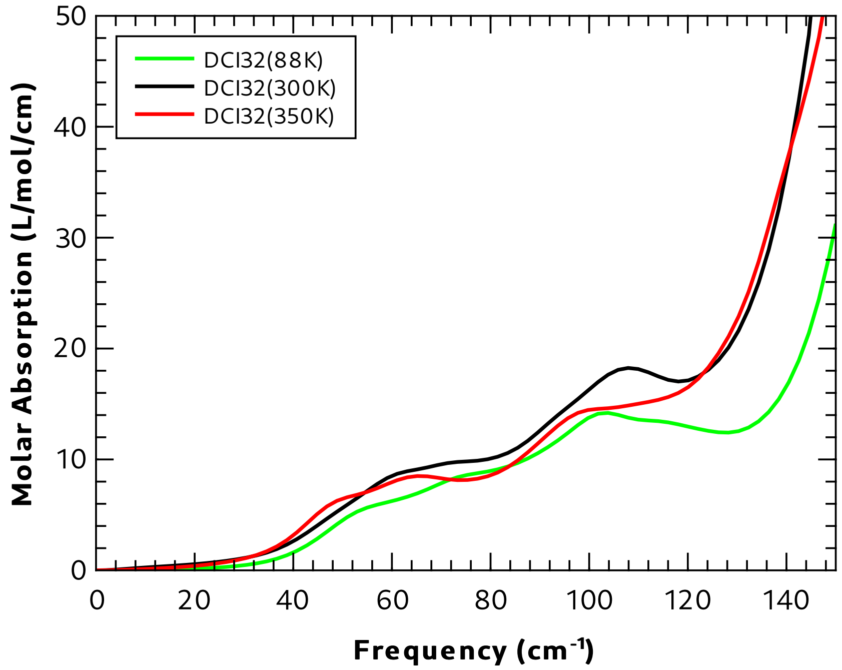

Figure 65: Cell dipole infrared absorption for DCI32 dispersed model - THz frequencies

References

(1) Kolesov, B. A.; Minkov, V. S.; Boldyreva, E. V.; Drebushchak, T. N. Phase Transitions in the Crystals of L and DL-Cysteine on Cooling: Intermolecular Hydrogen Bonds Distortions and the Side-Chain Motions of Thiol-Groups. 1. L-Cysteine. *The Journal of Physical Chemistry B* **2008**, *112*, 12827–12839.

(2) Moggach, S. A.; Allan, D. R.; Clark, S. J.; Gutmann, M. J.; Parsons, S.; Pulham, C. R.; Sawyer, L. High-pressure polymorphism in L-Cysteine: The crystal structures of L-Cysteine-III and L-Cysteine-IV. *Electrochemical and Solid-State Letters* **2006**, *9*, 296–309.

(3) Groom, C. R.; Bruno, I. J.; Lightfoot, M. P.; Ward, S. C. The Cambridge structural database. *Acta Crystallographica Section B: Structural Science, Crystal Engineering and Materials* **2016**, *72*, 171–179.

(4) Kerr, K. A.; Ashmore, J. P.; Koetzle, T. F. A neutron diffraction study of L-Cysteine. *Acta Crystallographica Section B* **1975**, *31*, 2022–2026.

(5) Moggach, S. A.; Clark, S. J.; Parsons, S. L-Cysteine-I at 30 K. *Acta Crystallographica Section E* **2005**, *61*, o2739–o2742.

(6) Görbitz, C. H.; Dalhus, B. L-Cysteine, Monoclinic Form, Redetermination at 120K. *Acta Crystallographica Section C* **1996**, *52*, 1756–1759.

(7) Hafner, J. Ab-initio simulations of materials using VASP: Density-functional theory and beyond. *Journal of Computational Chemistry* **2008**, *29*, 2044–2078.

(8) Perdew, J. P.; Burke, K.; Ernzerhof, M. Generalized Gradient Approximation Made Simple. *Physical Review Letters* **1996**, *77*, 3865–3868.

(9) Blöchl, P. E. Projector augmented-wave method. *Physical Review B* **1994**, *50*, 17953.

(10) Grimme, S.; Ehrlich, S.; Goerigk, L. Effect of the damping function in dispersion corrected density functional theory. *Journal of Computational Chemistry* **2011**, *32*, 1456–1465.

(11) Becke, A. D.; Johnson, E. R. A density-functional model of the dispersion interaction. *The Journal of Chemical Physics* **2005**, *123*, 154101.

(12) Tkatchenko, A.; Scheffler, M. Accurate molecular van der Waals interactions from ground-state electron density and free-atom reference data. *Physical Review Letters* **2009**, *102*, 073005.

(13) Steinmann, S. N.; Corminboeuf, C. A generalized-gradient approximation exchange hole model for dispersion coefficients. *The Journal of Chemical Physics* **2011**, *134*, 044117.

(14) Tkatchenko, A.; DiStasio Jr, R. A.; Car, R.; Scheffler, M. Accurate and efficient method for many-body van der Waals interactions. *Physical review letters* **2012**, *108*, 236402.

(15) Kendrick, J.; Burnett, A. D. Exploring the Reliability of DFT Calculations of the Infrared and Terahertz Spectra of Sodium Peroxodisulfate. *Journal of Infrared, Millimeter, and Terahertz Waves* **2019**,

(16) Bučko, T.; Hafner, J.; Ángyán, J. G. Geometry optimization of periodic systems using internal coordinates. *The Journal of Chemical Physics* **2005**, *122*, 124508.

(17) Bučko, T. Transition state optimization of periodic systems using delocalized internal coordinates. *Theoretical Chemistry Accounts* **2018**, *137*, 1–10.

(18) Togo, A.; Tanaka, I. First principles phonon calculations in materials science. *Scr. Mater.* **2015**, *108*, 1–5.

(19) Kühne, T. D. et al. CP2K: An electronic structure and molecular dynamics software package - Quickstep: Efficient and accurate electronic structure calculations. *Journal of Chemical Physics* **2020**, *152*.

(20) VandeVondele, J.; Hutter, J. Gaussian basis sets for accurate calculations on molecular systems in gas and condensed phases. *Journal of Chemical Physics* **2007**, *127*.

(21) Goedecker, S.; Teter, M. Separable dual-space Gaussian pseudopotentials. *Physical Review B - Condensed Matter and Materials Physics* **1996**, *54*.

(22) Chen, W.; Li, L.-S. The study of the optical phonon frequency of 3C-SiC by molecular dynamics simulations with deep neural network potential. *Journal of Applied Physics* **2021**, *129*, 244104.
